# Sexual life cycle establishes the unicellular red algae Cyanidiophyceae as a genetically tractable model for eukaryotic evolution

**DOI:** 10.1093/plcell/koag148

**Published:** 2026-06-05

**Authors:** Shunsuke Hirooka, Takayuki Fujiwara, Mark Seger, Soichi Inagaki, Shota Yamashita, Dai Tsujino, Ryo Onuma, Yu Kanesaki, Satoru Watanabe, Yuu Hirose, Ryudo Ohbayashi, Mari Takusagawa, Baifeng Zhou, Reiko Tomita, Fumi Yagisawa, Peter Lammers, Atsuko H Iwane, Shin-ya Miyagishima

**Affiliations:** Department of Gene Function and Phenomics, National Institute of Genetics, 1111 Yata, Mishima, Shizuoka 411-8540, Japan; Department of Gene Function and Phenomics, National Institute of Genetics, 1111 Yata, Mishima, Shizuoka 411-8540, Japan; Genetics Program, Graduate University for Advanced Studies (SOKENDAI), 1111 Yata, Mishima, Shizuoka 411-8540, Japan; Arizona Center for Algae Technology and Innovation, Arizona State University, 7418 Innovation Way South, Mesa, AZ 85212, United States; Department of Biological Sciences, Graduate School of Science, The University of Tokyo, 7-3-1 Hongo, Bunkyo-ku, Tokyo 113-0033, Japan; Department of Gene Function and Phenomics, National Institute of Genetics, 1111 Yata, Mishima, Shizuoka 411-8540, Japan; Department of Gene Function and Phenomics, National Institute of Genetics, 1111 Yata, Mishima, Shizuoka 411-8540, Japan; Genetics Program, Graduate University for Advanced Studies (SOKENDAI), 1111 Yata, Mishima, Shizuoka 411-8540, Japan; Department of Marine Biodiversity, Kobe University Research Center for Inland Seas, 2746 Iwaya, Awaji, Hyogo 656-2401, Japan; Department of Laboratory Sciences, Graduate School of Health Sciences, Gunma University, 3-39-22, Showa-machi, Maebashi, Gunma 371-8514, Japan; Department of Bioscience, Tokyo University of Agriculture, 1-1-1 Sakuragaoka, Setagaya-ku, Tokyo 156-8502, Japan; Department of Applied Chemistry and Life Science, Toyohashi University of Technology, 1-1 Hibarigaoka, Tempaku, Toyohashi, Aichi 441-8580, Japan; Department of Biological Sciences, Graduate School of Science, Tokyo Metropolitan University, 1-1 Minami-Osawa, Hachioji, Tokyo 192-0397, Japan; Graduate School of Agricultural and Life Sciences, The Univeraity of Tokyo, 1-1-1 Yayoi, Bunkyo-ku, Tokyo 113-8657, Japan; Department of Gene Function and Phenomics, National Institute of Genetics, 1111 Yata, Mishima, Shizuoka 411-8540, Japan; Department of Gene Function and Phenomics, National Institute of Genetics, 1111 Yata, Mishima, Shizuoka 411-8540, Japan; Research Facility Center, University of the Ryukyus, 1 Senbaru, Nishihara-cho, Nakagami-gun, Okinawa 903-0213, Japan; Arizona Center for Algae Technology and Innovation, Arizona State University, 7418 Innovation Way South, Mesa, AZ 85212, United States; Center for Biosystems Dynamics Research, Laboratory for Cell Field Structure, RIKEN, 3-10-23 Kagamiyama, Higashihiroshima, Hiroshima 739-0046, Japan; Center for Biosystems Dynamics Research, Laboratory for Comprehensive Bioimaging, RIKEN Center for Biosystems Dynamics Research, 2-2-3 Minatojima-minamimachi, Chuo, Kobe, Hyogo 650-0047, Japan; Department of Gene Function and Phenomics, National Institute of Genetics, 1111 Yata, Mishima, Shizuoka 411-8540, Japan; Genetics Program, Graduate University for Advanced Studies (SOKENDAI), 1111 Yata, Mishima, Shizuoka 411-8540, Japan

## Abstract

The thermo-acidophilic unicellular algal class Cyanidiophyceae diverged from other red algae soon after primary chloroplast acquisition. Cyanidiophyceae possess extremely simple genomes (8.7 to 17.8 Mb; approximately 4,800 to 7,800 genes), and the cell wall-less, genetically tractable strain *Cyanidioschyzon merolae* 10D has served as a model organism. However, its unknown sexual life cycle has limited its utility in studies of evolution and genetics. Here, we show that in the genera *Cyanidioschyzon*, *Cyanidiococcus*, and *Cyanidium*, the cell-walled diploid form, exclusively observed in nature, produces a cell wall-less haploid form when the culture pH is lowered, and both proliferate asexually. *Cz. merolae* 10D is a haploid clone that forms a cell-walled diploid through mating with other haploid clones. In addition, we generated high-quality genomic resources with phase-specific transcriptomes, identified a compact candidate mating-type region, and developed genetic manipulation systems using the cell wall-less haploids of these genera. We further uncovered phase-specific distributions of histone H3 lysine 27 trimethylation linked to haploid- and diploid-specific gene expression, including transcription factors involved in differentiation associated with sexual reproduction in plants. Additionally, biparental inheritance of organelle DNA occurs following isogamous mating of haploid cells but resolves into uniparental inheritance during diploid proliferation. These advances position Cyanidiophyceae as a powerful model lineage for studying early Archaeplastida evolution, the shared mechanisms of photosynthetic eukaryotes, and environmental adaptation.

## Teaser

The elucidation of previously unrecognized cryptic sexual reproduction in early-diverging single-celled red algae provides a system to study how photosynthetic organisms evolved and adapted to their environments.

## Introduction

**Figure 1:**
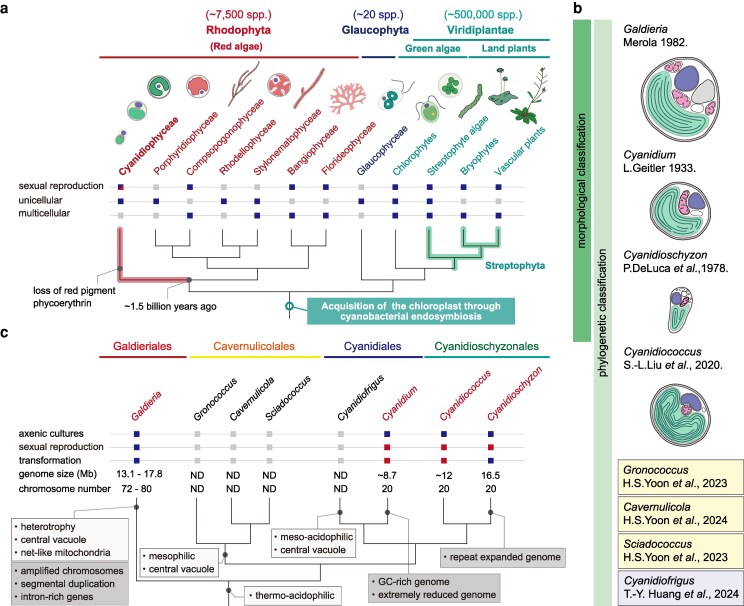
Taxonomy of Cyanidiophyceae. (a) Evolutionary position of the unicellular red algae Cyanidiophyceae within Archaeplastida. The cladogram, based on previous studies (Munoz-Gomez et al. 2017; One Thousand Plant Transcriptomes 2019), indicates whether sexual reproduction has been observed and whether the respective groups are unicellular or multicellular. Blue boxes indicate presence based on previous studies, gray boxes indicate absence (or no reports), and red boxes indicate presence based on both a previous study (Hirooka et al. 2022) and this study. (b) Cyanidiophycean algae are currently classified into 8 genera—*Galdieria*, *Cyanidium*, *Cyanidioschyzon*, *Cyanidiococcus*, *Gronococcus*, *Cavernulicola*, *Sciadococcus*, and *Cyanidiofrigus* (Park et al. 2023; Huang et al. 2024). Illustrations of *Galdieria*, *Cyanidium*, *Cyanidioschyzon*, and *Cyanidiococcus*, created with reference to Merola et al. (1981) and Liu et al. (2020), are shown. (c) The cladogram, based on a previous study (Park et al. 2023; Huang et al. 2024), shows the intergeneric phylogenetic relationships of Cyanidiophyceae, comprising 4 major clades: Galdieriales, Cavernulicolales, Cyanidiales, and Cyanidioschyzonales. Each clade is annotated with captions indicating traits (light gray) and genomic features (dark gray). Also shown are the availability of axenic cultures, the observation of sexual reproduction, genetic tractability, genome size, and chromosome number. Blue boxes indicate presence based on previous studies, gray boxes indicate absence (or no observations), and red boxes indicate presence based on this study.

Eukaryotes have evolved and diversified over more than 2 billion years from a last eukaryotic common ancestor (LECA) that arose through a merger between an archaeon-derived host cell and an α-proteobacterial endosymbiont that ultimately became the mitochondrion (Gu, 1997; Hedges et al. 2001; Richards et al. 2024). After LECA diverged into the ancestors of several eukaryotic supergroups, the common ancestor of the supergroup Archaeplastida acquired a chloroplast through the genetic integration of a cyanobacterial endosymbiont into the host nuclear genome, in a process known as primary endosymbiosis (Mereschkowsky 1905; Martin and Herrmann 1998). This lineage subsequently diverged into Rhodophyta (red algae), Glaucophyta, and Viridiplantae (green algae and land plants) (Fig. 1a) (Bowles et al. 2023). In addition to Archaeplastida, many other eukaryotic lineages also acquired photosynthetic capabilities through secondary endosymbiosis, by genetically integrating unicellular red or green algal endosymbionts into their nuclear genomes (Delwiche 1999). In particular, red algae contributed to the establishment of secondary chloroplasts (plastids) in various eukaryotic groups, such as Stramenopila, Alveolata, Haptista, and Cryptista (Sibbald and Archibald 2020; Strassert et al. 2021).

Several lines of evidence indicate that, in addition to the mitochondrion, LECA already possessed complex eukaryotic features, including several functionally distinct membrane-bound organelles (such as the nuclear envelope, endoplasmic reticulum, peroxisomes, lysosomes, and Golgi apparatus), sophisticated cytoskeletal systems, and a sexual reproduction system (Dacks and Doolittle 2001; Richards et al. 2024). However, during the subsequent evolution and diversification of eukaryotes, each lineage independently developed a range of unique traits, including variations in cell morphology, organelle structures, motility, sex determination, and environmental adaptation (Grosberg and Strathmann 2007; Beukeboom and Perrin 2014; Adl et al. 2019; Fritz-Laylin and Titus 2023). For example, among Archaeplastida, red algae, certain lineages of green algae, and land plants have each independently evolved multicellular sexual life cycles (Fig. 1a) (Bowles et al. 2023). To understand how such traits evolved, it is necessary to conduct not only phenotypic and genotypic comparisons across different lineages but also a variety of comparative functional analyses, including genetic manipulation in model organisms (Fields and Johnston 2005). Nevertheless, when the distribution of available model organisms is considered in light of the phylogenetic diversity of eukaryotes, a substantial bias becomes evident (Hedges 2002; Adl et al. 2019). As a result, our current understanding of eukaryotic evolution remains largely incomplete.

Regarding the situation in Archaeplastida, after the establishment of some angiosperms, such as *Arabidopsis thaliana* as model organisms (Hedges 2002) (vascular plants in Fig. 1a), the moss *Physcomitrium patens* (Rensing et al. 2020) and the liverwort *Marchantia polymorpha* (Kohchi et al. 2021) were later developed and have been used as model systems representing basal land plants to study the evolutionary processes underlying key traits of land plants, especially development and sexual reproduction (bryophytes in Fig. 1a). Tracing further back in the phylogeny, the unicellular green alga *Chlamydomonas reinhardtii* has been extensively studied as a model green alga (Goodenough 2022) (chlorophytes in Fig. 1a). In addition, the green algal order Volvocales, which includes unicellular genus *Chlamydomonas* and multicellular genera with varying cell numbers—such as *Gonium* (16 cells; genetically tractable) (Hanschen et al. 2016) and *Volvox* (over 500 cells; genetically tractable) (Prochnik et al. 2010)—represents a lineage in which multicellularity evolved independently of land plants and has served as a model lineage to study the evolution of multicellularity and its coordination with sexual reproduction (chlorophytes in Fig. 1a). Recently, a procedure for genetic manipulation was developed in *Closterium* (Kawai et al. 2022), a member of the class Zygnematophyceae, which is expected to serve as a unicellular algal model that is evolutionarily closer to land plants than Volvocales or other genetically tractable green algae, such as *Ostreococcus* (Lozano et al. 2014), thus filling the gap between core green algae and land plants (streptophyte algae in Fig. 1a). Tracing further back in the phylogeny, the unicellular red alga *Cyanidioschyzon merolae* (*Cz. merolae*), which belongs to the class Cyanidiophyceae (Fig. 1a), is equipped with advanced genetic manipulation systems, and research has begun using this organism to understand the early evolution of the Archaeplastida (Kuroiwa et al. 2018; Miyagishima and Tanaka 2021), as described below. However, because no sexual reproduction process has been identified in this alga, attempts to understand the early evolution of the sexual life cycle in Archaeplastida inevitably rely on comparisons with organisms outside Archaeplastida, such as model animals and yeasts belonging to the supergroup Opisthokonta (Adl et al. 2019).

The unicellular red algal class Cyanidiophyceae primarily inhabits thermo-acidic environments (pH 0.05 to 5.0, <56 °C) in sulfuric hot springs of volcanic areas worldwide and exhibit a blue–green color because they have lost the red pigment phycoerythrin during evolution, which is present in other red algae (Seckbach 1994). This group is estimated to have branched off from a mesophilic and neutrophilic common ancestor of other unicellular and multicellular red algal lineages early in eukaryotic evolution, approximately 1.5 billion years ago (Yoon et al. 2004). Cyanidiophyceae includes a total of 8 genera, with 4—*Galdieria*, *Cyanidium*, *Cyanidioschyzon*, and *Cyanidiococcus*—being axenically cultured. The group was initially classified into 3 genera (*Galdieria*, *Cyanidium*, and *Cyanidioschyzon*) based on morphological and physiological characteristics (Merola et al. 1981) and was more recently expanded based on genomic sequences and structural features (Liu et al. 2020; Cho et al. 2023) (Fig. 1b). Within Cyanidiophyceae, *Galdieria* is the earliest-diverging lineage, estimated to have diverged approximately 1 billion years ago (Yoon et al. 2004) (Fig. 1c). *Galdieria* cells are spherical (3 to 11 *μ*m in diameter), enclosed by a thick cell wall, and contain a single multilobed chloroplast, a net-like mitochondrion, and a central vacuole (Albertano et al. 2000; Yamashita et al. 2025). They proliferate by undergoing 2 to 5 rounds of successive cell divisions, generating 4 to 32 daughter cells before hatching from the mother cell (Albertano et al. 2000; Jong et al. 2021). Among Cyanidiophyceae, *Galdieria* is unique in its ability to grow photoautotrophically, mixotrophically, and heterotrophically, utilizing more than 50 different carbon sources, unlike other genera that are obligate photoautotrophs (Gross and Schnarrenberger 1995; Barbier et al. 2005). Cells of *Cyanidium* and *Cyanidiococcus*, which are spherical (2 to 5 *μ*m in diameter) and enclosed by a rigid cell wall, are morphologically indistinguishable by optical microscopy, although their genome sizes differ significantly (Fig. 1, b and c) (Cho et al. 2023). These genera proliferate by forming 4 daughter cells through 2 successive divisions before being released from the mother cell (Merola et al. 1981; Jong et al. 2021). In contrast, *Cyanidioschyzon* cells are oval shaped (1.5 to 3.5 *μ*m in length), lack a cell wall, and proliferate by binary fission (Fig. 1b) (Merola et al. 1981). Unlike *Galdieria*, these 3 genera contain a cup-shaped chloroplast, a single disc-shaped mitochondrion, and a few small vacuoles (Merola et al. 1981; Albertano et al. 2000). In addition to the above-mentioned thermo-acidophilic genera, Cyanidiophyceae contains 4 mesophilic genera that have not yet been axenically cultured: *Gronococcus*, *Cavernulicola*, and *Sciadococcus*, which form a monophyletic clade and thrive at moderate temperatures (18 to 25 °C), neutral pH, and low light in a few coastal caves (Fig. 1, b and c) (Park et al. 2023), and another genus, *Cyanidiofrigus*, which is mainly found in nonaquatic microhabitats at moderate temperatures (20 to 30 °C) and low pH conditions (pH 0.5 to 2.5) (Fig. 1, b and c) (Huang et al. 2024). Cells of these mesophilic genera possess a cell wall and a central vacuole and proliferate by forming 4 daughter cells within a mother cell (Park et al. 2023; Huang et al. 2024). Furthermore, *Galdieria phlegrea* Soos, a representative of the crypto-endolithic species belonging to 1 of the 2 major clades of *Galdieria* (Qiu et al. 2013), was isolated from soil samples in Soos National Park in the Czech Republic and exhibits a relatively low optimum growth temperature of ∼30 °C (Gross et al. 2002). These mesophilic genera are thought to have secondarily adapted to mesophilic environments from thermo-acidic environments.

In addition to the characteristics mentioned above—adaptation to thermo-acidic environments and simple intracellular architecture—Cyanidiophyceae are also notable for possessing one of the most compact nuclear genomes among eukaryotes (8.7 to 17.8 Mb; approximately 4,800 to 7,800 protein-coding genes) (Matsuzaki et al. 2004; Nozaki et al. 2007; Qiu et al. 2013; Schonknecht et al. 2013; Rossoni et al. 2019; Liu et al. 2020; Downing et al. 2022; Hirooka et al. 2022; Cho et al. 2023). Their compact genomes are attributed to 2 phases of significant gene loss: one that occurred in the common ancestor of Rhodophyta and another in the common ancestor of Cyanidiophyceae (Qiu et al. 2015). Although the specific causes of these reductions remain unclear, both events may have been driven by the need to reduce energy consumption—as an adaptation to oligotrophic environments in the former case, and in the latter, to reallocate the energy saved through genome reduction toward coping with environmental stress, such as high temperature and low pH (Van Etten et al. 2023). Although the genomes of Cyanidiophyceae are streamlined, they have also acquired ∼1% of their gene inventory through horizontal gene transfer (HGT) from bacteria and archaea, and the majority of these HGT-derived genes are apparently related to adaptation to extreme environments (Schonknecht et al. 2013; Rossoni et al. 2019). Thus, Cyanidiophyceae, comprising 8 genera (Fig. 1c), could serve as an excellent model lineage for understanding the evolution of Archaeplastida and, more broadly, eukaryotes. Furthermore, comparisons among genera will not only help understand the traits and mechanisms possessed by the common ancestor over one billion years ago, but also provide information to distinguish and analyze those that each genus or species has independently evolved.

Among Cyanidiophyceae, a strain of the genus *Cyanidioschyzon*, *Cz. merolae* 10D [16.5 Mb genome; 4,803 protein-coding genes {Matsuzaki et al. 2004; Nozaki et al. 2007}—this number will be revised later in this study], which was isolated from a mixture of algae in sulfuric hot spring water in Italy (Toda et al. 1995), has emerged as a model organism (Kuroiwa et al. 2018; Miyagishima and Tanaka 2021). Owing to the absence of a cell wall—the presence of which hampers the introduction of exogenous DNA—and its highly efficient homologous recombination activity, arbitrary chromosomal loci in this strain can be precisely modified via PEG-mediated transformation (Ohnuma et al. 2008). Based on this technique, several molecular genetic procedures have been developed, including gene knockouts, epitope tagging (Imamura et al. 2009; Fujiwara et al. 2013), selectable marker recycling (Takemura et al. 2018; Bora and Tanaka 2025), inducible gene expression [via heat shock, ammonium-to-nitrate exchange, isopropyl β-D-thiogalactopyranoside {IPTG}, or estradiol] (Sumiya et al. 2014; Fujiwara et al. 2015, 2026), inducible protein knockdown (using rapamycin, IPTG, or estradiol) (Fujiwara et al. 2024, 2026), and CRISPR/Cas9-based genome editing (Tanaka et al. 2021). Taking advantage of these techniques and the simple cellular and genomic content of *Cz. merolae* 10D, the strain has increasingly been used in a wide range of research fields, including organelle division (Miyagishima et al. 2003; Nishida et al. 2003; Yoshida et al. 2010; Imoto et al. 2013), the cell cycle (Miyagishima et al. 2014; Fujiwara et al. 2020), the coordination of these 2 mechanisms (Miyagishima et al. 2012; Sumiya et al. 2016), the photosynthetic apparatus (Krupnik et al. 2013; Nilsson et al. 2014; Nikolova et al. 2017; Pham et al. 2019), epigenetics (Mikulski et al. 2017; Hisanaga et al. 2023), RNA processing (Soma et al. 2007; Stark et al. 2015), responses to environmental changes (Imamura et al. 2009; Kobayashi et al. 2014; Pancha et al. 2019), and industrial applications (Rahman et al. 2017; Hirooka et al. 2020; Yoshida et al. 2021; Seger et al. 2023; Villegas-Valencia et al. 2023). Thus, if present, uncovering sexual reproduction in *Cz. merolae* would greatly expand the range of its applications for understanding eukaryotic evolution and for studies based on genetics.

Regarding this point, we recently identified a sexual reproduction process in *Galdieria* (Hirooka et al. 2022). In *Galdieria*, the previously recognized natural, cell-walled form (Fig. 1b) is diploid and, upon a decrease in the pH of the culture, produces a cell wall-less haploid that is likewise capable of asexual reproduction via mitotic cell division (Hirooka et al. 2022). In addition, we successfully transferred the PEG-mediated genetic manipulation method developed in *Cz. merolae* 10D to *Galdieria partita* by utilizing the cell wall-less haploid (Hirooka et al. 2022). Inspired by these findings and developments, we have explored the possibility that *Cz. merolae* and other members of Cyanidiophyceae also undergo a sexual reproduction process, establishing them as a model lineage equipped with functional genomic techniques for understanding eukaryotic evolution.

Here, we report the sexual life cycles of *Cyanidioschyzon* and 2 other genera of Cyanidiophyceae, *Cyanidiococcus* and *Cyanidium*, in which diploid cells are cell walled and proliferate by forming 4 daughter cells within a mother cell, whereas haploid cells are cell wall-less and proliferate by binary fission. In line with these findings, we also show that *Cz. merolae* 10D (isolated from Sardinia, Italy) is a haploid clone that produces a cell-walled diploid through isogamous mating with a haploid of the opposite mating type (generated from a diploid clone isolated from YNP, United States). Based on these findings, we have established high-quality genomic resources for strains belonging to each genus of Cyanidiophyceae, including complete genome sequences based on haploids without allelic polymorphisms, gene models, and transcriptome datasets encompassing haploid- and diploid-specific genes. In addition, we have identified a compact candidate mating-type region (MTR) in *Cz. merolae* and developed genetic manipulation techniques utilizing the cell wall-less haploids of the respective genera. We have also found changes in the genomic distribution of trimethylation of histone H3 lysine 27 (H3K27me3), a well-known repressive epigenetic mark, between haploid and diploid cells, and these changes correlate with haploid- and diploid-dominant gene expression. Notably, these phase-dominant genes include transcription factors (TFs) belonging to families that, in plants, are involved in diverse developmental processes, including flower formation and transitions in the sexual life cycle. In addition, we observed initial biparental inheritance of mitochondrial and chloroplast DNA upon diploid formation through haploid mating, followed by resolution of heteroplasmy (ie eventual uniparental inheritance) during diploid proliferation. In this way, Cyanidiophyceae—based on the developed datasets, procedures, and observations—has now become a powerful model lineage for understanding the early evolution of Archaeplastida, as well as the fundamental and shared mechanisms and environmental adaptation in photosynthetic eukaryotes.

## Results

### All cultivable members of the Cyanidiophyceae, including the genera *Galdieria*, *Cyanidium*, *Cyanidiococcus*, and *Cyanidioschyzon*, undergo sexual reproduction

*Cz. merolae* was initially identified as the only cell wall-less member of the Cyanidiophyceae that proliferates via binary fission and was described in 1978 (De Luca et al. 1978; Merola et al. 1981). However, since the 2000s, several strains possessing a cell wall—morphologically indistinguishable from *Cyanidium* and *Cyanidiococcus* under optical microscopy—have been isolated and assigned to the *Cyanidioschyzon* clade based on molecular phylogenetic analyses (Fig. 1c) (Toplin et al. 2008; Lee et al. 2015). Conversely, *Cyanidiococcus* possesses a cell wall and releases 4 daughter cells from a mother cell, similar to *Cyanidium* (Fig. 1b), but was recently established as a distinct genus that forms a sister group to *Cyanidioschyzon* (Cyanidioschyzonales in Fig. 1c), rather than to *Cyanidium*, based on molecular phylogenetic analysis (Fig. 1c) (Liu et al. 2020). Subsequently, a strain lacking a cell wall and morphologically resembling *Cz. merolae* was also assigned to the *Cyanidiococcus* clade based on molecular phylogenetic analyses (Fig. 1c) (Cho et al. 2020). Thus, molecular phylogenetic classification and the original classification based on cellular morphological characteristics (ie presence or absence of a cell wall and proliferation by binary fission or by forming 4 daughter cells within a mother cell) have become inconsistent, leading to taxonomic confusion. Under these circumstances, while culturing *Cyanidiococcus yangmingshanensis* (*Cc. yangmingshanensis*) NIES-4659, we occasionally observed ovoid-shaped, smaller cells appearing within the population of originally cell-walled, spherical forms. These cells were morphologically similar to *Cz. merolae* 10D (described later).

**Figure 2:**
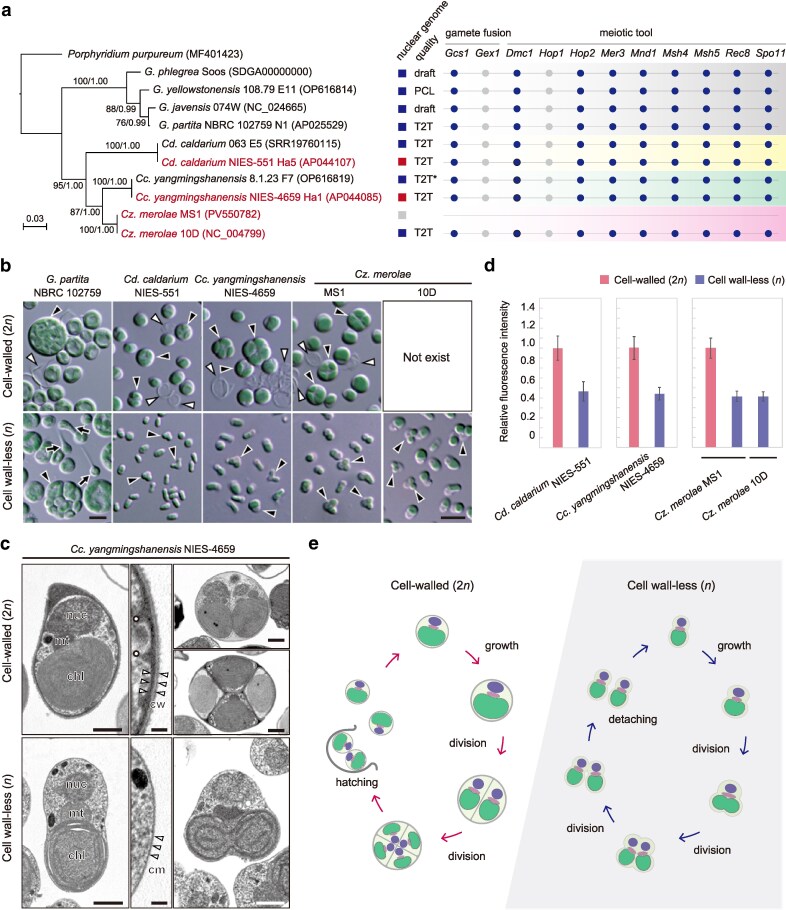
Sexual reproduction in Cyanidiophyceae. (a) Phylogenetic relationships of cyanidiophycean strains based on amino acid sequences of the chloroplast-encoded RbcL protein were inferred using ML analysis. *Porphyridium purpureum* was used as the outgroup. Bootstrap values > 50% (left) obtained by ML analysis and posterior probabilities > 0.95 (right) obtained by Bayesian analysis are shown above the branches. Branch lengths represent evolutionary distances, as indicated by the scale bar. Strains used in this study are highlighted in red. The boxes on the right indicate strains for which genomes have been sequenced to date: blue, sequenced in previous studies; red, sequenced in this study; and gray, not yet sequenced. The quality of assembled genomes was categorized into 3 classes: draft, PCL (pseudochromosome-level), and T2T. An asterisk (*) denotes a genome consisting of 19 T2T contigs and 1 single-end telomere contig (near-complete genome). Presence (blue circles) or absence (gray circles) of putative gamete fusion and meiotic tool genes in the genomes of cyanidiophycean strains (gene IDs or GenBank accession numbers are listed in Data Set S1). (b) DIC micrographs of cell-walled cells (2*n*; original diploid clones) and cell wall-less cells (*n*; haploid clones). Black arrowheads indicate dividing cells; the white arrowhead indicates the mother cell wall released upon hatching of daughter cells; the black arrow indicates tadpole-shaped cells of *G. partita*. Scale bar: 5 *µ*m. (c) Transmission electron micrographs of *Cc. yangmingshanensis* NIES-4659 2*n* and *n* cells. cm, cell membrane; *nuc*, nucleus; *chl*, chloroplast; *cw*, cell wall; *mt*, mitochondrion; white dots, eisosomes. Scale bars: 1 *µ*m (left), 0.1 *µ*m (center), 0.5 *µ*m (right). (d) Relative amounts of nuclear DNA in cell-walled and cell wall-less cells, based on fluorescence intensity of DAPI-stained nuclei. Cells were cultured asynchronously under continuous light, and only nuclei in cell-walled cells at the 4-cell stage (G1 phase just after 2 rounds of cell division) and in ovoid cell wall-less cells (G1 phase) were analyzed. The mean fluorescence intensity of cell-walled cells was defined as 1.0. Data are means ± Sd from 20 independent cells. (e) Schematic illustration of proliferation modes in 2*n* and *n* cells of *Cc. yangmingshanensis*, *Cz. merolae*, and *Cd. caldarium*.

Based on this observation, the apparent discrepancy between cellular morphology and molecular phylogeny, and the recent discovery of a sexual life cycle in *Galdieria* (Hirooka et al. 2022), we hypothesized that previously unrecognized sexual reproduction may also occur in *Cyanidiococcus*, *Cyanidioschyzon*, and even *Cyanidium*. Specifically, we assumed that the cell-walled form may represent the diploid phase, whereas the cell wall-less form may correspond to the haploid phase in these 3 genera. Supporting this hypothesis, a genome survey of these genera revealed the presence of gamete fusion and meiosis-related genes encoded in their genomes (Fig. 2a; Data Set S1).

To test this hypothesis, we prepared 3 cell-walled strains *Cyanidium caldarium* (*Cd. caldarium*) NIES-551, *Cc. yangmingshanensis* NIES-4659, and *Cz. merolae* MS1 (Fig. 2b). Unlike *Cz. merolae* 10D, *Cz. merolae* MS1 is a cell-walled strain that was originally isolated from Yellowstone National Park, United States, and assigned to the *Cyanidioschyzon* clade based on phylogenetic analysis (Fig. 2a). Notably, *Cz. merolae* MS1 was a very rare contaminant in *Galdieria sulphuraria* (*G. sulphuraria*) CCMEE 5587.1 (originally described in Toplin et al. 2008; isolated from Yellowstone National Park). In photoautotrophic cultures of *G. sulphuraria* CCMEE 5587.1 aerated with 2% CO_2_, *Cz. merolae* MS1 grew faster than *G. sulphuraria*, became dominant in the culture, and was subsequently isolated as a clonal strain. The 3 cell-walled strains, maintained in MA medium at pH 2.0, were spherical and proliferated by forming 4 daughter cells within a mother cell (Fig. 2, b and c; Fig. S1, a and b). Based on previous findings that lowering the culture pH from 2.0 to 1.0 triggered a diploid-to-haploid transition in *Galdieria* (Hirooka et al. 2022), we transferred the cultures to lower-pH media—pH 0.75 for *Cd. caldarium* NIES-551 and pH 1.0 for the other strains—and incubated them for 2 to 3 wk. As a result, ovoid, smaller cells resembling *Cz. merolae* 10D appeared among the populations of originally cell-walled cells in all 3 cultures. When these *Cz. merolae*-like cells were isolated and cultured under the same low-pH conditions, they proliferated (Fig. 2b), and transmission electron microscopy confirmed that they lacked a cell wall, in contrast to the original cell-walled cells (Fig. 2c; Fig. S1, a and b). When the nuclear DNA was stained with DAPI and quantified based on fluorescence intensity (Kuroiwa et al. 1986), the cell wall-less forms in each strain showed, as expected, approximately half the DNA content of the original cell-walled forms (Fig. 2d). These results indicate that, as in *Galdieria*, the other genera of Cyanidiophyceae—*Cyanidium*, *Cyanidiococcus*, and *Cyanidioschyzon*—also possess a sexual life cycle, in which both the diploid and haploid are capable of asexual reproduction (Fig. 2e). Thus, it became clear that *Cz. merolae* 10D, which has been used in research to date, should be recognized as being in the haploid phase of the *Cz. merolae* life cycle.

The diploid cells of the 3 genera *Cyanidium*, *Cyanidiococcus*, and *Cyanidioschyzon* are morphologically indistinguishable by optical and transmission electron microscopy, and the same applies to their haploid forms (Fig. 2, b and c; Fig. S1, a and b). As an exception, we found that the cell wall of *Cd. caldarium* NIES-551 diploid cells (∼50 nm thick) was thicker than those of *Cc. yangmingshanensis* NIES-4659 and *Cz. merolae* MS1 diploid cells (∼20 nm thick) (Fig. 2, c and e; Fig. S1). This difference is likely a useful morphological marker to distinguish *Cyanidium* from *Cyanidiococcus* and *Cyanidioschyzon*. However, whether this feature represents a shared derived trait (synapomorphy) of the genus *Cyanidium* requires further investigation of additional strains in the future. Thus, at present, the distinction among *Cyanidium*, *Cyanidiococcus*, and *Cyanidioschyzon* relies primarily on differences in DNA sequences and genome sizes (Cho et al. 2023; Park et al. 2023) (Fig. 1c; Table 1).

**Table 1:** Comparison of genomic features among the cyanidiophycean genera *Galdieria*, *Cyanidium*, *Cyanidiococcus*, and *Cyanidioschyzon*.

Genus	*Galdieria*	*Cyanidium*		*Cyanidiococcus*		*Cyanidioschyzon*
Species name	*G. partita*	*Cd. caldarium*		*Cc. yangmingshanensis*		*Cz. merolae*
Strain name	NBRC 102759 N1	063 E5	NIES-551 Ha5	8.1.23 F7	NIES-4659 Ha1	10D (NIES-3377)
Genome size (Mb)	17.8	8.79	8.73	12.0	12.1	16.5
GC content (%)	37.6	65.7	65.7	54.6	54.6	55.0
No. of contigs	80	20	20	20	20	20
No. of T2T contigs	80	20	20	19	20	20
Telomere sequence	CCCTAA(A)TAAA	AATGGGGGGG	AATGGGGGGG	AATGGGGGG	AATGGGGGG	AATGGGGGG
No. of protein coding genes	7,832	4,870	4,930	4,832	4,884	5,022
No. of introns within ORFs	16,347	44 (41 genes)	46 (42 genes)	36 (35 genes)	35 (34 genes)	34 (33 genes)
No. of 18S, 5.8S, and 28S rRNA units	8	2	2	2	2	3
No. of histone H3 genes	1	3	3	3	3	3
References	Hirooka et al. (2022)	Cho et al. (2023)	This study	Cho et al. (2023)	This study	Matsuzaki et al. (2004), Nozaki et al. (2007), This study

In contrast, the haploid forms of *Cyanidium*, *Cyanidiococcus*, and *Cyanidioschyzon* are also clearly distinct from those of *Galdieria*, as with the known differences in the diploid forms described above: Haploid cells of *Galdieria*, except during cell division, exhibit a tadpole-like shape with an extendable tail in which actin and microtubules are localized (Hirooka et al. 2022), whereas such structures are not observed in the haploid cells of the other 3 genera (Fig. 2b). Furthermore, haploid *Galdieria* cells form colonies of 4 to 32 daughter cells through successive cell divisions before the daughter cells detach from one another (Hirooka et al. 2022), while haploid cells of the other 3 genera proliferate by binary fission (Fig. 2, b and e).

### Development of genomic resources for Cyanidiophyceae enabling intergeneric comparisons and identification of meiotic recombination and genetic diversity

To advance evolutionary and functional genomic analyses in Cyanidiophyceae, high-quality genome data with accurate gene annotation are essential. Although telomere-to-telomere (T2T) or near-complete genome assemblies have recently been established for representative strains of each genus except *Galdieria* (Fig. 2a) (Matsuzaki et al. 2004; Nozaki et al. 2007; Cho et al. 2023), gene prediction in these datasets was based solely on transcriptomic data from either the diploid or haploid phase. However, as shown below, certain genes are specifically expressed in either the haploid or diploid phase, and incorporating transcriptome data from both phases would enable more accurate genome annotation. In this study, we constructed T2T genome assemblies with gene annotations for haploid clones of *Cd. caldarium* NIES-551 Ha5 and *Cc. yangmingshanensis* NIES-4659 Ha1 and also reannotated the genome of *Cz. merolae* 10D.

The assembled nuclear genome sizes of *Cd. caldarium* NIES-551 Ha5 and *Cc. yangmingshanensis* NIES-4659 Ha1 are 8.73 Mb and 12.1 Mb, respectively (Table 1). These sizes are mostly consistent with those reported in previous studies on other strains of these species (*Cd. caldarium* 063 E5 and *Cc. yangmingshanensis* 8.1.23 F7) (Cho et al. 2023) (Table 1), as well as with the results of pulsed-field gel electrophoresis of chromosomes (Fig. S2). The smaller genome sizes of these 2 genera compared to *Cz. merolae* 10D (16.5 Mb) are attributed to the shorter length of intergenic regions rather than to differences in the number of genes as previously shown (Cho et al. 2023). We predicted gene models by incorporating RNA-seq data from both diploid and haploid phases and identified 4,930 protein-coding genes in the nuclear genome of *Cd. caldarium* NIES-551 Ha5, of which 38 genes contained a single intron and 4 genes contained 2 introns within their ORFs, and 4,884 protein-coding genes in the nuclear genome of *Cc. yangmingshanensis* NIES-4659 Ha1, of which 34 genes contained a single intron and 1 gene contained 2 introns (Table 1). These gene numbers are greater than the previous predictions for other strains of these species, which identified 4,870 and 4,832 protein-coding genes, respectively (Table 1) (Cho et al. 2023).

In *Cz. merolae* 10D, previously published gene models were based on evidence from ESTs and amino acid sequence similarity to proteins from other organisms in the GenBank nonredundant (nr) database (Matsuzaki et al. 2004). This set of gene models, consisting of 4,803 protein-coding genes, has not been updated since 2007 (https://www.ncbi.nlm.nih.gov/datasets/genome/GCF_000091205.1/) and contains several annotation errors. Recently, 11 previously unannotated introns were identified in the *Cz. merolae* 10D genome through RNA-seq analysis (Wong et al. 2023), and the start codons of 161 genes were revised using ribosome profiling (Ribo-seq) (Mogi et al. 2025). Furthermore, in the present study, we identified an additional 219 protein-coding genes using RNA-seq data from *Cz. merolae* 10D and from both the diploid and haploid phases of *Cz. merolae* MS1, as well as predicted protein data from *Cc. yangmingshanensis*. As a result, we obtained a reannotated set of 5,022 protein-coding genes, of which 33 genes contained a single intron and 1 gene contained 2 introns (Table 1; Data Set S2).

Regarding *Galdieria*, genome sequences of more than 10 strains are currently available; however, none of them have been assembled at the chromosome level. Among them, recent advanced genomic studies have generated near-chromosome-level assemblies for diploid strains of *Galdieria* spp., including ACUF 138, ACUF 017, SAG 107.79, and 108.79 E11 (Downing et al. 2022; Cho et al. 2023). However, due to extensive segmental duplications across chromosomes, generating T2T genome assemblies from diploid strains remains challenging. To overcome this difficulty, we used a haploid clone to assemble a 17.8 Mb T2T genome of *G. partita* NBRC 102759 N1, which contains 7,832 protein-coding genes with an intron-rich gene structure (16,347 introns; Table 1) (Hirooka et al. 2022).

**Figure 3:**
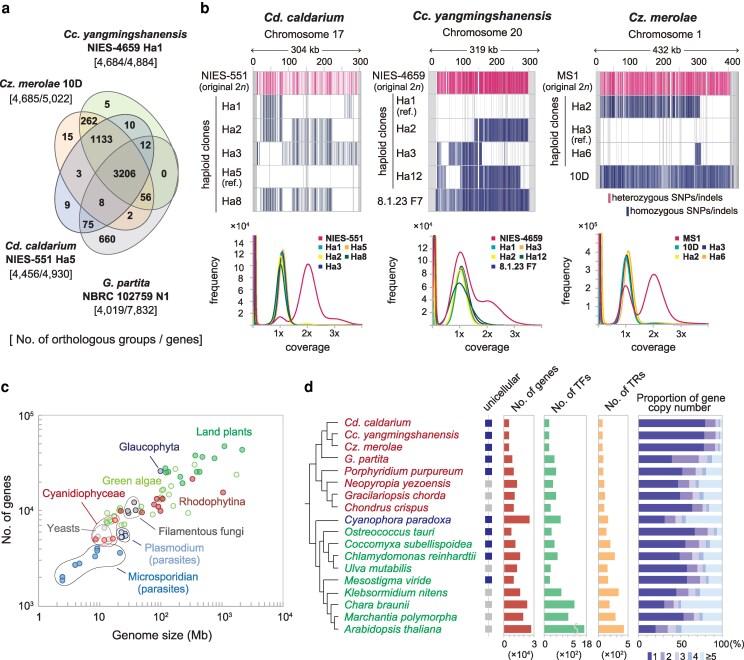
Comparison of genomic features of Cyanidiophyceae with other eukaryotes. (a) Venn diagram showing the number of orthogroups (identified by OrthoFinder; genes duplicated within respective genomes were counted as one) shared among the genomes of *G. partita* NBRC102759 N1, *Cd. caldarium* NIES-551 Ha5, *Cc. yangmingshanensis* NIES-4659 Ha1, and *Cz. merolae* 10D. (b) Mapping of SNP/indel positions in the original 2*n* clones and their derived *n* clones of cyanidiophycean algae. These analyses were restricted to callable regions, defined as genomic positions with sufficient coverage and mapping quality. Sites matching the haploid reference genome sequences are shown in white; heterozygous SNPs/indels in red; homozygous SNPs/indels in blue; and regions not used for variant calling—including regions with highly similar sequences across chromosomes, such as chromosome termini—are shown in gray. Histogram of *k*-mer counts (*k* = 41) was generated from whole-genome Illumina short reads using Jellyfish ver. 2.3.0 (Marcais and Kingsford 2011). (c) Comparison of the number of protein-coding genes and genome sizes of cyanidiophycean algae with those of other eukaryotes. (d) Comparison of the number of protein-coding genes, TFs, and TRs, as well as the proportion of gene copy numbers in Archaeplastida. The proportions of gene copy numbers were calculated from the Orthogroups. GeneCount.tsv file generated by OrthoFinder (Emms and Kelly 2019). The dendrogram with species names on the left is based on previous studies (Munoz-Gomez et al. 2017; One Thousand Plant Transcriptomes 2019). See also Data Set S4 for details.

Then, we compared gene content across the 4 genera of Cyanidiophyceae. Because each genome contains groups of duplicated genes, these were counted as a single orthogroup per species using OrthoFinder (Emms and Kelly 2019), and interspecies comparisons were performed (Fig. 3a). Here, an orthogroup is defined as a group that includes both orthologs, which descended from a single gene in their last common ancestor, and paralogs, which arose from gene duplication events within a species after speciation. The comparative analysis revealed a total of 5,456 orthogroups in the Cyanidiophyceae pangenome, of which 3,206 (58.76%) were shared among all 4 genera. *Cd. caldarium*, *Cc. yangmingshanensis*, and *Cz. merolae* shared 4,339 orthogroups (90.47%) out of the 4,796 identified in those 3 strains (Fig. 3a). Of the total 5,456 orthogroups in Cyanidiophyceae, 660 were uniquely present in *G. partita*, whereas 1,133 were shared exclusively by the other 3 strains (*Cd. caldarium*, *Cc. yangmingshanensis*, and *Cz. merolae*) and were absent in *G. partita* (Fig. 3a). These results indicate that the most pronounced divergence in genomic content exists between *Galdieria* and the other 3 genera—*Cyanidium*, *Cyanidiococcus*, and *Cyanidioschyzon*—which is consistent with their differences in cellular morphology (Fig. 2b) and phylogenetic relationships (Fig. 1c; Fig. 2a). Notably, although *Cd. caldarium* diverged from the common ancestor of *Cc. yangmingshanensis* and *Cz. merolae* approximately 0.3 billion years ago and their genome sizes and GC contents have diverged (Cho et al. 2023), most intron-containing genes are still shared among the 3 genera (Data Set S3), suggesting that these introns play essential roles in their biological processes.

Using the genome assemblies generated in this study, we examined whether meiotic recombination occurs in *Cyanidium*, *Cyanidiococcus*, and *Cyanidioschyzon*, as previously observed in *Galdieria* (Hirooka et al. 2022), by analyzing single nucleotide polymorphisms (SNPs) and insertions/deletions (indels) in haploid clones derived from the original diploid clones. To facilitate this analysis, we additionally constructed a T2T genome assembly of the haploid clone *Cz. merolae* MS1 Ha3, with a genome size of 16.5 Mb. Numerous heterozygous SNPs and indels were detected when genomic reads from the original diploid clones (*Cd. caldarium* NIES-551, *Cc. yangmingshanensis* NIES-4659, and *Cz. merolae* MS1) were mapped to their respective reference genomes, indicating that each clone is a heterozygous diploid (Fig. 3b). Subsequently, when genomic reads from multiple distinct haploid clones derived from each original diploid clone were mapped to the corresponding reference genome, different SNP/indel patterns were observed depending on the haploid clone (Fig. 3b). Furthermore, *k*-mer analysis of genomic reads from the original diploid clones revealed 2 major peaks: one corresponding to heterozygous *k*-mers (with a peak at 1× coverage) and the other to homozygous *k*-mers (with a peak at 2× coverage) (Fig. 3b). In contrast, genomic reads from haploid clones exhibited a single peak (Fig. 3b). These results demonstrate, at the whole-genome level, that meiotic recombination occurs in these algae.

To gain insights into intraspecific genetic diversity within Cyanidiophyceae, we performed intraspecific comparisons of chromosomal architecture and genome-wide sequence divergence. First, we compared synteny within each genus using T2T genome assemblies of 2 strains each of *Cd. caldarium*, *Cc. yangmingshanensis*, and *Cz. merolae*. Overall, synteny was well conserved between strains within each species (Fig. S3). However, between *Cc. yangmingshanensis* 8.1.23 F7 (Kula, Turkey) and NIES-4659 Ha1 (Hakone, Japan), a few inversions were detected. Furthermore, between *Cz. merolae* 10D (Sardinia, Italy) and MS1 Ha3 (YNP, United States), multiple rearrangements, including 1 inversion and 1 reciprocal translocation, were observed. In contrast, between *Cd. caldarium* 063 E5 (Comitini, Italy) and NIES-551 Ha5 (unknown origin), no chromosomal rearrangements were detected.

Next, we quantified sequence divergence between the 2 haplotypes of *Cd. caldarium* NIES-551, *Cc. yangmingshanensis* NIES-4659, and *Cz. merolae* MS1, based on the SNP and indel analyses described above (Fig. 3b). Genome-wide heterozygosity, defined as the SNP/indel rate between the 2 haplotypes within each diploid strain, was 0.093% for *Cd. caldarium* NIES-551, 2.19% for *Cc. yangmingshanensis* NIES-4659, and 0.59% for *Cz. merolae* MS1, revealing substantial differences in haplotype divergence among these diploid strains (Fig. 3b).

To assess whether geographic separation is associated with increased genetic divergence, we next compared sequence divergence between geographically distant strains of *Cc. yangmingshanensis* [NIES-4659 {Hakone, Japan} vs. 8.1.23 F7 {Kula, Turkey}] and *Cz. merolae* [MS1 {YNP, United States} vs. 10D {Sardinia, Italy}]. Mapping genomic reads from haploid strain 8.1.23 F7 to the haploid NIES-4659 Ha1 reference genome yielded an interstrain SNP/indel rate of 1.26%, which is lower than the haplotype divergence within NIES-4659 (2.19%). In contrast, mapping reads from haploid strain 10D to the haploid strain MS1 Ha3 reference genome resulted in an interstrain divergence of 0.71%, slightly higher than the haplotype divergence within MS1 (0.574%).

Thus, it remains unclear whether geographic separation contributes to genetic divergence in Cyanidiophyceae. However, the ability of haploid strains 10D and MS1 Ha3 to mate and form a hybrid diploid, together with the largely conserved chromosomal architecture despite the presence of inversions and translocations, suggests relatively high genome stability in cyanidiophycean genomes (excluding *Galdieria*), although further investigation of additional strains will be necessary to confirm this trend.

### Comparison of genome features with other eukaryotes

Based on the refined datasets, we compared the genomes of cyanidiophycean algae with previously sequenced genomes of other eukaryotes in terms of genome size and the number of protein-coding genes (Fig. 3c; Data Set S4). The genome of *Cd. caldarium* (8.73 Mb) is among the smallest of all sequenced eukaryotic genomes, excluding parasitic microsporidia (2.5 to 21.6 Mb) (Fig. 3c), and is comparable to that of the methylotrophic yeast *Ogataea polymorpha* (8.97 Mb) (Riley et al. 2016). Specifically, the number of protein-coding genes (4,884 to 7,832) in cyanidiophycean algae is also comparable to those of yeasts, such as *O. polymorpha* (5,177) (Riley et al. 2016), *Saccharomyces cerevisiae* (6,569) (Goffeau et al. 1996), and *Schizosaccharomyces pombe* (4,970) (Wood et al. 2002), although the nuclear genomes of cyanidiophycean algae additionally encode genes related to photosynthesis, as well as chloroplast biogenesis and regulation. Within Archaeplastida, several green algal lineages also possess streamlined nuclear genomes (12.9 to 17.4 Mb); however, the number of protein-coding genes is relatively large, ranging from ∼7,300 to over 9,300 (Foflonker et al. 2018; Lemieux et al. 2019; Kato et al. 2023) (Data Set S4). Comparative analyses of orthogroups within Archaeplastida further revealed that cyanidiophycean algae possess a remarkably streamlined gene repertoire compared with other photosynthetic eukaryotes (Fig. S4). In addition, their nuclear genomes encode a relatively small number of regulatory genes, such as TFs (80 to 169) and transcriptional regulators (TRs) (70 to 86) (Fig. 3d; Data Set S4).

Gene duplication is widely observed among eukaryotic genomes and is considered one of the major evolutionary mechanisms, providing the genetic material necessary for the emergence of genes with new or modified functions (Ohno 1970). In flowering plants (angiosperms), approximately 65% of genes have duplicated copies, most of which are derived from multiple ancient whole-genome duplication events (Panchy et al. 2016). In contrast, the genomes of cyanidiophycean algae (excluding *Galdieria*) have a high proportion of single-copy genes (∼80%; Fig. 3d). Taken together, Cyanidiophyceae possess one of the simplest genomic architectures among free-living eukaryotes examined to date, including photosynthetic lineages.

### Exclusive diploid dominance of Cyanidiophyceae in natural habitats

**Figure 4:**
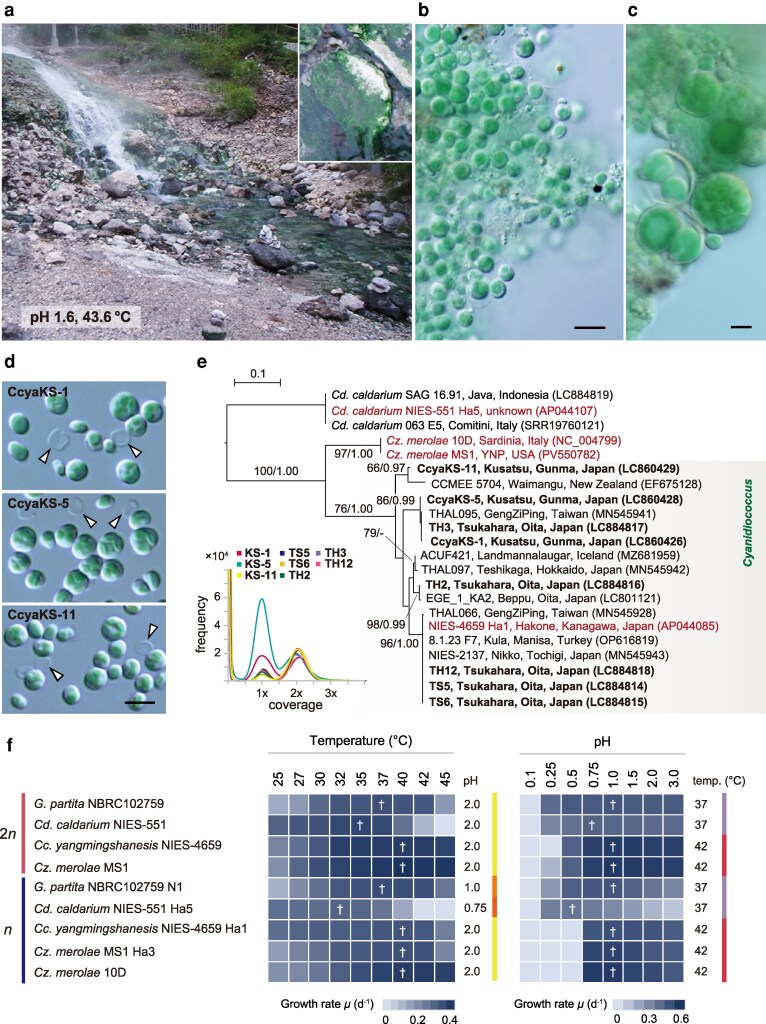
Natural habitat and optimal growth conditions of Cyanidiophyceae. (a) Cyanidiophycean algae inhabiting an acidic hot spring in Kusatsu were predominantly found in association with stones in the water. (b and c) DIC micrographs of morphologically diploid *Cyanidiococcus* (b) and *Galdieria* (c) obtained from blue–green mats collected in the acidic hot spring. Scale bar: 5 *µ*m. (d) DIC micrograph of cultured cell-walled *Cyanidiococcus* strains isolated from the acidic hot spring in Kusatsu. The arrowhead indicates the mother cell wall released upon hatching of daughter cells. Scale bar: 5 *µ*m. (e) Phylogenetic relationships of cyanidiophycean strains, including strains collected from Kusatsu and Tsukahara hot springs (highlighted in bold), based on nucleotide sequences of the chloroplast-encoded *rbcL* were inferred using ML analysis. *Cd. caldarium* was used as the outgroup. Bootstrap probabilities (BPs) > 50% (left) from ML analysis and Bayesian inference (BI) > 0.95 (right) from Bayesian analysis are shown above the branches. Branch lengths represent evolutionary distances, as indicated by the scale bar. Strains used in this study are highlighted in red. Histogram of *k*-mer counts (*k* = 41) generated from whole-genome Illumina short reads of *Cc. yangmingshanensis* strains collected from Kusatsu and Tsukahara hot springs, using Jellyfish ver. 2.3.0 (Marcais and Kingsford 2011). (f) Heatmaps showing the growth rate, based on increases in OD_750_, under the indicated temperature and pH conditions. Daggers (†) denote the highest growth rates for each alga. See also Data Set S5 for details.

Most strains of *Galdieria*, *Cyanidiococcus*, and *Cyanidioschyzon* isolated from acidic hot springs worldwide possess a cell wall (Toplin et al. 2008; Huang et al. 2024). This observation suggests that Cyanidiophyceae predominantly exist in the diploid phase in natural environments. To investigate whether haploid cells are also present in these habitats, we conducted field sampling at 2 sulfuric hot springs in Japan: Kusatsu Hot Spring (Fig. 4a; Fig. S5, a to d) and Tsukahara Hot Spring (Fig. S5e). Cyanidiophycean algae were often found attached to stones on the riverbed, forming blue–green mats (Fig. 4a; Fig. S5e). We collected these mats and examined them using microscopy, which revealed only cells with diploid morphology and no cells with haploid morphology (Fig. 4, b and c; Fig. S5f). Among them, smaller cells morphologically similar to diploid *Cyanidium*, *Cyanidiococcus*, or *Cyanidioschyzon* were dominant (Fig. 4b; Fig. S5f), although larger cells corresponding to *Galdieria* diploids were occasionally observed (Fig. 4c). To identify the taxonomic affiliation of the smaller cells, we isolated single cells and established clonal cultures. In all resulting cultures, remnants of the mother cell wall were observed following the hatching of daughter cells (Fig. 4d; Fig. S5g). Phylogenetic analysis of the *rbcL* gene classified all clones into the genus *Cyanidiococcus* (Fig. 4e). Furthermore, *k*-mer analysis of Illumina short reads revealed 2 peaks (Fig. 4e), indicating that all clones are heterozygous diploids.

Members of the Cyanidiophyceae are also known to inhabit the surfaces of humid mud and the endolithic regions (a layer a few millimeters beneath the rock surface) above the waterline (Gross et al. 1998; Qiu et al. 2013; Van Etten et al. 2023), observations of these habitats likewise yielded only cell-walled diploid forms (Fig. S5, a to d).

Taken together, these results suggest that Cyanidiophyceae exist exclusively in the diploid phase in their natural habitats and that haploid cells may arise only under specific and infrequent conditions, such as rare environmental stimuli or in locally distinct microhabitats.

### Comparison of growth rates among species and between haploid and diploid phases under various conditions

Several eukaryotic organisms have been identified in acidic environments (pH < 4.0), such as acid mine drainage (AMD) and geothermal hot springs (Amaral-Zettler 2012). The low pH in these environments facilitates the solubilization of metals into water. Consequently, acidophilic organisms generally possess mechanisms to tolerate not only low pH but also high concentrations of metals, including toxic heavy metals. Here, we estimated the optimal temperature and pH, as well as metal tolerance, based on the increase rate of optical density at 750 nm (OD_750_) under the respective conditions. Although the relationship between OD_750_ and cell density (ie the conversion factor) varies among culture conditions and strains, OD_750_ was measured within the linear range of its correlation with cell density for each strain under the respective conditions.

To compare temperature preferences among the 4 genera of Cyanidiophyceae and between their respective diploid and haploid phases, cells of both phases of each strain were cultured across a temperature gradient ranging from 25 to 45 °C (Fig. 4f; Data Set S5). Each culture was maintained at a specific pH: pH 1.0 for *Galdieria* haploid cells, pH 0.75 for *Cyanidium* haploid cells, and pH 2.0 for the remaining strains. In *G. partita*, diploid cells exhibited optimal growth at 30 to 42 °C (Fig. 4f; here and below, the conditions for which the growth rate did not differ significantly from the peak according to Tukey's multiple comparison test are defined as optimal) and haploids at 32 to 42 °C, with both showing the highest growth rate at 37 °C (Fig. 4f). *Cd. caldarium* showed optimal growth at 25 to 37 °C for diploids (peak at 35 °C) and haploids at 25 to 35 °C (peak at 32 °C) (Fig. 4f). *Cc. yangmingshanensis* diploid cells exhibited optimal growth at 32 to 45 °C and haploids at 27 to 42 °C, with both peaking at 40 °C (Fig. 4f). In *Cz. merolae,* diploid cells (strain MS1) exhibited optimal growth at 32 to 45 °C, while haploid strains MS1 Ha3 and 10D exhibited optimal growth at 37 to 45 °C and 32 to 42 °C, respectively, with maximum growth again at 40 °C for both phases (Fig. 4f).

To evaluate pH preferences, diploid and haploid cells of each strain were cultured across a pH gradient ranging from 0.1 to 3.0 (Fig. 4f; Data Set S5). The temperature was set at 37 °C for *Galdieria* and *Cyanidium* and at 42 °C for the other strains. In *G. partita*, diploid cells exhibited optimal growth across pH 0.5 to 3.0, peaking at pH 1.0, whereas haploid cells preferred pH 0.75 to 1.0 with peak growth at pH 1.0 (Fig. 4f). In *Cd. caldarium*, diploid cells exhibited optimal growth at pH 0.5 to 3.0 and peaked at pH 0.75, while haploid cells grew best at pH 0.25 to 1.5 with a peak at pH 0.5 (Fig. 4f). In *Cc. yangmingshanensis*, both diploid and haploid cells exhibited optimal growth across pH 0.75 to 3.0 and showed maximum growth at pH 1.0. In *Cz. merolae*, diploid cells (strain MS1) exhibited optimal growth from pH 0.5 to 3.0, while haploid strains (MS1 Ha3 and 10D) exhibited optimal growth from pH 0.75 to 2.0, with peak growth at pH 1.0 for both phases (Fig. 4f).

These results indicate that in all 4 genera, diploid cells exhibit a broader range of tolerance to both temperature and pH compared to haploid cells. However, the optimal conditions for growth are largely similar between the haploid and diploid phases. Notably, both the diploid and haploid phases of *Cd. caldarium* NIES-551 exhibited a lower preferred temperature range compared to the other genera. Whether this trait represents a synapomorphy of the genus *Cyanidium* remains to be determined, and further investigation of additional strains is required. However, since its sister genus, *Cyanidiofrigus pintoensis* (Cyanidiales in Fig. 1c), inhabits meso-acidophilic environments (Huang et al. 2024), it is plausible that *Cyanidium* has adapted to more moderate conditions during its evolutionary divergence from other thermo-acidophilic members of Cyanidiophyceae.

To compare metal tolerance, diploid and haploid cells of each strain were cultured in MA medium supplemented with different concentrations of arsenate (As(V)) and nickel (Ni) ranging from 0 to 50 mm (Fig. S5d; Data Set S5). Given that metal toxicity is highly dependent on pH (Peterson et al. 1984), all media used in this assay were adjusted to pH 0.75. As a result, both diploid and haploid cells of *G. partita* exhibited higher tolerance to As(V) than other cyanidiophycean algae (Fig. S5d), consistent with a previous study of *Galdieria yellowstonensis* (*G*. *yellowstonensis*) 108.79 E11 (Cho et al. 2023). This result is likely due to the presence of the *arsB* and *arsC* genes of HGT origin, which are encoded only in the *Galdieria* genome (Cho et al. 2023). Regarding Ni tolerance, both diploid and haploid cells of *Cd. caldarium* exhibited greater Ni tolerance than other cyanidiophycean algae (Fig. S5h). A recent study showed that *Cz. merolae* 10D avoids Ni accumulation inside the cells in the presence of a high concentration of Ni (Marchetto et al. 2024). Although the molecular mechanism of Ni tolerance is still unclear, it is suggested that each member of Cyanidiophyceae has evolved its genome to adapt to different environments. For tolerance to both As(V) and Ni, there was little difference between the haploid and diploid phases (Fig. S5h), unlike the larger ranges of temperature and pH tolerated by diploids compared with haploids (Fig. 4f), suggesting that the presence or absence of a cell wall does not affect heavy metal tolerance.

### Development of procedures for genetic manipulation

Among Cyanidiophyceae, genetic manipulation was first developed in *Cz. merolae* 10D, where linear DNA constructs are introduced into cell wall-less cells by PEG-mediated transformation via homologous recombination (Ohnuma et al. 2008). Recently, we adapted this method to haploid *Galdieria* cells, which also lack a cell wall (Hirooka et al. 2022).

**Figure 5:**
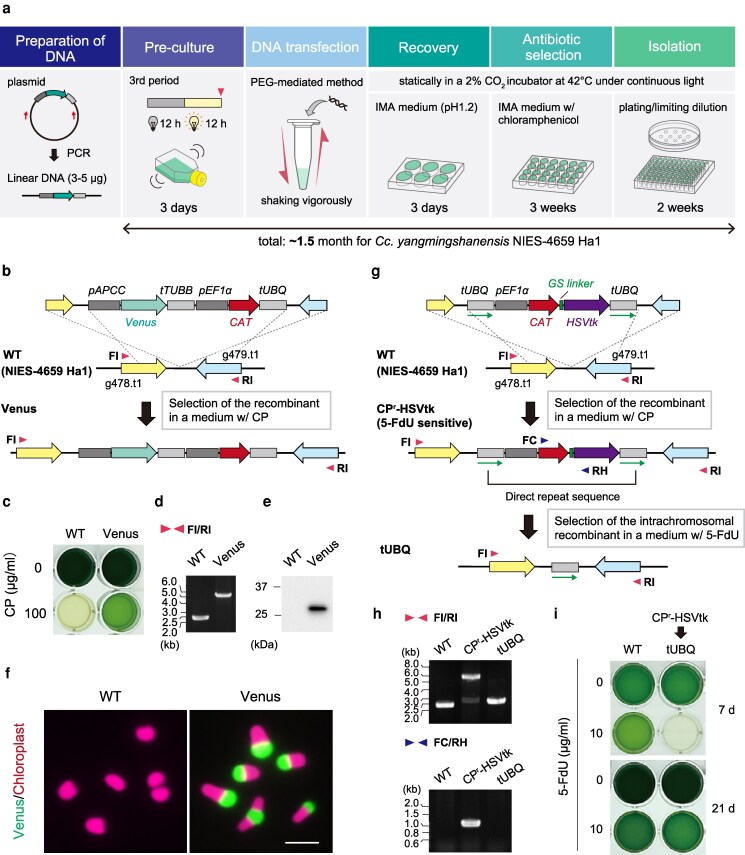
Genetic manipulation of *Cc. yangmingshanensis* NIES-4659 haploid clone Ha1. (a) Workflow of the transformation protocol used in *Cc. yangmingshanensis* to obtain transformants. (b) Schematic diagram showing insertion of the Venus expression cassette and the CAT selectable marker into a chromosomal intergenic region by homologous recombination in the *n* clone. (c) Transformed cells (Venus) were selected in IMA medium at pH 1.2 with chloramphenicol (CP) for 3 wk. (d) Targeted integration of the transgenes was confirmed by PCR using primers FI and RI indicated by arrowheads in (b). (e and f) Expression of Venus protein was confirmed by immunoblotting (e) and by fluorescence microscopy (f) (green, Venus fluorescence; magenta, chloroplast fluorescence). The wild type (WT) served as a negative control. Scale bar: 2 *µ*m. (g) Schematic diagram showing targeted integration and subsequent removal of the CAT selectable marker. The CAT selectable marker and the HSV*tk* suicide marker, linked by a GS linker, were flanked by 2 directly repeated *t*UBQ sequences (green arrows). This construct was integrated into the intergenic region by homologous recombination. After selection of the transformant (CP^r^-HSVtk) in the presence of CP, the selectable marker was excised through intrachromosomal homologous recombination between the 2 *t*UBQ copies, followed by selection with 5-FdU. (h) Confirmation of recombination events by PCR using the primers FI, RI, FC, and RH, indicated by arrowheads in (g). (i) The CP^r^-HSVtk cells were cultured for 21 d in the presence or absence of 5-FdU. The WT haploid clone served as a negative control.

To further facilitate comparative and functional genomic studies in Cyanidiophyceae, we subsequently extended this genetic modification procedure to the cell wall-less haploid cells of *Cc. yangmingshanensis* and *Cd. caldarium* (Fig. 5a). First, we constructed a plasmid encoding a *Venus* (a fluorescent protein) expression cassette and a chloramphenicol resistance gene (chloramphenicol acetyltransferase, *CAT*) as a selectable marker, flanked on both sides by sequences identical to a chromosomal intergenic region to allow targeted integration (Fig. 5b; Data Set S6). Linear DNA, amplified by PCR, was introduced into precultured cells of *Cc. yangmingshanensis* NIES-4659 Ha1 using a PEG-mediated transformation method developed for *Cz. merolae* 10D (Ohnuma et al. 2008; Fujiwara and Ohnuma 2018), with minor modifications. The cells were then recovered for 3 d in iron-rich MA (IMA) liquid medium at pH 1.2 under continuous light without any antibiotic and subsequently transferred to the same medium supplemented with chloramphenicol for selection. After incubation for 3 wk, chloramphenicol-resistant cells were observed (Fig. 5c). These cells were serially diluted into chloramphenicol-free IMA liquid medium or plating on cornstarch slurry spots over gellan gum–solidified IMA plates. After ∼2 wk of incubation, clonal colonies appeared. PCR analysis of genomic DNA from a chloramphenicol-resistant clone of *Cc. yangmingshanensis* confirmed correct integration of the *Venus* expression cassette and *CAT* marker into the intergenic region (Fig. 5d). Stable expression of *Venus* was validated by immunoblotting and fluorescence microscopy (Fig. 5, e and f).

A transformant of *Cd. caldarium* NIES-551 Ha5 was obtained using the same procedure, except that the IMA medium was adjusted to pH 0.75 and the culture was maintained at 37 °C. Additionally, because the *CAT* selectable marker was ineffective in this strain, it was replaced with a blasticidin S resistance cassette (blasticidin S deaminase, *BSD*) (Fig. S6a; Data Set S6), which had previously been shown to function in *Cz. merolae* 10D (Fujiwara et al. 2021) and *G. partita* NBRC 102759 haploid clones (Hirooka et al. 2022; Yamashita et al. 2025). As a result, a blasticidin S-resistant transformant expressing *Venus*, with integration of the *Venus* expression cassette and *BSD* marker into the intergenic region, was successfully obtained (Fig. S6, b to d).

Recently, we developed a procedure in *G. partita* to remove the selectable marker (*BSD*) from a transformant using a combination of the herpes simplex virus thymidine kinase (*HSVtk*) suicide marker, which converts ganciclovir into a toxic compound (Smith et al. 1982), and intrachromosomal recombination (Hirooka et al. 2022). Using this technique and reusing the *BSD* selectable marker, 2 or more chromosomal loci can be modified in a single strain (Hirooka et al. 2022). To expand this technique to other members of Cyanidiophyceae, we first generated a *Cc. yangmingshanensis* transformant expressing a *CAT–HSVtk* fusion protein, in which *HSVtk* is fused to the C-terminus of *CAT* via a glycine-serine linker (Fig. 5, g and h; Data Set S6). When the *CAT–HSVtk* transformant was cultured in MA medium supplemented with various concentrations of ganciclovir, its growth was slightly inhibited but not completely suppressed—contrary to expectations and unlike the case in *G. partita* (Fig. S6e). Since *HSVtk* also converts 5-fluoro-2′-deoxyuridine (5-FdU) into the highly toxic compound 5-fluoro-2′-deoxyuridylate (5-FdUMP), which inhibits thymidylate synthase and leads to cell death (Fig. S6f) (Yagil and Rosner 1971), we tested whether 5-FdU could inhibit the growth of *CAT–HSVtk*-expressing *Cc. yangmingshanensis* cells. When both wild-type and *CAT–HSVtk* transformant cells were cultured in media supplemented with different concentrations of 5-FdU (Figure S6e), most transformant cells died within 7 d, while wild-type cells survived at 10 *µ*g/mL 5-FdU (Fig. S6e). Upon continued cultivation, cells that had lost the *CAT–HSVtk* cassette via intrachromosomal homologous recombination between the direct repeat sequences (2 copies of the *UBQ* terminator) flanking the *CAT–HSVtk* cassette (Fig. S6f) appeared after 21 d (Fig. 5i). The expected recombination event was confirmed by PCR (Fig. 5h). Furthermore, we successfully applied this selectable marker deletion strategy to *Cz. merolae* 10D (Fig. S6, g to j; Data Set S6).

In summary, genetic manipulation at targeted chromosomal loci has now been developed for all axenically cultivable genera of Cyanidiophyceae—*Cyanidioschyzon*, *Cyanidiococcus*, *Cyanidium*, and *Galdieria*—and the method for selectable marker removal enables marker recycling for the editing of 2 or more loci.

### Diploid formation by isogamous mating of haploids and identification of the candidate MTR

In *G. partita*, haploid cells not only undergo isogamous mating between distinct mating types to form heterozygous diploids but also occasionally undergo self-diploidization by endoreduplication, resulting in homozygous diploids (Hirooka et al. 2022). Self-diploidization was also observed in *Cc yangmingshanensis* NIES-4659 Ha1, where cell-walled diploid cells occasionally emerged in the haploid clonal culture. By isolating and propagating the emerged diploid cell, a homozygous diploid culture derived from NIES-4659 Ha1 was obtained. However, this ability of self-diploidization disappeared after prolonged subculturing of haploids, unlike in *G. partita*. The resulting homozygous diploids, lacking interallelic polymorphisms, were suitable for direct comparison with haploids, as described below.

**Figure 6:**
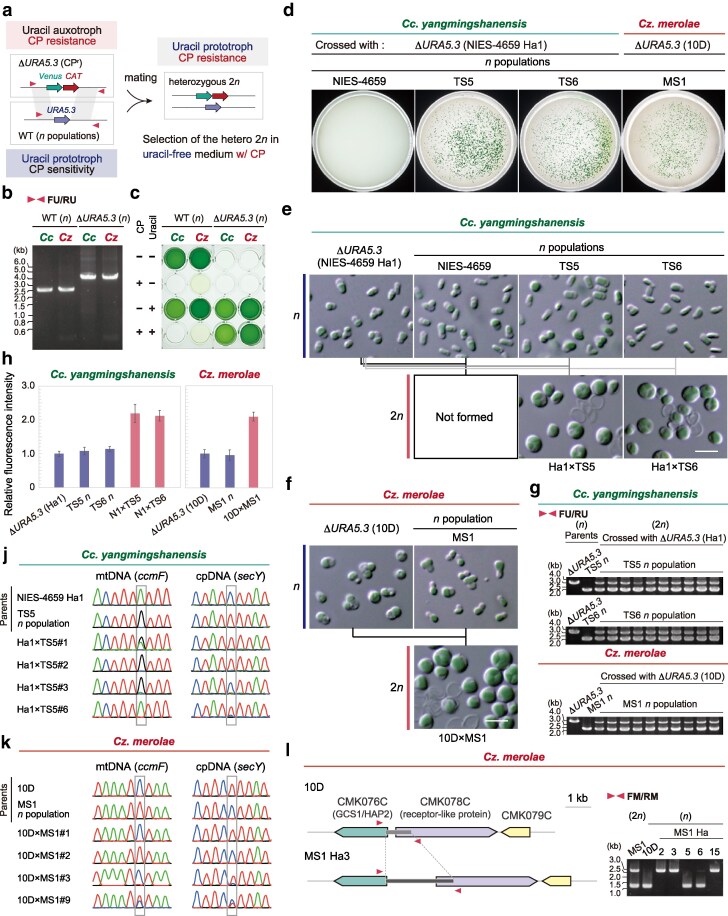
Diploid generation by haploid mating, candidate MTRs, and organellar DNA inheritance in *Cc. yangmingshanensis* and *Cz. merolae*. (a) Schematic illustration of the strategy for selecting heterozygous 2*n* cells possessing both the CAT selectable marker and the *URA5.3* gene, following mating of the Δ*URA5.3* (CP^r^) *n* clone with a WT *n* population derived from a 2*n* clone in uracil-free, CP-supplemented medium. (b) Replacement of the chromosomal *URA5.3* locus with the Venus expression cassette and the CAT selectable marker in the generated Δ*URA5.3 n* clone was confirmed by PCR using primers FU and RU (arrowheads in (a)). The WT *n* clone served as a control. Cc, *Cc. yangmingshanensis*; Cz, *Cz. merolae*. (c) Uracil auxotrophy and CP resistance of the Δ*URA5.3 n* clones were confirmed by cultivation for 7 d in the presence or absence of uracil and CP. WT *n* clones served as a control. Cc, *Cc. yangmingshanensis*; Cz, *Cz. merolae.* (d) Δ*URA5.3* (CP^r^) *n* clones of *Cc. yangmingshanensis* and *Cz. merolae* were crossed with their respective WT *n* populations. Heterozygous 2*n* cells generated by mating were selected on HATF Immobilon nitrocellulose membranes (85 mm) placed over gellan gum–solidified medium at pH 2.0 with CP. The phylogenetic relationships among strains NIES-4659, TS5, TS6, 10D, and MS1 are shown in Fig. 4e. (e) DIC micrographs of *Cc. yangmingsha*n*ensis*: Δ*URA5.3* (NIES-4659 Ha1) *n* clone, *n* populations derived from the original 2*n* clones NIES-4659, TS5, and TS6, and hybrid 2*n* clones Ha1 × TS5 and Ha1 × TS6. Scale bar: 5 *µ*m. (f) DIC micrographs of *Cz.* m*erolae*: Δ*URA5.3* (10D) *n* clone, *n* population derived from the original 2*n* clone MS1, and the hybrid 2*n* clone 10D × MS1. Scale bar: 5 *µ*m. (g) Heterozygosity of the hybrid 2*n* clones was confirmed by PCR using primers FU and RU. (h) Nuclear DNA content compared by measuring fluorescence intensity of DAPI-stained nuclei. The mean fluorescence intensity of Δ*URA5.3 n* cells was defined as 1.0. Data are means ± Sd from 20 independent cells. (i) Schematic diagram showing the candidate MTR in *Cz. merolae*. The region was amplified by PCR using primers FM and RM, which are indicated by arrowheads in the left panel. (j and k) Sanger sequencing chromatograms of mitochondrial (*ccmF*) and chloroplast (*secY*) DNA regions from parental *n* clones and their 2*n* progenies of *Cc. yangmingshanensis* (J; colonies #1, #2, #3, and #6) and *Cz. merolae* (K; colonies #1, #2, #3, and #9). Boxes indicate nucleotide variants between the parental *n* clones. Green, adenine; red, thymine; black, guanine; blue, cytosine.

To investigate whether diploids arise through mating between haploids in cyanidiophycean genera other than *Galdieria*, uracil-auxotrophic, drug-resistant haploid strains were generated. In *Cz. merolae* 10D, URA5.3 (UMP synthase) mutants exhibiting uracil auxotrophy have previously been generated and used as host strains for genetic manipulation (Minoda et al. 2004; Taki et al. 2015). In the same manner, we replaced the chromosomal *URA5.3* locus with *Venus–CAT* in *Cc. yangmingshanensis* NIES-4659 Ha1 and *Cz. merolae* 10D, or with *Venus–BSD* in *Cd. caldarium* NIES-551 Ha5 (Fig. 6, a and b; Fig. S7, a and b). These Δ*URA5.3* strains express *Venus* (Fig. S7c) and exhibit uracil auxotrophy along with resistance to chloramphenicol or blasticidin S, whereas the wild-type strains are uracil prototrophic and drug sensitive (Fig. 6c; Fig. S7b). Therefore, diploids resulting from mating between Δ*URA5.3* haploids and wild-type haploids would possess both the drug resistance gene (*CAT* or *BSD*) and *URA5.3* and are thus expected to exhibit both drug resistance and uracil prototrophy (Fig. 6a). A similar strategy was previously employed in *G. partita* mating experiments (Hirooka et al. 2022).

For mating, each Δ*URA5.3* haploid clone was cocultured with the corresponding wild-type haploid population—derived from heterozygous diploid strains—that should contain both mating types, if such sexuality exists, in MA medium supplemented with uracil, under a 12-h light/12-h dark cycle for 7 d (or 10 d for *Cd. caldarium*). After mating, the mixed cultures were transferred to selective conditions lacking uracil and containing the relevant drug. For *Cc. yangmingshanensis* and *Cz. merolae*, the cultures were plated on nitrocellulose membranes over gellan gum–solidified MA medium with chloramphenicol. For *Cd. caldarium*, the culture was incubated in liquid MA medium with blasticidin S.

As a result, no colonies appeared when Δ*URA5.3* (*Cc. yangmingshanensis* NIES-4659 Ha1) was crossed with the wild-type haploid population derived from the heterozygous diploid NIES-4659 (the reason remains unclear). However, crosses with other wild-type haploid populations (derived from heterozygous diploid TS5 or TS6; Fig. 4e; Fig. S4c) did yield colonies (Fig. 6d). Similarly, for *Cz. merolae*, crosses between Δ*URA5.3* (10D) and wild-type haploid population derived from the MS1 diploid also produced colonies (Fig. 6d). For *Cd. caldarium*, crosses between Δ*URA5.3* (NIES-551 Ha5) and wild-type haploid populations derived from the diploids NIES-551 and SAG 16.91 yielded growing cells in the liquid medium as well (Fig. S7d). The cells in these colonies or in the liquid culture showed diploid morphology (Fig. 6, e and f; Fig. S7e), genetic heterozygosity (Fig. 6g; Fig. S7f), approximately twice the nuclear DNA content of haploid cells (Fig. 6h; Fig. S7g), and expression of *Venus* (Fig. S7h). These results indicate that *Cyanidium*, *Cyanidiococcus*, and *Cyanidioschyzon* also undergo isogamous sexual reproduction and that *Cz. merolae* 10D is a haploid clone capable of sexual reproduction.

Notably, when Δ*URA5.3* haploid clones of *Cd. caldarium* and *Cz. merolae* were cocultivated with their corresponding wild-type haploid clones (*Cd. caldarium* NIES-551 Ha5 and *Cz. merolae* 10D), no drug-resistant and uracil-prototrophic colonies or cells were observed (Figs. S7d and S8a), suggesting that homothallic mating—ie mating-type switching in a subset of cells within a population of the same mating type (Vanwinkle-Swift and Hahn 1986)—does not occur in these species. The same was observed for *G. partita* (Hirooka et al. 2022). This experiment could not be conducted for *Cc. yangmingshanensis* because the Δ*URA5.3* clone of NIES-4659 Ha1 failed to generate diploids when crossed with the wild-type haploid population derived from the NIES-4659 diploid as above. In addition, when *Cz. merolae* 10D Δ*URA5.3* was cocultivated with various haploid clones of *Cz. merolae* MS1 (Ha2, Ha3, Ha5, Ha6, and Ha15), diploid colonies were produced in crosses with Ha2, Ha3, and Ha15 (ie drug-resistant and uracil-prototrophic), but not with Ha5 or Ha6 (Fig. S8, a to c). These results suggest the presence of 2 mating types: 10D, Ha5 and Ha6 versus Ha2, Ha3, and Ha15.

MTRs in a chromosome, which contribute to mating-type differences and are typically associated with recombination suppression, have evolved independently in eukaryotes (Beukeboom and Perrin 2014). To identify the candidate MTR in *Cz. merolae*, we compared the genomes of 2 strains with different mating types, 10D and MS1 Ha3. The comparison revealed a compact region with sequence differences between the 2 mating types, indicating recombination suppression, located between CMK076C and CMK078C (10D, ∼1 kb; MS1 Ha3, ∼2.6 kb) (Fig. 6i). In the diploid strain *Cz. merolae* MS1, both candidate MTR variants were present in the genome, as confirmed by PCR analysis (Fig. 6i). Furthermore, the presence of either sequence type correlated with the mating type (Fig. 6i), indicating that this region represents the MTR. CMK076C encodes GENERATIVE CELL SPECIFIC 1/HAPLESS 2 (GCS1/HAP2), a membrane protein of the male gamete that is essential for gamete fusion and is conserved across a wide range of eukaryotic lineages (Mori et al. 2006; von Besser et al. 2006). CMK078C encodes a receptor-like protein containing leucine-rich repeat (LRR), growth factor receptor (GFR), and tumor necrosis factor receptor (TNFR) domains. Sequence differences within the MTR are expected to cause divergence in cis-regulatory sequences located immediately upstream of the ORFs, as well as in the N-terminal amino acid sequences of the encoded proteins between the 2 mating types (Fig. S9). Overall, these differences are likely to give rise to mating-type–specific gene regulation and function.

### Inheritance of organelle DNA during sexual reproduction

Unlike nuclear DNA, organellar DNA—mitochondrial (mtDNA) and chloroplast (cpDNA)—exists in multiple copies within a single organelle and cell and is uniparentally inherited in the majority of eukaryotes, although biparental inheritance has been reported in several lineages (Birky 1995). In Viridiplantae, approximately 20% of angiosperms exhibit biparental inheritance of cpDNA (Corriveau and Coleman 1988; Zhang et al. 2003). Furthermore, a population genetic study provided evidence for potential biparental inheritance accompanied by recombination in the green alga *Ostreococcus tauri* (Blanc-Mathieu et al. 2013). In red algae, uniparental inheritance of cpDNA has been observed in the multicellular species *Pyropia yezoensis* (formerly *Porphyra yezoensis*) (Choi et al. 2008), whereas patterns of organellar DNA inheritance in unicellular red algae remain unclear.

To clarify how organellar DNA is inherited in cyanidiophycean red algae, we performed crosses using haploid strain combinations that exhibit polymorphisms in both mtDNA and cpDNA: *Cc. yangmingshanensis* NIES-4659 Ha1 (Δ*URA5.3*) and TS5, *Cz. merolae* 10D (Δ*URA5.3*) and MS1, and *G. partita* NBRC 102759 N1 (Δ*URA1*; uracil auxotrophic and blasticidin S-resistant) (Hirooka et al. 2022) and *G. partita* NJ-5 (Fig. S8d). We then analyzed the sequences of mtDNA and cpDNA regions containing nucleotide variations between the parental strains in multiple diploid progeny colonies derived from these crosses (Fig. 6, j and k; Fig. S10). For *Cd. caldarium*, we were unable to prepare 2 strains with polymorphisms in both mtDNA and cpDNA.

As a result, except for mtDNA in *G. partita*, the majority of diploid colonies exhibited mtDNA and cpDNA sequences derived from only 1 of the parental strains, selected in an apparently random manner. In some cases, both mtDNA and cpDNA originated from the same parent, while in others, they were derived from different parents, indicating that mtDNA and cpDNA are inherited independently. In contrast, some diploid colonies retained mtDNA (eg colonies #1, #4, #7, #8, #9, and #10 of *Cc. yangmingshanensis* Ha1 × TS5 and colony #9 of *Cz. merolae* 10D × MS1) and cpDNA (eg colonies #7 and #8 of *Cc. yangmingshanensis* Ha1 × TS5, colony #9 of *Cz. merolae* 10D × MS1, and colonies #2, #4, #5, #7, #8, #9, and #10 of *G. partita* NBRC 102759 N1 × NJ-5) from both parents (Fig. 6, j and k; Fig. S10). In the yeast *S. cerevisiae*, it is known that mtDNA is biparentally inherited, but 1 parental type is rapidly lost, resulting in homoplasmy (Westermann 2014). Based on this, we investigated whether only 1 parental organellar genome is eventually retained in each cell of diploid colony #7 of *Cc. yangmingshanensis* Ha1 × TS5, colony #9 of *Cz. merolae* 10D × MS1, and colony #2 of *G. partita* NBRC 102759 N1 × NJ-5, all of which initially inherited mtDNA and/or cpDNA from both parents (Fig. S10). To test this, the diploid progenies were subcultured for 1 to 3 mo, after which the cells were spread onto gellan gum–solidified medium. Single colonies were isolated, and their mtDNA and cpDNA sequences were analyzed. As a result, all clones retained either mtDNA or cpDNA from only 1 parent, and the parental origin varied randomly among the colonies (Fig. S10). In addition, in the above investigations, we analyzed the behavior of 2 loci located at the most distant positions on the circular genome of each organelle: *ccmF* and *nad6* for mtDNA and *secY* and *psbD* for cpDNA. In all cases, the paired loci showed identical inheritance patterns (Fig. S10), indicating that no recombination occurred between organellar genomes from the 2 parents.

These results suggest that, except for mtDNA in *G. partita*, organellar DNA in Cyanidiophyceae is initially inherited biparentally when a diploid is formed by mating between haploids. However, during diploid proliferation, organellar genomes derived from 1 parent (randomly determined) are lost, resulting in homoplasmy. Notably, in *Cz. merolae* 10D, it has been reported that a single cell—containing a single mitochondrion and a single chloroplast—harbors approximately 80 copies each of mtDNA and cpDNA (Kuroiwa et al. 2021). Given such high copy numbers, random segregation is unlikely to account for the rapid resolution of heteroplasmy to homoplasmy, suggesting that a specific mechanism operates to ensure uniparental inheritance of organelle genomes. In contrast, mtDNA in *G. partita* diploids was consistently inherited from a specific parent (in our experiments, from *G. partita* NBRC 102759 N1) (Fig. S10a). This suggests that in Cyanidiophyceae, only *Galdieria* possesses a mechanism—similar to that found in most other eukaryotes—that ensures the uniparental inheritance of mtDNA from a specific mating type.

### Differences in transcriptome and histone modification between the diploid and haploid phases

**Figure 7:**
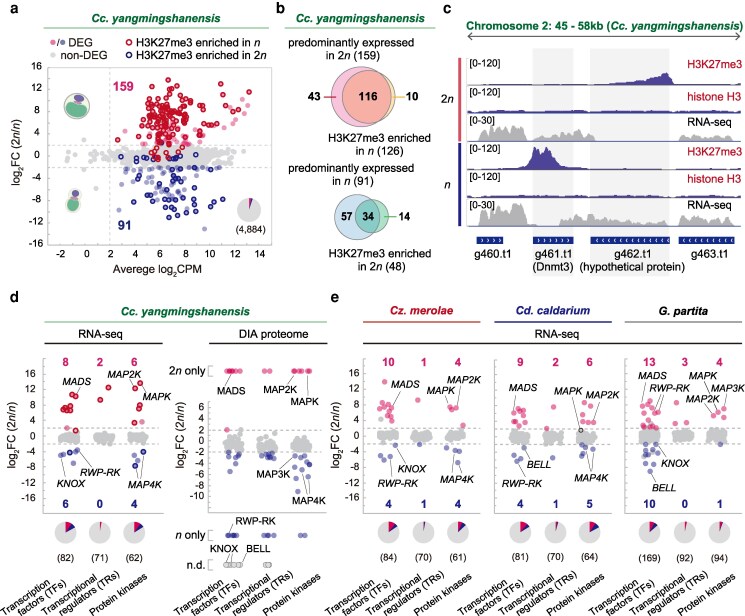
Comparison of transcriptomes and genomic distribution of H3K27me3 between diploid and haploid phases in Cyanidiophyceae. (a) MA plot showing 159 genes upregulated in 2*n* (homozygous 2*n* derived from NIES-4659 Ha1) and 91 genes upregulated in *n* (NIES-4659 Ha1) among 4,884 nucleus-encoded genes (DEGs; FDR < 0.01, log_2_CPM > 2, and |log_2_FC| > 2 in 3 independent cultures). (b) Venn diagrams showing the overlap between genes predominantly expressed in 2*n* cells and genes with elevated H3K27me3 levels in *n* cells (upper) and between genes predominantly expressed in *n* cells and genes with elevated H3K27me3 levels in 2*n* cells (lower). (c) Patterns of read count levels from ChIP-seq of H3K27me3 and histone H3, and RNA-seq, in chromosome 2: 45 to 58 kb in 2*n* and *n* cells. Normalized coverage (per million mapped reads) is shown. Numbers in brackets indicate value ranges. Gene models are shown as bars at the bottom. (d) Ratios of mRNA abundance and the abundance of the corresponding proteins between 2*n* and *n* cells for TF, TR, and protein kinase genes in *Cc. yangmingshanensis*. For the RNA-seq results, numbers in parentheses below the pie chart indicate the number of genes in each category. For the proteome results, proteins detected only in 2*n* (2*n* only), only in *n* (*n* only), or not detected in either (n.d.) are indicated above or below the graphs, respectively. See also Data Set S7. (e) Ratios of mRNA between 2*n* and *n* cells for genes encoding TFs, TRs, and protein kinases in *Cz. merolae*, *Cd. caldarium*, and *G. partita*. See also Data Sets S8 to S10.

To gain insights into the genetic basis of the phenotypic differences between diploid and haploid cells in Cyanidiophyceae, we compared the transcriptomes of haploid *Cc. yangmingshanensis* NIES-4659 Ha1 and its derived homozygous diploid cells (Fig. 7a; Data Set S7). In addition, we conducted a comparative proteome analysis to complement the transcriptome data (Data Set S7). For these omics comparisons, both haploid and diploid phases of *Cc. yangmingshanensis* were cultured under identical conditions within the optimal growth range (pH 2.0 and 40 °C). The results showed that among the 4,884 nucleus-encoded genes, 159 genes (3.3%) were predominantly expressed in the diploid (false discovery rate [FDR] < 0.01, log_2_CPM > 2, log_2_FC(2*n/n*) > 2), with 139 of these supported by the proteome analysis, while 91 genes (2.8%) were predominantly expressed in the haploid (FDR < 0.01, log_2_CPM > 2, log_2_FC(2*n/n*) < −2), with 74 supported by proteome analysis (Data Set S7). We also prepared comparative transcriptome datasets between diploid and haploid phases for *Cz. merolae*, *Cd. caldarium*, and *G. partita*, with both phases cultured under identical conditions within the optimal growth range for each species (pH 2.0, 40 °C for *Cz. merolae*; pH 0.75, 37 °C for *Cd. caldarium*; and pH 1.0, 37 °C for *G. partita*) (Fig. S11; Data Sets S8 to S10). Among them, similar expression patterns to those observed in *Cc. yangmingshanensis* were found in *Cd. caldarium* and *Cz. merolae*, which share 93.1% and 99.4% of their genes, respectively, with *Cc. yangmingshanensis* (Fig. 3a; Data Set S3), as described below.

In multicellular organisms, the histone modification H3K27me3 silences the expression of specific sets of genes, leading to phenotypic outcomes, such as cellular differentiation during development (Margueron and Reinberg 2011). Recent studies have shown that H3K27me3 also occurs in some unicellular eukaryotes. In the diatom *Phaeodactylum tricornutum* (*P. tricornutum*), for example, genes involved in the establishment and maintenance of cell morphology are marked with H3K27me3, and changes in this modification are associated with morphological changes (Zhao et al. 2021). In *Cz. merolae* 10D (which turned out to be a haploid clone, as described above), Gene Ontology (GO) enrichment analysis revealed that H3K27me3-targeted genes are significantly enriched for terms related to development (Mikulski et al. 2017).

Based on these findings, to examine whether H3K27me3 is involved in phenotypic changes between the haploid and diploid phases in Cyanidiophyceae, we compared the genome-wide distribution of H3K27me3 between haploid *Cc. yangmingshanensis* NIES-4659 Ha1 and its derived homozygous diploid using chromatin immunoprecipitation sequencing (ChIP-seq) (Fig. 7a; Data Set S7). The results showed that 126 genes are marked with H3K27me3 in haploid cells, 116 of which are predominantly expressed in diploid cells. In contrast, 48 genes are marked with H3K27me3 in diploid cells, 34 of which are predominantly expressed in haploid cells (Fig. 7, b and c). Thus, in *Cc. yangmingshanensis*, the patterns of genes marked with H3K27me3 in haploid and diploid phases largely correlate with those whose expression is suppressed in the corresponding phases, suggesting that phenotypic changes during the life cycle are epigenetically regulated.

### TFs and signaling pathways differentially expressed in haploid and diploid cells

The MAPK cascades regulate a wide variety of stimulus-responsive cellular processes, including proliferation, differentiation, and stress responses in eukaryotes (Plotnikov et al. 2011). The signals transmitted through these cascades enter the nucleus, where they modulate the activity of TFs and chromatin remodeling proteins (Plotnikov et al. 2011). In each of the nuclear genomes of *Cc. yangmingshanensis*, *Cz. merolae*, and *Cd. caldarium*, 3 MAPKs, 1 MAPK kinase (MAP2K), 1 MAPK kinase kinase (MAP3K), and 4 MAPK kinase kinase kinases (MAP4Ks) were identified by iTAK (Data Sets S7 to S9). Among them, 1 MAPK and 1 MAP2K were predominantly expressed in diploid cells, whereas 2 MAP4Ks were predominantly expressed in haploid cells in *Cc. yangmingshanensis*, *Cz. merolae*, and *Cd. caldarium* (Fig. 7, d and e). The only minor exception was that, in *Cd. caldarium*, 1 *MAPK* gene showed slightly higher expression in haploid cells (log_2_FC(2*n*/*n*) = 1.76, below the threshold for differential expression defined in this study) (Fig. 7e). In *Cc. yangmingshanensis*, proteome analyses also showed similar expression patterns of MAPK cascade components (Fig. 7d). In *Galdieria*, 3 MAPKs, 2 MAP2Ks, 1 MAP3K, and 5 MAP4Ks were identified, of which 1 MAPK, 1 MAP2 K, and 1 MAP3K were predominantly expressed in diploid cells (Fig. 7e). Thus, changes in MAPK cascades are likely involved in regulation of life cycle in Cyanidiophyceae.

Of the 82 TFs encoded in the nuclear genome of *Cc. yangmingshanensis* (84 in *Cz. merolae*, 80 in *Cd. Caldarium*, and 169 in *G. partita*), 8 genes—including 1 encoding a MEF2-type MADS-box TF—were predominantly expressed in diploid cells, while 6 genes (including those encoding KNOX and RWP-RK TFs) were predominantly expressed in haploid cells (Fig. 7d; Data Set S7). A similar expression pattern of TF genes was also observed in *Cd. caldarium*, *Cz. merolae*, and *G. partita* (Fig. 7e; Data Sets S8 to S10). Among these TF genes, 7 of the 8 diploid-dominant TFs were marked with H3K27me3 in haploid cells, whereas only 1 of the 6 haploid-dominant TFs was marked with H3K27me3 in diploid cells in *Cc. yangmingshanensis* (Fig. 7d; Data Set S7). Notably, the gene encoding DNA (cytosine-5)-methyltransferase 3 (Dnmt3)—the only predicted enzyme responsible for DNA methylation, which is associated with gene silencing in other eukaryotes (Lyko 2018)—was predominantly expressed in diploid cells in *Cc. yangmingshanensis*, *Cz. merolae*, and *Cd. caldarium* (Fig. 7c; Data Sets S7 to S9). These observations raise the possibility that distinct gene-silencing mechanisms are involved in haploid-to-diploid and diploid-to-haploid transitions. In contrast, the *Galdieria* genome encodes 3 *Dnmt3* genes, which are expressed at similar levels in both diploid and haploid cells (Data Set S10), suggesting that the gene-silencing mechanism associated with DNA methylation may differ from that in other cyanidiophycean algae.

The MADS-box family of eukaryotic TFs is involved in various cellular processes, including cell differentiation, cell wall reinforcement, and arginine metabolism in yeast and animals (Messenguy and Dubois 2003). In angiosperms, the MIKC-type MADS-box family has undergone substantial expansion and regulates diverse developmental processes, including flower formation (Smaczniak et al. 2012). MIKC-type MADS-box genes are conserved in streptophyte algae and land plants (together classified as Streptophyta in Fig. 1a) and are thought to have evolved from ancestral MEF2-type MADS-box genes—found in both green and red algae—through the acquisition of additional domains, such as the I and K domains (Thangavel and Nayar 2018). However, little is known about the function of MEF2-type MADS-box genes in unicellular green and red algae, although a previous study suggested that in the unicellular green alga *Coccomyxa subellipsoidea*, the gene is involved in development and stress tolerance (Nayar and Thangavel 2021). We previously found that in *Galdieria*, the single MEF2-type MADS-box gene encoded in the genome is predominantly expressed in the diploid phase, and its deletion inhibits the haploid-to-diploid transition, suggesting that it plays a role in diploid cell formation (Fig. 7e) (Hirooka et al. 2022). Similarly, in *Cc. yangmingshanensis*, *Cz. merolae*, and *Cd. caldarium*, the single MEF2-type MADS-box gene encoded in each genome was also predominantly expressed in diploid cells (Fig. 7, d and e). In *Cc. yangmingshanensi*s, proteome analyses also showed the exclusive expression of MADS-box protein in the diploid phase (Fig. 7d). These results suggest a conserved function of this gene across Cyanidiophyceae. The function of such MEF2-type MADS-box genes in diploid formation may reflect their origin in Archaeplastida, although future analyses, such as the identification of their target genes, could provide further insights.

KNOX and BELL-related proteins, both of which are TALE homeodomain (HD) TFs, regulate sexual life cycle transitions in some lineages of Viridiplantae (Lee et al. 2008; Sakakibara et al. 2013; Horst et al. 2016; Dierschke et al. 2021; Hisanaga et al. 2021). Similarly, the TALE-HD TFs OUROBOROS and SAMSARA promote the transition from the gametophyte (haploid) to the sporophyte (diploid) generation after fertilization in the multicellular brown alga *Ectocarpus* (Coelho et al. 2011; Arun et al. 2019). In the unicellular green alga *C. reinhardtii*, the KNOX protein GSM1 and the BELL-related protein GSP1 and the are exclusively expressed in minus and plus mating-type gametes, respectively (Lee et al. 2008). These proteins heterodimerize, trigger nuclear and organellar fusion between mating types (Kariyawasam et al. 2019), and activate diploid gene expression after mating (Lee et al. 2008). Furthermore, in multicellular red algae, the expression level of the *KNOX* gene varies with life cycle transitions in *Pyropia yezoensis* (Mikami et al. 2019), and a distinct pair of TALE-HD TFs is encoded on the sex-determining regions (SDRs) of the female and male, respectively, in *Bostrychia moritziana* (Petroll et al. 2025a). In *Cc. yangmingshanensis*, *Cz. merolae*, *Cd. Caldarium*, and *G. partita*, 2 *KNOX* genes and a single *BELL*-related gene are encoded in their nuclear genomes (Joo et al. 2018) (Data Sets S7 to S10). We previously showed that, in *Galdieria*, 1 of the 2 *KNOX* genes and the single *BELL*-related gene were predominantly expressed in haploids of both mating types, and deletion of either TF inhibited the haploid-to-diploid transition (Hirooka et al. 2022). In contrast, in *Cc. yangmingshanensis*, the 1 *KNOX* gene—but not the *BELL*-related gene—was predominantly expressed in haploid cells (Fig. 7d). However, proteome analysis failed to detect BELL-related proteins, and moreover, KNOX proteins were also not detected (Fig. 7d; Data Set S7). We also found that, in *Cz. merolae*, the *KNOX* gene was predominantly expressed in haploid cells of both mating types (10D and MS1 Ha3), while the *BELL*-related gene was not (Fig. 7e; Data Set S8). Conversely, in *Cd. caldarium*, the *BELL*-related—but not the *KNOX* gene—was predominantly expressed in haploid cells (Data Set S9). Considering these results, the haploid cultures of these 3 species used in this study may represent populations of vegetative haploid cells. Upon some form of stimulation, they may transform into as-yet-unknown gamete forms in which BELL-related and KNOX proteins are expressed as a result of a substantial increase in the mRNA levels of these genes, compared to those observed in this study.

RWP-RK TFs, found only in Archaeplastida, are involved in responses to nitrogen availability and sexual reproduction-associated processes (eg gamete sex determination, gametogenesis, and gametophyte development) in Viridiplantae (Chardin et al. 2014). Only a single RWP-RK TF gene is encoded in each genome of *Cc. yangmingshanensis*, *Cz. merolae*, and *Cd. caldarium*, and it is predominantly expressed in haploid cells (Fig. 7, d and e). In *Cc. yangmingshanensi*s, proteome analyses also showed the exclusive expression of RWP-RK protein in the haploid phase (Fig. 7d). In contrast, in *G. partita*, among the 11 RWP-RK TF genes encoded in the nuclear genome, 2 were predominantly expressed in diploid cells (Fig. 7e; Data Set S10). In *P. yezoensis*, it was reported that RWP-RK TF genes were upregulated both in gametophytes treated with the ethylene precursor 1-aminocyclopropane-1-carboxylic acid (ACC)—which promotes gamete formation—and in mature gametophytes (Uji et al. 2016). Although the precise roles of RWP-RK TFs in red algae remain to be elucidated, these observations suggest their possible involvement in sexual reproduction-associated processes, highlighting their deep evolutionary origin within Archaeplastida.

### Differentially expressed genes underlying differences in cellular architecture between haploid and diploid cells

Cell walls in photosynthetic eukaryotes display a considerable degree of diversity in their compositions and molecular architectures. Red algae typically possess composite cell walls that are primarily composed of cellulose microfibrils and also contain unique polysaccharides called sulfated galactans, including the economically important carrageenan and agar (Borg et al. 2023). However, Cyanidiophyceae have lost genes encoding cellulose synthase (CesA) and cellulose synthase-like (Csl) enzymes (Yin et al. 2009) and are thus thought to possess a unique cell wall. Notably, their ability to survive several months of darkness suggests that the cell wall is likely impermeable to protons, possibly as an adaptation to highly acidic environments (Gross 2000). In addition, previous study showed that in *Cd. caldarium* (the strain number was not reported, and thus its phylogenetic position remains unclear; it could potentially be assigned to *Cc. yangmingshanensis* or *Cz. merolae*), the cell wall is largely proteinaceous (50% to 55%), unlike other red algae (Bailey and Staehelin 1968).

**Figure 8:**
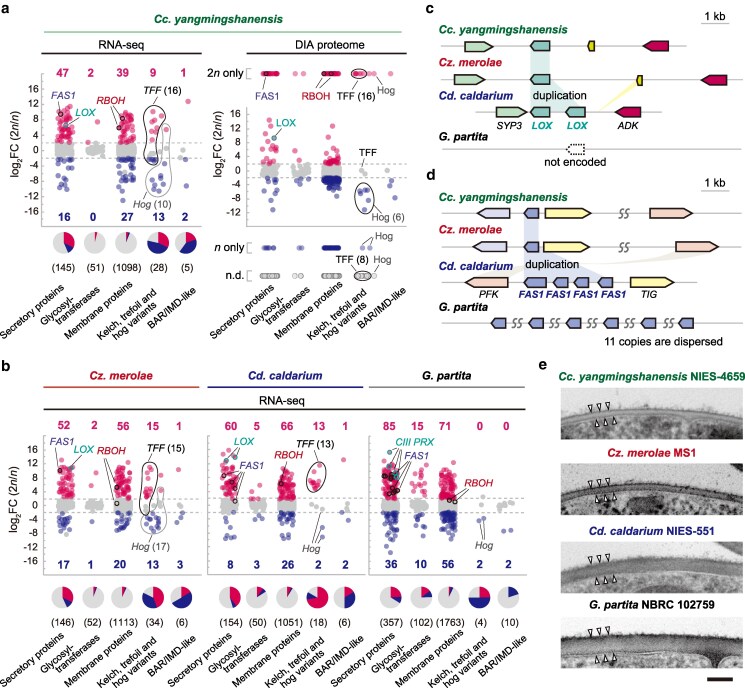
Comparison of transcriptomes and copy numbers of cell wall-related genes in Cyanidiophyceae. (a) Ratios of mRNA abundance and the abundance of the corresponding proteins between 2*n* and *n* cells for secretory proteins, glycosyltransferases, and other functional categories related to cell wall formation/regulation in *Cc. yangmingshanensis*. For the RNA-seq results, numbers in parentheses below the pie chart indicate the number of genes in each category. For the proteome results, proteins detected only in 2*n* (2*n* only), only in *n* (*n* only), or not detected in either (n.d.) are indicated above or below the graphs, respectively. See also Data Set S7 for details. (b) Ratios of mRNA abundance between 2*n* and *n* cells for genes encoding secretory proteins, glycosyltransferases, and other functional categories related to cell wall formation/regulation in *Cz. merolae*, *Cd. caldarium*, and *G. partita*. See also Data Sets S8–S10 for details. (c and d) Variation in the copy numbers of the cell wall–related genes *LOX* (c) and *FAS1* (d) in the nuclear genomes of cyanidiophyceaen algae. (e) Transmission electron micrographs of the cell walls of diploid cells in cyanidiophycean algae. Scale bar: 100 *µ*m.

In algae and plants, genes encoding secretory proteins and glycosyltransferases contribute to the formation of the extracellular matrix and cell wall. In *Cc. yangmingshanensis*, *Cz. merolae*, *Cd. caldarium*, and *G. partita*, approximately 35% to 50% of the genes encoding secretory proteins are differentially expressed between cell-walled diploid and cell wall-less haploid cells (Fig. 8, a and b). We found that, in *Cc. yangmingshanensis*, *Cz. merolae*, and *Cd. caldarium*, genes encoding secretory proteins, such as fasciclin (FAS1) domain proteins and lysyl oxidase (LOX), were predominantly expressed in diploid cells (Fig. 8, a and b). In *Cc. yangmingshanensi*s, proteome analyses also showed the predominant expression of these proteins in the diploid phase (Fig. 8a). FAS1 domain proteins are membrane-anchored glycoproteins involved in building cell wall structures in plants (Silva et al. 2020), and in *G. partita*, they are also expressed exclusively in diploid cells (Fig. 8b) (Hirooka et al. 2022). LOX plays a critical role in the formation and repair of the extracellular matrix by oxidizing lysine residues in fibrous proteins, such as elastin and collagen in animals (Kagan and Li, 2003), and it has been identified as an extracellular protein in *Cc. yangmingshanensis* RK-1 (renamed from *Cd. caldarium* RK-1 by Park et al. (2023)) (Tomo et al. 2018). LOX domain-containing genes are widely distributed in animals and other eukaryotes, as well as bacteria and archaea (Grau-Bove et al. 2015). However, within Archaeplastida, they have been identified only in *Porphyra* and in cyanidiophycean algae (except for *Galdieria*), suggesting the acquisition of these genes through HGT from other organisms.

We also found that the nuclear genomes of *Cd. caldarium* NIES-551 Ha5 and the previously sequenced *Cd. caldarium* 063 E5 (Cho et al. 2023) encode 2 copies of *LOX* and 4 copies of *FAS1* genes, all of which are arranged in tandem repeats, in contrast to the genomes of *Cz. merolae* 10D (Matsuzaki et al. 2004) and *Cc. yangmingshanensis* 8.1.23 F7 (Cho et al. 2023) and NIES-4659 Ha1, which each possess a single copy of these genes (Fig. 8, c and d). In *G. partita*, 11 copies of *FAS1* genes are dispersed throughout the genome (Fig. 8d). These differences, particularly in the copy number of *FAS1* genes, might be related to the variation in cell wall thickness observed among *Cc. yangmingshanensis* NIES-4659, *Cz. merolae* MS1, *Cd. caldarium* NIES-551, and *G. partita* NBRC 102759 (Fig. 8e).

Regarding glycosyltransferase genes, in *Cc. yangmingshanensis*, only 2 were predominantly expressed in diploid cells (2 in *Cz. merolae* and 5 in *Cd. caldarium*), whereas in *G. partita*, 13 were predominantly expressed in diploid cells (Fig. 8, a and b). Thus, *Cyanidiococcus* (as well as *Cyanidioschyzon* and *Cyanidium*) likely have a simpler cell wall polysaccharide composition than *Galdieria*.

In plants, reactive oxygen species (ROS), such as superoxide (O_2_^−^) and hydrogen peroxide (H_2_O_2_), play important regulatory roles in cell differentiation and development (Mhamdi and Van Breusegem 2018). Some enzymes, such as respiratory burst oxidase homologs (RBOHs) and Class III peroxidases (CIII PRXs), play predominant roles in apoplastic ROS regulation, contributing to cell wall loosening and hardening in plants (Cosio and Dunand 2009). The genome of *Cc. yangmingshanensis* encodes 2 *RBOH* genes (2 in *Cz. merolae* and 1 in *Cd. caldarium*), both of which are predominantly expressed in diploid cells of *Cc. yangmingshanensis* (Fig. 8a), a result supported by proteome analyses (Fig. 8a). In *Cz. merolae*, 1 of the 2 *RBOH* genes, and in *Cd. caldarium*, the single *RBOH* gene is predominantly expressed in diploid cells (Fig. 8b). In *P. yezoensis*, RBOH is suggested to be involved in archeospore (asexual reproductive spore) formation, which is associated with changes in cell wall composition (Gui et al. 2022). Although 2 *RBOH* genes are encoded in the *G. partita* genome, they are expressed at similar levels in both diploid and haploid cells (Data Set S10). In contrast, the *G. partita* genome encodes 4 *CIII PRX* genes, all of which are predominantly expressed in diploid cells (Fig. 8b) (Hirooka et al. 2022). No *CIII PRX* genes are found in the genomes of *Cyanidiococcus*, *Cyanidioschyzon*, or *Cyanidium*, nor in other red algae. The presence of *RBOHs* and *CIII PRXs*—enzymes typically found in plants—in Cyanidiophyceae (despite lineage-specific losses), and their expression specifically in cell-walled diploid cells, highlight the deep evolutionary origin of apoplastic ROS regulation by these proteins within Archaeplastida.

The majority of the 18 to 34 genes encoding proteins with combinations of Hedgehog (Hh), trefoil factor (TFF), and kelch (K)-like domains (eg TFF, Hog, K + TFF, and K + Hog) are encoded in the subtelomeric regions of several chromosomes in *Cd. caldarium*, *Cc. yangmingshanensis*, and *Cz. merolae* (Nozaki et al. 2007; Cho et al. 2023). In the *G. partita* genome, 2 K-like domain–containing genes and 2 Hh-like domain–containing genes are encoded, with the *Hh*-like genes located in subtelomeric regions, whereas *TFF*-like genes are absent. However, the proteins previously annotated as Hh in Cyanidiophyceae retain the carboxy-terminal autocatalytic domain HhC, which contains a Hint module, but lack the amino-terminal signaling domain HhN typically found in bona fide Hh proteins. Thus, these proteins encoded in cyanidiophycean genomes have been reclassified as non-Hh Hog proteins (Burglin 2008). While bona fide Hh is restricted to metazoans, non-Hh Hog proteins—of unknown function but potentially involved in processes, such as cell division, membrane organization, stress response, or extracellular matrix construction through lipid modification of proteins—are widespread across eukaryotes (Burglin 2008). Of the 10 *Hog* genes (K + Hog and Hog) encoded in the *Cc. yangmingshanensis* genome (17 in *Cz. merolae*, 3 in *Cd. caldarium*, and 2 in *G. partita*), 1 was predominantly expressed in diploid cells, while 8 were predominantly expressed in haploid cells, a result supported by proteome analyses (Fig. 8a). A similar expression pattern of *Hog* genes was also observed in *Cz. merolae* (Fig. 8b). In *Cd. caldarium* and *G. partita*, 2 of the 3 and both *Hog* genes, respectively, were predominantly expressed in haploid cells (Fig. 8b). TFFs are small, protease-resistant proteins that are cosecreted with mucin molecules in the stomach, localize to the gastric mucous layer, and inhibit acidification of surface epithelial cells in mammals (Aihara et al. 2017). Of the 16 *TFF*-like genes (K + TFF and TFF) encoded in the *Cc. yangmingshanensis* genome (15 in *Cz. merolae* and 13 in *Cd. caldarium*), 7 were predominantly expressed in diploid cells, while 5 were predominantly expressed in haploid cells (Data Set S7). However, proteome analyses of *Cc. yangmingshanensi*s showed that many of these proteins are predominantly expressed in the diploid phase (Fig. 8a). Furthermore, in *Cz. merolae* and *Cd. caldarium*, 13 *TFF*-like genes in each species were predominantly expressed in diploid cells (Fig. 8b; Data Sets S8 and S9). Thus, these proteins are likely to function primarily in the diploid phase. The evolutionary origin of *TFF*-like genes remains unclear; however, given that *TFF*s are found only in vertebrates outside of Cyanidiophyceae, it is possible that they arose through convergent evolution to protect cells from low environmental pH in Cyanidiophyceae.

Eisosomes are trough-shaped invaginations of the cell membrane and are found across various eukaryotic lineages (Lee et al. 2015). Recent studies have identified eisosomes as integral components of major stress response pathways in the yeast *S. cerevisiae* (Lanze et al. 2020), although their roles in other organisms remain unclear. Among microalgae, eisosomes have been observed only in cell-walled species, including cell-walled *Cz. merolae* YNP 1A and *G. sulphuraria* CCMEE 5587.1 (Lee et al. 2015). Consistent with this observation, eisosomes were detected in cell-walled diploid cells, but not in cell wall-less haploid cells, of *Cd. caldarium*, *Cc. yangmingshanensis*, and *Cz. merolae* (Fig. 2c; Fig. S1).

In *S. cerevisiae*, Pil1 and Lsp1, which are membrane-sculpting Bin/amphiphysin/Rvs (BAR) domain proteins, have been identified as core components of eisosomes (Walther et al. 2006; Olivera-Couto et al. 2011). Of the 5 *BAR* genes in the *Cc. yangmingshanensis* genome (6 in *Cz. merolae* and *Cd. caldarium*), 1 gene was predominantly expressed in diploid cells, whereas 2 others were predominantly expressed in haploid cells (Fig. 8a), a result supported by proteome analyses (Fig. 8a). A similar expression pattern of *BAR* genes was also observed in *Cz. merolae* and *Cd. caldarium* (Fig. 8b). Thus, the *BAR* genes predominantly expressed in the diploid phase may represent a candidate involved in eisosome formation. In contrast, in *G. partita*, 2 of the 10 *BAR* genes were predominantly expressed in haploid cells, whereas the others showed similar expression levels in both diploid and haploid cells (Fig. 8b), suggesting that these genes may be related to both cell wall invaginations in the diploid phase and/or in other membrane invaginations in the haploid phase.

### Differentially expressed genes underlying differences in the mechanisms of cell division between haploid and diploid cells

Actin is involved in various cellular processes, including cellular motility and cytokinesis in eukaryotes (Blanchoin et al. 2014). Notably, previous studies showed that cytokinesis in the cell-walled *Cc. yangmingshanensis* RK-1 (as demonstrated in this study to be diploid) is accompanied by actin ring formation at the division site, whereas in the cell wall-less *Cz. merolae* 10D (demonstrated above to be haploid), cytokinesis occurs without actin ring formation (Suzuki et al. 1995; Takahashi et al. 1998). In *Cz. merolae* 10D, the endosomal sorting complex required for transport III (ESCRT-III), which is involved in cytokinesis in archaea and animal cells, was shown to mediate cytokinesis (Yagisawa et al. 2020). These findings suggest that distinct cytokinesis mechanisms operate in the diploid and haploid cells of Cyanidiophyceae.

**Figure 9:**
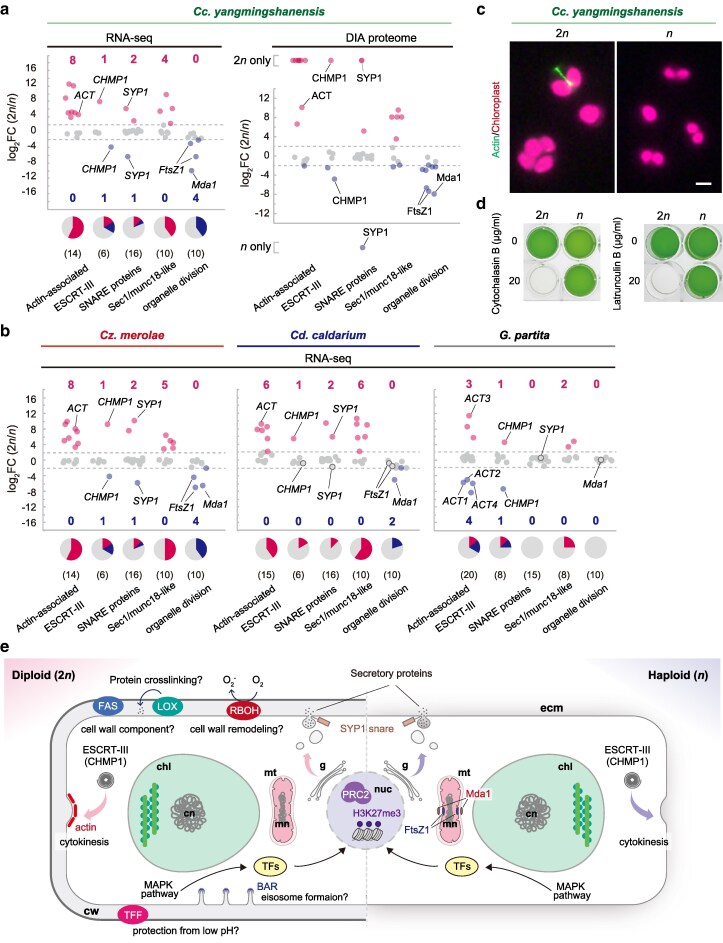
Comparison of transcriptomes of cell division-related genes in Cyanidiophyceae. (a) Ratios of mRNA abundance and the abundance of the corresponding proteins between 2*n* and *n* cells for actin, actin-associated proteins, ESCRT-III components, SNARE proteins, and other functional categories related to cell division in *Cc. yangmingshanensis*. For the RNA-seq results, numbers in parentheses below the pie chart indicate the number of genes in each category. For the proteome results, proteins detected only in 2*n* (2*n* only) or only in *n* (*n* only) are indicated above or below the graphs, respectively. See also Data Set S7 for details. (b) Ratios of mRNA abundance between 2*n* and *n* cells for genes encoding actin, actin-associated proteins, ESCRT-III components, SNARE proteins, and other functional categories related to cell division in *Cz. merolae*, *Cd. caldarium*, and *G. partita*. See also Data Sets S8 to S10 for details. (c) Actin filaments visualized using Alexa Fluor 488 phalloidin in 2*n* and *n* cells (green, phalloidin fluorescence indicating actin filaments; magenta, chloroplast fluorescence). Scale bar: 2 *µ*m. (d) Comparison of sensitivity to actin polymerization inhibitors (cytochalasin B or latrunculin B) between the 2*n* and *n* cells of *Cc. yangmingshanensis*. (e) Schematic representation of the biological features of 2*n* and *n* cells. *chl*, chloroplast; *cn*, chloroplast nucleoid; *cm*, cell membrane; *cw*, daughter cell wall; *ecm*, extracellular matrix; *g*, Golgi apparatus; *mt*, mitochondrion; *mn*, mitochondrial nucleoid; *nuc*, nucleus.

Consistent with these observations, in *Cc. yangmingshanensis*, *Cz. merolae*, and *Cd. caldarium*, a single-copy actin gene and many other genes encoding actin-associated proteins are predominantly expressed in diploid cells (Fig. 9, a and b). Proteome analyses of *Cc. yangmingshanensis* also showed the predominant expression of these proteins in the diploid phase (Fig. 9a). Labeling of F-actin with phalloidin revealed the formation of actin rings at the division plane in diploid cells, but not in haploid cells (Fig. 9c; Fig. S12a). In agreement with this, haploid cells—but not diploid cells—were able to proliferate in the presence of the actin polymerization inhibitors cytochalasin B and latrunculin B (Fig. 9d; Fig. S12b), indicating that diploid cytokinesis, but not haploid cytokinesis, depends on F-actin formation at the division site. In *G. partita*, which possesses 4 actin genes, *ACT3* (orthologous to the *Cc. yangmingshanensis* actin gene) and *ACT2* are required for cytokinesis in diploid cells and for cell motility in haploid cells, respectively (Hirooka et al. 2022).

Among the ESCRT-III components, 2 paralogous *CHMP1* genes encoded in the genomes of *Cc. yangmingshanensis*, *Cz. merolae*, *Cd. caldarium*, and *G. partita* exhibited phase-specific expression: one was predominantly expressed in diploid cells and the other in haploid cells (Fig. 9, a and b). The only minor exception was that, in *Cd. caldarium*, 1 *CHMP1* gene was also predominantly expressed in diploid cells, whereas the other showed slightly higher expression in haploid cells (log_2_FC(2*n*/*n*) = −0.71, below the threshold for differential expression defined in this study) (Fig. 9b). In *Cc. yangmingshanensi*s, proteome analyses also showed these phase-specific expression patterns of CHMP1 proteins (Fig. 9a). A previous study showed that the *CHMP1* gene expressed in haploid cells localizes at the division site during cytokinesis in *Cz. merolae* 10D (Yagisawa et al. 2020). Although the localization and function of the CHMP1 paralog expressed in diploid cells remain unclear, it is likely that distinct ESCRT-III complexes are involved in cytokinesis in the diploid and haploid cells.

Notably, the genomes of *Cc. yangmingshanensis*, *Cz. merolae*, and *Cd. caldarium* do not encode any myosin genes, suggesting that the actin ring involved in diploid cytokinesis functions in the absence of myosin via a mechanism that remains to be elucidated. In *Galdieria*, a single myosin-family gene is encoded in the genome; however, we previously showed that the corresponding protein is not required for cytokinesis (Hirooka et al. 2022).

The soluble N-ethylmaleimide-sensitive factor attachment protein receptor (SNARE) proteins mediate membrane fusion between organelles and between vesicles and the plasma membrane (PM), and their assembly is primarily regulated by Sec1/Munc18-like (SM) proteins (Sudhof and Rothman 2009). Of the 16 genes encoding SNARE proteins in each genome of *Cc. yangmingshanensis*, *Cz. merolae*, and *Cd. caldarium*, 1 of the 2 paralogous *SYP1* genes was predominantly expressed in diploid cells and the other in haploid cells (Fig. 9, a and b). The only minor exception was that, in *Cd. caldarium*, 1 *SYP1* gene was also predominantly expressed in diploid cells, whereas the other showed slightly higher expression in haploid cells (log_2_FC(2*n*/*n*) = −1.73, below the threshold for differential expression defined in this study) (Fig. 9b). Additionally, of the 10 SM genes encoded in each genome of *Cc. yangmingshanensis*, *Cz. merolae*, and *Cd. caldarium*, 4 were predominantly expressed in diploid cells (5 in *Cz. merolae* and 6 in *Cd. caldarium*) (Fig. 9, a and b). In *Cc. yangmingshanensi*s, proteome analyses confirmed that the 2 SYP1 proteins were exclusively expressed in the haploid and diploid phases, respectively, whereas 5 of the SM proteins were predominantly expressed in diploid cells (Fig. 9a). In *Galdieria*, the genome encodes a single *SYP1* gene, which is expressed at similar levels in both diploid and haploid cells, and 8 *SM* (SEC) genes, 2 of which are predominantly expressed in diploid cells (Fig. 9b).

Members of the SYP1 family generally localize to the PM and mediate membrane fusion between the PM and secretory vesicles. In *A. thaliana*, most *SYP1* genes exhibit tissue-specific expression, contributing to the complexity of plant development through membrane trafficking (Enami et al. 2009). KNOLLE (SYP111), a member of the SYP1 family, interacts with the membrane-associated SM protein KEULE (SEC11) at the cell plate and plays a key role in cytokinesis in *A. thaliana* (Waizenegger et al. 2000). In the liverwort *M. polymorpha*, SYP12A, a member of the SYP1 family, is required for cell plate formation during cytokinesis, functioning similarly to KNOLLE (Kanazawa et al. 2020).

Based on these findings, in *Cyanidiococcus*, *Cyanidioschyzon*, and *Cyanidium*, the phase-specific expression of the 2 *SYP1* paralogs may be associated with differences in the secretory pathway, which in turn may lead to the formation of distinct extracellular matrices between diploid and haploid cells. Additionally, 1 or more SM proteins that are highly expressed in diploid cells may interact with SYP1 and contribute to the construction of a nascent cell wall during cytokinesis. By contrast, in *Galdieria*, although only a single *SYP1* gene is present and expressed in both diploid and haploid cells, the difference in the expression patterns of *SM* genes likely results in a difference in extracellular matrix formation between the diploid and haploid phases.

Finally, while it is unclear how this impact manifests as phenotypic differences, we found that 2 types of *FtsZ1* genes (each a single-copy gene) and the *Mda1* gene (also a single-copy gene), but not the dynamin-related gene (*DRP3*), which are responsible for mitochondrial division (Nishida et al. 2003, 2007), are predominantly expressed in the haploid phase in *Cc. yangmingshanensis*, *Cz. merolae*, and *Cd. caldarium* (Fig. 9, a and b). In *Cc. yangmingshanensis*, proteome analyses also showed the predominant expression of these proteins in the haploid phase (Fig. 9a). These results suggest that the haploid and diploid phases may use different mechanisms for mitochondrial division, though further analysis is needed. Regarding *Galdieria*, which possesses a net-like mitochondrion unlike other cyanidiophycean genera with a single disc-shaped mitochondrion, it has lost the mitochondrial *FtsZ* gene during evolution, and the *Mda1* as well as the *DRP3* gene exhibited no differences in expression between the haploid and diploid phases (Fig. 9b; Data Set S10).

In summary, multiple candidate genes involved in the phenotypic differences between diploid and haploid phases in cyanidiophycean algae were identified (Fig. 9e). In particular, several genes, including those encoding the MADS-box TF, MAPK cascade components, actin, and the ESCRT-III subunit CHMP1, exhibited similar phase-specific expression patterns across Cyanidiophyceae. These results suggest that the functions of these genes were retained from the last common ancestor of Cyanidiophyceae. The functions of these proteins in Cyanidiophyceae may reflect their ancestral features in Archaeplastida, which will be elucidated by further functional analyses through genetic modifications in Cyanidiophyceae.

## Discussion

### Revisiting Cyanidiophyceae following the discovery of their sexual life cycle

**Figure 10:**
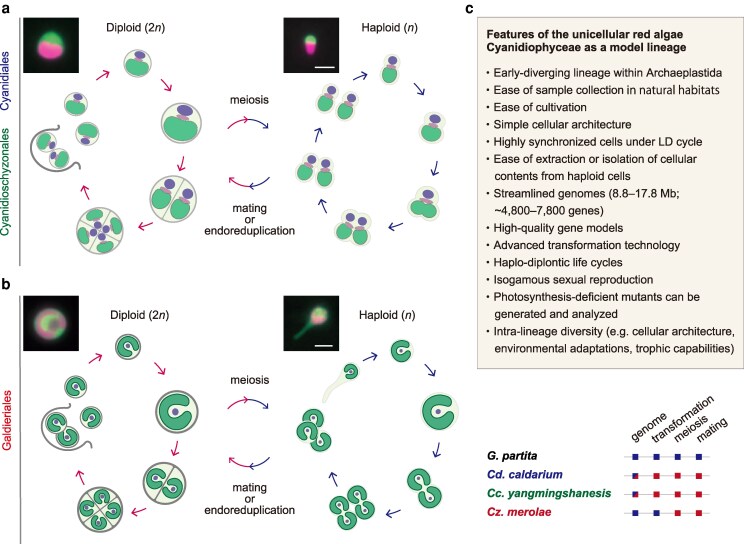
The sexual life cycle and model-lineage characteristics of the unicellular red algae Cyanidiophyceae. (a and b) Haplo-diplontic life cycle of *Cyanidium* (Cyanidiales), *Cyanidiococcus* and *Cyanidioschyzon* (both Cyanidioschyzonales) (a), and *Galdieria* (Galdieriales) (b), showing the proliferation modes 2*n* and *n* cells. These cyanidiophycean algae can undergo sexual reproduction under laboratory conditions, enabling the generation of stable diploid transformants through mating between haploid transformants. The upper panels show transformants expressing the fluorescent protein Venus in the cytoplasm. Scale bar: 2 *µ*m. (c) Summary of features and experimental techniques available for Cyanidiophyceae. In this study, we established that all 4 axenically cultivable genera of Cyanidiophyceae now have access to high-quality genomic resources, genetic manipulation methods, and protocols for inducing meiosis (to generate *n* cells) and mating (to generate heterozygous 2*n*). Blue boxes indicate presence based on previous studies, and red boxes indicate presence based on this study.

Sexual reproduction had not been observed in unicellular red algae, despite their long history of research. Recently, we discovered a sexual life cycle in *Galdieria*, the earliest-branching genus in the unicellular red algal class Cyanidiophyceae (Hirooka et al. 2022). In this study, we further elucidate that 3 additional genera of Cyanidiophyceae—*Cyanidium*, *Cyanidiococcus*, and *Cyanidioschyzon*—also have a sexual life cycle, alternating between the cell-walled diploid and the cell wall-less haploid (Fig. 10).

Thus far, inconsistencies between morphological and molecular phylogenetic classifications have caused systematic problems in the classification of Cyanidiophyceae. The discovery of the sexual life cycle has revealed that the traditional classification that only *Cyanidioschyzon* among Cyanidiophyceae lacks a cell wall and proliferates by binary fission, whereas *Cyanidiococcus* and *Cyanidium* possess a cell wall and proliferate by forming 4 daughter cells within the mother cell (Fig. 1, b and c), has turned out to be incorrect. As shown in this study, the diploid forms, as well as the haploid forms, of these 3 genera are morphologically indistinguishable from one another under light microscopy (Fig. 2b). Instead, based on current knowledge, the major differences among the 3 genera, classified by molecular phylogenetic analysis (Park et al. 2023), include large variations in genome size (Fig. 1b) (Cho et al. 2023) and partial differences in gene content (Fig. 3a). The thicker cell wall of *Cyanidium*, compared to the other 2 genera (Fig. 2c; Fig. S1), as well as its lower optimal temperature and pH (Fig. 4f), may serve as distinguishing characteristics for *Cyanidium* in relation to *Cyanidiococcus* and *Cyanidioschyzon*, although further investigation of additional strains is needed.

Furthermore, it has become clear that *Cz. merolae* 10D, the most extensively studied cell wall-less strain among Cyanidiophyceae (Kuroiwa et al. 2018; Miyagishima and Tanaka 2021), should be recognized as a haploid clone. With this clarification, the long-standing mystery from previous genome analyses of *Cz. merolae* 10D—namely, the presence of numerous genes that show no detectable mRNA expression despite being conserved across other eukaryotic lineages (such as genes encoding actin and its regulatory proteins) (Matsuzaki et al. 2004)—can now be reasonably explained. This study reveals that many of these genes are, in fact, specifically expressed in the diploid phase (Fig. 7a; Fig. S11).

In this study, our field sampling in Japanese sulfuric hot springs revealed that only the cell-walled diploid forms of *Cyanidiococcus* and *Galdieria* were present in the blue–green algal mats attached to submerged rocks and in endolithic habitats and mud deposits above the water surface (Fig. 4a; Fig. S5, a to f). Furthermore, we reanalyzed recently published diurnal metatranscriptomic (RNA-seq) data from Yellowstone National Park, United States—specifically, cyanidiophycean mats in a stream dominated by *Cz. merolae* sequences and terrestrial mats on mud, where *G. yellowstonensis* sequences were second in abundance after *Cz. merolae* (Stephens et al. 2024). Even in these datasets, diploid-specific genes (identified in this and previous studies) were detected (Fig. S13a), whereas haploid-specific gene sets showed almost no detectable expression (Fig. S13b). Supporting these results, microscopic observations of the *Cz. merolae* mat in the stream revealed only cell-walled round cells (Bhattacharya et al. 2025).

The cell wall-less *Cz. merolae* was originally isolated from hot spring samples collected in Naples, Italy, after propagation of algae in the samples in an inorganic, nutrient-rich medium at pH 1.5 (De Luca et al. 1978). The cell wall-less strain *Cz. merolae* 10D was subsequently isolated from a similar sample collected in Sardinia, Italy (Toda et al. 1995). Thus, these strains were most likely originally cell-walled diploids in nature, which underwent meiosis and produced cell wall-less haploids during cultivation in nutrient-rich media prior to the isolation of haploid clones.

Thus, to date, there have been no confirmed observations of the haploid phase of Cyanidiophyceae in natural habitats. When, where, and under what environmental conditions haploid cells are formed remain open questions for future investigation, although in this study, they were generated at low frequency in an inorganic, nutrient-rich medium with a lower pH (1.0 or 0.75) than the standard cultivation conditions for Cyanidiophyceae (∼pH 2.0).

### Utility of Cyanidiophyceae as a model lineage for studying eukaryotic evolution, photosynthesis, life cycles, and phenotypic diversification

In this and our recent (Hirooka et al. 2022) study, taking advantage of the lack of a cell wall in the haploid phase of Cyanidiophyceae, PEG-mediated DNA introduction—originally developed for genetic modification of *Cz. merolae* 10D—has now been successfully applied to all 4 axenically cultivable genera of Cyanidiophyceae. Together with genomic and transcriptomic data, this genetic manipulation technology provides a powerful platform for studying the evolution and minimal requirements for the construction and function of photosynthetic eukaryotic cells, as well as the adaptation and colonization of eukaryotes into new environments and other biological processes, based on the following features.

1. Cyanidiophyceae possess the simplest cellular and genomic structures among known photosynthetic eukaryotes. These cells contain only a minimal set of membrane-bound organelles (Kuroiwa et al. 2018), and their nuclear genomes encode ∼4,800 to 7,800 genes. Their cell cycles can be tightly synchronized with the diel cycle, even in natural habitats (Suzuki et al. 1994; Fujiwara et al. 2009; Jong et al. 2021; Stephens et al. 2024). Additionally, haploid cells of Cyanidiophyceae lack a rigid cell wall, which facilitates the extraction and isolation of cellular contents, including intact organelles, as demonstrated in *Cz. merolae* 10D (Miyagishima et al. 1999; Yagisawa et al. 2009). The simple genome facilitates comprehensive omics, such as transcriptomics and proteomics. For example, DIA proteome analyses of *Cc. yangmingshanensis* detected more than 90% of the predicted proteins, providing near-complete coverage of the expressed proteome (Data Set S7). Taken together, these features enable studies on cell cycle and organelle biology, including interorganelle studies.
2. Cyanidiophyceae are widely distributed worldwide, yet the extent and distribution of genetic diversity in this lineage remain poorly understood. In this study, we quantified genetic divergence in 3 genera and revealed substantial differences in haplotype divergence among these algae. Notably, *Cc. yangmingshanensis* NIES-4659 exhibited relatively high haplotype divergence (2.19%) compared to the other genera examined. However, *k*-mer–based estimation of heterozygosity among additional *Cyanidiococcus* strains revealed substantial variation, ranging from approximately 0.146% (KS-11) to 1.72% (KS-5) (Fig. 4e), indicating marked genetic divergence within the genus *Cyanidiococcus*. Given the phylogenetic subdivision within *Cyanidiococcus* (Fig. 4e) (Huang et al. 2024), integrating mating experiments with comparative genomics may provide further insight into the extent of reproductive compatibility and genetic differentiation among these lineages. In addition, comparative analyses incorporating chromosomal architecture and environmental conditions may further contribute to our understanding of the evolution of genetic diversity in Cyanidiophyceae. Overall, the worldwide distribution of Cyanidiophyceae, their ease of sampling, and their streamlined genomes make them a promising model system for investigating the evolutionary processes shaping genetic diversity.
3. A variety of genetic modification techniques can be applied to the streamlined genomes of Cyanidiophyceae. Because most proteins are encoded by single-copy genes (except for *Galdieria*; Fig. 3c), phenotypic analyses are relatively straightforward. Marker recycling enables editing of multiple loci (Fig. 5, g to i; Fig. S6, g to j), and haploid clones can be converted into diploids as needed (Fig. 10, a and b). Strategies developed for *Cz. merolae* 10D—such as gene knockouts, epitope tagging, inducible expression, inducible knockdown, and CRISPR/Cas9-based editing—are in principle applicable to all strains. Currently, generating transformants of *Cc. yangmingshanensis* and *Cd. caldarium* takes over a month; however, this process could be shortened by referring to a recently optimized protocol that enables transformant preparation in *Cz. merolae* 10D within 2 wk (Villegas-Valencia et al. 2025). Thus, while further optimization is needed, these technologies are expected to facilitate functional genomic analyses across Cyanidiophyceae.
4. Both diploid and haploid cells undergo stable asexual proliferation, representing a haplo-diplontic life cycle. This feature allows straightforward preparation of samples and direct comparison between the 2 phases. In addition, a recent study reported that 50% of protists distributed across the entire eukaryotic evolutionary tree possess a haplo-diplontic life cycle, suggesting that this may be an ancestral feature of eukaryotes (Rizos et al. 2024). Thus, studying the life cycles of Cyanidiophyceae may provide insights into the characteristics and evolution of the original eukaryotic life cycle.
5. Photosynthesis-deficient mutants can be generated and analyzed. The red algal photosynthetic apparatus exhibits intermediate characteristics between those of cyanobacteria and Viridiplantae (Allen et al. 2011) and contains lineage-specific components of unknown function (some also present in organisms with secondary red plastids) (Sturm et al. 2013; Lee et al. 2019). However, molecular genetic studies of photosynthesis-related genes have not been feasible in red algae, limiting their functional analyses. We recently succeeded in generating knockout mutants of genes involved in photosynthetic pigment biosynthesis in *G. partita* (Hirooka et al. 2022). This was possible because *Galdieria* can uniquely grow photoautotrophically, mixotrophically, and heterotrophically, unlike strictly photoautotrophic genera, such as *Cyanidium*, *Cyanidiococcus*, and *Cyanidioschyzon* (Gross and Schnarrenberger 1995; Barbier et al. 2005). Notably, a transgenic *Cz. merolae* expressing a *Galdieria* PM sugar transporter showed heterotrophic growth in glucose-supplemented medium (Fujiwara et al. 2019). Thus, these features make Cyanidiophyceae a valuable model for uncovering unknown mechanisms of photosynthesis.
6. Cyanidiophyceae exhibit intralineage diversity in environmental adaptations, trophic capabilities, cellular architectures, and organelle inheritance. After diverging from the common ancestor of other red algae around 1.5 billion years ago, Cyanidiophyceae diversified (Yoon et al. 2004; Cho et al. 2023; Park et al. 2023; Huang et al. 2024). Today, many strains exist, each adapted to distinct, isolated hot, acidic springs—or even to distinct microenvironments within the same location. For example, *G. phlegrea* is adapted to endolithic layers (Qiu et al. 2013). Further, as shown in this study, even among thermo-acidophilic strains, optimal growth conditions and metal tolerance vary (Cho et al. 2023) (Fig. 4f; Fig. S5d). Some species have secondarily adapted to mesophilic environments (eg the genus *Cyanidiofrigus*; (Huang et al. 2024)), or even to neutral environments (eg 3 genera: *Gronococcus*, *Cavernulicola*, and *Sciadococcus*; (Park et al. 2023)), although their axenic cultures have not yet been established and genomic information is still lacking. Remarkably, 18S rDNA sequences related to Cyanidiophyceae have been detected in global ocean samples (*Tara* Oceans project; https://www.gbif.org/species/334), raising the possibility that additional, yet undiscovered, diversity may exist within Cyanidiophyceae. Thus, comparative analyses of the currently cultivable cyanidiophycean strains exhibiting diverse environmental adaptations, together with the future establishment of axenic cultures of cyanidiophycean algae inhabiting mesophilic environments, are expected to facilitate studies of adaptive evolution in Cyanidiophyceae. In addition, one of the major differences within Cyanidiophyceae is trophic capability: *Galdieria* is capable of heterotrophic growth, and in some strains, the chloroplast (plastid) can reversibly lose the photosynthetic apparatus and thylakoid membrane, whereas other genera are obligate photoautotrophs, possibly having lost this capacity altogether (Gross and Schnarrenberger 1995; Barbier et al. 2005; Yamashita et al. 2025). Furthermore, Cyanidiophyceae exhibit diversity in other traits, such as the morphology of mitochondria and vacuoles (Fig. 1c) (Albertano et al. 2000), and patterns of mtDNA inheritance (Fig. S10). Comparative genomic analyses have suggested that gene losses, HGTs—including those encoding heavy metal detoxification enzymes and heat shock proteins—and de novo gene emergence, all occurring in a lineage- or strain-specific manner, likely underlie the phenotypic diversification observed across Cyanidiophyceae (Van Etten et al. 2023). To further clarify the genetic bases of phenotypic diversification, functional approaches—such as gene knockout or gene transplantation (for which this study developed a method applicable to all culturable strains), exemplified by the expression of a *Galdieria* sugar transporter in *Cyanidioschyzon* in a previous study (Fujiwara et al. 2019)—will be particularly useful. Collectively, these features establish Cyanidiophyceae as a model lineage for elucidating phenotypic diversification in eukaryotes.

### The Cyanidiophyceae as a basis for understanding the origin of complex multicellular sexual life cycles and associated phenomena in Archaeplastida

Sexual reproduction has been observed in multicellular red algae, certain lineages of both multicellular and unicellular green algae, and land plants. The sexual life cycle of Cyanidiophyceae is the only one known among unicellular red algae and represents the earliest-diverging example found within Archaeplastida to date (Fig. 1a). Thus, it would provide crucial insights into the evolution of multicellular sexual life cycles with cellular differentiation, which independently arose in red algae and Viridiplantae (Fig. 1a), as well as into the broader evolution of such systems in eukaryotes.

In this study, we found that 159 (3.3%) and 91 (1.9%) protein-coding genes in the nuclear genome (4,884 protein-coding genes) are predominantly expressed in the diploid and haploid phases, respectively, in *Cc. yangmingshanensis* (Fig. 7a). Some of these genes are reasonably correlated with phenotypic differences between the 2 phases, such as the presence or absence of a cell wall and the requirement for actin in cell division (Fig. 9e). Furthermore, the set of differentially expressed genes includes members of TF families that are known in plants to be involved in the formation of reproductive structures and other developmental processes, and the number of such genes is considerably smaller compared to plants (Fig. 7d). Similar results were also observed in *Cz. merolae*, *Cd. caldarium*, and *G. partita* (Fig. 7e). In addition, most of these haploid- or diploid-predominant genes are marked with H3K27me3 in the opposite phase (Fig. 7, b and c).

H3K27me3 is a histone modification catalyzed by the Polycomb Repressive Complex 2 (PRC2), which mediates gene silencing during differentiation and development in multicellular organisms, such as animals and plants. In contrast, the PRC2 complex is absent in the yeasts *S. cerevisiae* and *S. pombe*, which previously led to the assumption that PRC2 and H3K27me3 may have evolved alongside the emergence of multicellularity to regulate cellular differentiation. However, components of PRC2 and its associated mark (H3K27me1/-me2/-me3) have been later identified in several lineages of unicellular eukaryotes (Shaver et al. 2010; Mikulski et al. 2017; Frapporti et al. 2019; Zhao et al. 2021; Petroll et al. 2025b), suggesting that yeasts secondarily lost PRC2. To date, the function of H3K27me3 has been investigated in only a few unicellular eukaryotes. In the diatom *P. tricornutum* (a diploid clone) (Filloramo et al. 2021) and in *Cz merolae* 10D (a haploid clone), H3K27me3 predominantly marks transposable elements (TEs) and repeat sequences, but is also found on certain protein-coding genes (Hisanaga et al. 2023). In *P. tricornutum*, genes involved in cell morphological changes were found to be marked by H3K27me3, and knockout of the *enhancer of zeste* (*E(z)*) gene, which encodes the catalytic component of the PRC2 complex, abolished normal cell morphology, suggesting that H3K27me3 regulates cell differentiation in this unicellular organism (Zhao et al. 2021). However, unlike in *P. tricornutum*, knockout of *E(z)* gene in *Cz. merolae* 10D resulted in the expression of TEs, but no obvious changes in cellular morphology were observed (Hisanaga et al. 2023). Thus, in addition to H3K27me3, other histone modifications and/or the expression of specific TFs may also be required for the phenotypic differences between the haploid and diploid phases.

KNOX and BELL-related TFs are expressed in gametes and are involved in the haploid-to-diploid transition in *C. reinhardtii* and *M. polymorpha* (Lee et al. 2008; Hisanaga et al. 2021), and more recently have also been shown to be required for this transition in *Galdieria* (Hirooka et al. 2022), which may reflect their ancestral roles in the Archaeplastida. However, in this study, we were not able to detect BELL-related or KNOX proteins in either haploid or diploid cells of *Cc. yangmingshanensis* by proteome analysis, although KNOX mRNA was predominantly detected in the haploid phase of *Cyanidioschyzon* and *Cyanidiococcus* (Fig. 7, d and e). These results raise the possibility that the haploid cells examined in this study are likely in a vegetative state and may transform into as-yet-unknown gametes expressing KNOX and BELL proteins upon a certain stimulus. In this regard, among the *Galdieria* haploid cell population, cells before cell division phase formed tail-like membrane protrusions in which actin and microtubules were organized (tadpole-shaped cells), and these cells migrated across surfaces at approximately 25 *µ*m/min (Hirooka et al. 2022). In contrast, the haploid cells of *Cz. merolae* 10D moved much more slowly, at only around 4 *µ*m/min (Ohnuma et al. 2011). On the other hand, a recent study showed that increasing the salt concentration of the *Cz. merolae* culture medium induces, within 10 min, the formation of protrusions from the part of the cell membrane containing actin filaments on the cytoplasmic side (termed “tentacles”), which in turn appear to accelerate cell motility (Maschmann et al. 2020). Thus, if vegetative haploids can be induced to form gamete through such a process, it will be important in future studies to distinguish and analyze these states separately.

In many eukaryotes, organelle DNA is inherited from only 1 parent. There is no single mechanism underlying uniparental inheritance; rather, diverse mechanisms operate at different stages of reproduction in different species (Birky 1995). For example, in *C. reinhardtii*, cpDNA from the minus mating type is selectively degraded by an as-yet-unidentified nuclease within minutes after gametic fusion (Kuroiwa et al. 1982; Nishimura et al. 1999). In *S. cerevisiae*, rapid segregation from heteroplasmy to homoplasmy within a few tens of generations has been attributed to asymmetric partitioning of mtDNA copies between daughter cells (Roussou et al. 2024), possibly facilitated by concatemer formation through rolling-circle DNA replication (Ling and Shibata 2004). In both species, parental organelles fuse in the zygote, and the mixed genomes subsequently become biased toward 1 parental type. By contrast, in the brown alga *Ectocarpus*, each gamete contains a single chloroplast, and after isogamous mating, the 2 parental chloroplasts remain physically separate in the zygote. They are segregated into different daughter cells at the first sporophytic division, resulting in random fixation of 1 parental chloroplast (Peters et al. 2004; Choi et al. 2020). In Cyanidiophyceae, we found that—except for mtDNA in *Galdieria*—both mtDNA and cpDNA are initially inherited biparentally, producing a heteroplasmic state. During diploid proliferation, this heteroplasmy resolves stochastically into homoplasmy, with 1 parental genome retained at random (Fig. 6i; Fig. S10). Thus, Cyanidiophyceae resemble *S. cerevisiae* and *Ectocarpus*—although the underlying mechanisms differ between those species—rather than the strictly uniparental pattern seen in *C. reinhardtii* and many other eukaryotes. In *Galdieria*, however, mtDNA from only 1 mating type is retained, indicating the presence of an active mechanism. The molecular basis of these processes in Cyanidiophyceae remains unclear. In particular, it will be essential to determine whether organelle fusion occurs in the zygote and, if so, how mitochondrial and chloroplast genomes behave subsequently. This will require direct observation of mitochondrial and chloroplast dynamics during and after gametic fusion.

Further elucidation of the mechanisms underlying sexual reproduction in Cyanidiophyceae—including those that regulate phenotypic transitions between the haploid and diploid phases, as well as those governing organelle DNA inheritance—will require observation of both mating and meiosis, which we were unable to achieve in this study due to their substantially low frequency in our cultivation conditions. Thus, further studies are needed to identify conditions that increase the frequency of mating and meiosis.

## Materials and methods

### Algal strains and culture conditions

Many cyanidiophycean algal strains have been maintained in stock centers, such as the Microbial Culture Collection at the National Institute of Environmental Studies (NIES), the Sammlung von Algenkulturen Culture Collection of Algae (SAG), the Culture Collection of Cryophilic Algae (CCCryo), and the Algal Collection University Federico II (ACUF). However, some cyanidiophycean strains are not clonal cultures and are contaminated with other strains. For example, *G. sulphuraria* 3377 was isolated from a *Cz. merolae* culture as a green colony that arose on glucose-supplemented plates in the dark (Masson et al. 2026). Likewise, *Cz. merolae* Soos was isolated from a *G. phlegrea* Soos culture as a strict photoautotroph that outgrew the *Galdieria* strain under photoautotrophic conditions (Rossoni et al. 2019). Due to contamination issues in some strains, caution is required when using cyanidiophycean algal cultures. Thus, in this study, single clones of all strains obtained from stock centers were reisolated prior to analyses.

*Cc. yangmingshanensis* N3110 (NIES-4659) was isolated from a sample collected in an acidic hot spring in Hakone, Kanagawa Prefecture, Japan, and is deposited in the NIES Collection. The other *Cyanidiococcus* strains (heterozygous diploids; CcyaKS-1, CcyaKS-5, CcyaKS-11, TS5, TS6, TH2, TH3, and TH12) were isolated from samples collected in acidic hot springs in Kusatsu, Gunma Prefecture (Sunada et al. 2025), or Tsukahara, Oita Prefecture, Japan, and have been stocked at the Symbiosis and Cell Evolution Laboratory, National Institute of Genetics (NIG). *Cz. merolae* MS1 (heterozygous diploid) was isolated from a sample collected in an acidic hot spring in Yellowstone National Park, Wyoming, United States, and is stocked at the Arizona Center for Algae Technology and Innovation, Arizona State University, and at the Symbiosis and Cell Evolution Laboratory, NIG. *Cd. caldarium* NIES-551 (heterozygous diploid) and *Cz. merolae* 10D (NIES-3377; haploid) were obtained from the NIES Collection. *Cd. caldarium* SAG-16.91 (heterozygous diploid) was obtained from the SAG. *G. partita* NBRC102759 (heterozygous diploid) was obtained from the Biological Resource Center, NITE (NBRC). The haploid strain *G. partita* NBRC102759 N1 (Hirooka et al. 2022), derived from *G. partita* NBRC102759, is stocked at the NIG.

These strains were maintained in 20 mL of MA medium at pH 2.0 (for *Cd. caldarium* NIES-551, *Cc. yangmingshanensis* NIES-4659, other *Cyanidiococcus* strains, *Cz. merolae* 10D, *Cz. merolae* MS1, and *G. partita* NBRC102759) or at pH 1.0 (for *G. partita* NBRC102759 N1) in 25 cm^2^ tissue culture flasks (TPP Techno Plastic Products, Trasadingen, Switzerland), statically in a 2% CO_2_ incubator at 37 °C for *Cd. caldarium* NIES-551 and at 42 °C for the other strains under continuous light (30 *μ*mol photons m^−2^ s^−1^).

To generate haploid cells from the diploid clones (*Cd. caldarium* NIES-551, *Cc. yangmingshanensis* NIES-4659, and *Cz. merolae* MS1), cells in MA medium at pH 2.0 were transferred to 1 mL of MA medium at pH 1.0 (or pH 0.75 for *Cd. caldarium*) in a 24-well plate (1 mL/well). The cells were cultivated statically in a 2% CO_2_ incubator at 37 °C for *Cd. caldarium* and at 42 °C for the other strains under continuous light (30 *μ*mol photons m^−2^ s^−1^) for 1 to 3 wk. In the resulting culture, cell wall-less haploid cells were generated and mixed with cell-walled diploid cells. A single haploid cell was isolated and transferred into 1 mL of IMA medium at pH 0.75 for *Cd. caldarium* and at pH 1.2 for the other strains. The IMA medium contained 20 mM (NH_4_)_2_SO_4_, 4 mM MgSO_4_·7H_2_O, 4 mM KH_2_PO_4_, 1 mM CaCl_2_, 5 mM FeSO_4_·7H_2_O, and trace elements as described in Minoda et al. (2004). The isolated haploid cell was cultured in 1 well of a 24-well plate (1 mL/well) statically in a 2% CO_2_ incubator under continuous light to generate a clonal haploid population. The haploid strains are stocked at the Symbiosis and Cell Evolution Laboratory, NIG. Each haploid clone of the respective strains is maintained in 20 mL of MA medium at pH 2.0 (for *Cc. yangmingshanensis* NIES-4659 Ha1 and *Cz. merolae* MS1 Ha3) or at pH 0.5 or 0.75 (for *Cd. caldarium* NIES-551 Ha5) in 25 cm^2^ tissue culture flasks, statically in a 2% CO_2_ incubator at 37 °C for *Cd. caldarium* NIES-551 Ha5 and at 42 °C for the other strains under continuous light (30 *μ*mol photons m^−2^ s^−1^).

To determine the optimal temperature condition, cells were cultured at various temperatures (ranging from 25 to 45 °C). Growing cells were inoculated into 20 mL of MA at pH 0.75 (for *Cd. caldarium* NIES-551 Ha5), pH 1.0 (for *G. partita* NBRC102759 N1), and pH 2.0 (for the other strains) in 25 cm^2^ tissue culture flasks to give an OD_750_ of 0.2. The cells were cultured on a reciprocal shaker at 120 rpm (NR-2; TAITEC) in an incubator (MIR-154-PJ; PHC) under ambient air and continuous light conditions (50 *μ*mol photons m^−2^ s^−1^) for 7 d. To determine the optimal pH condition, cells were cultured in MA medium at 7 different pH values (ranging from pH 0.1 to pH 3.0). The pH of the medium was adjusted using H_2_SO_4_. Growing cells were harvested by centrifugation at 1,500 × *g* for 5 min and then resuspended in 1 mL of the respective medium in each well of a 24-well plate to give an OD_750_ of 0.2. The cells were then cultured statically in a 2% CO_2_ incubator at 37 °C for *Cd. caldarium* and *G. partita* and at 42 °C for the other strains under continuous light (50 *μ*mol photons m^−2^ s^−1^) for 7 d.

To determine arsenate (KH_2_AsO_4_) and nickel (NiSO_4_·6H_2_O) tolerance, cells were cultivated in media containing a series of concentrations of the respective metals (ranging from 0 to 50 mM). Growing cells were harvested by centrifugation at 1,500 × *g* for 5 min and then resuspended in 1 mL of the respective medium at pH 0.75 in each well of a 24-well plate to give an OD_750_ of 0.2. The cells were cultured statically in a 2% CO_2_ incubator at 37 °C for *Cd. caldarium* and at 42 °C for the other strains under continuous light (50 *μ*mol photons m^−2^ s^−1^) for 7 d. OD_750_ values were measured using a spectrophotometer (BioSpectrometer basic; Eppendorf, Germany). Growth rates based on OD_750_ were determined as described by Hirooka et al. (2014).

To determine the sensitivity of the *CP^r^-HSVtk* strain to ganciclovir and 5-FdU, cells were cultivated in media containing a series of concentrations of ganciclovir (ranging from 0 to 2 mg/mL) and 5-FdU (from 0 to 100 *μ*g/mL). Growing cells were harvested by centrifugation at 1,500 × *g* for 5 min and then resuspended in 1 mL of the respective medium in each well of a 24-well plate to give an OD_750_ of 0.2. The cells were then cultured statically in a 2% CO_2_ incubator at 42 °C under continuous light (30 *μ*mol photons m^−2^ s^−1^) for 7 d.

To compare the sensitivity to actin polymerization inhibitors between diploid and haploid cells of cyanidiophycean strains, original diploid clones (*Cc. yangmingshanensis* NIES-4659 and *Cz. merolae* MS1) and haploid clones (*Cc. yangmingshanensis* NIES-4659 Ha1, *Cz. merolae* MS1 Ha3, and *Cz. merolae* 10D), grown in MA medium at pH 2.0, were inoculated into 1 mL of MA medium at pH 2.0 supplemented with 20 *µ*g/mL cytochalasin B or latrunculin B to give an OD_750_ of 0.2. For the *Cd. caldarium* strains (*Cd. caldarium* NIES-551 and *Cd. caldarium* NIES-551 Ha5), cells were grown at 37 °C in MA medium at pH 0.75 and inoculated into 1 mL of MA medium at pH 0.75 supplemented with 50 *µ*g/mL cytochalasin B or latrunculin B. Stock solutions of cytochalasin B (10 mg/mL; FUJIFILM Wako Pure Chemical Corp., Osaka Japan) and latrunculin B (5 mg/mL; Sigma-Aldrich) were prepared in DMSO. DMSO was used as a control. The respective cells were cultured statically in 1 well of a 24-well plate in a 2% CO_2_ incubator at 42 °C for 1 wk (*Cc. yangmingshanensis* and *Cz. merolae*) or at 37 °C for 2 wk (*Cd. caldarium*), under continuous light (30 *μ*mol photons m^−2^ s^−1^).

### Microscopy

To observe cyanidiophycean cells using differential interference contrast (DIC) and fluorescence microscopy, to compare nuclear DNA content, and to visualize actin filaments, cells were cultured statically in 20 mL of MA medium, adjusted to the optimal pH for each strain, in 25 cm^2^ tissue culture flasks. Cultures were maintained in a 2% CO_2_ incubator at the optimal temperature for each strain under continuous light (30 *μ*mol photons m^−2^ s^−1^).

To compare nuclear DNA content, a 0.5 mL sample of cells cultured under continuous light was fixed with 0.1% glutaraldehyde at room temperature for 10 min. The fixed cells were centrifuged at 1,500 × *g* for 5 min, and the cell pellet was resuspended in 1 mL of 50% (v/v) ethanol and incubated at room temperature for 5 min. The cells were then harvested by centrifugation and resuspended in 0.5 mL of 70% (v/v) ethanol. After centrifugation, the cells were resuspended in 0.5 mL of phosphate-buffered saline (PBS), and DAPI was added to a final concentration of 1 *µ*g mL^–1^. Nuclear DNA content was quantified using nuclei from Type I cells (ovoid cells corresponding to the G1 phase), as defined by Fujiwara et al. (2009), in cell wall-less (haploid) cells, and nuclei at the 4-cell stage (G1 phase just after 2 rounds of cell division) in cell-walled (diploid) cells; both correspond to a 1C nuclear state.

To visualize actin filaments, 0.1 mL of cultured cells was fixed with 2% paraformaldehyde at room temperature for 10 min and then centrifuged at 1,500 × *g* for 5 min. The fixed cells were washed twice with 0.5 mL of PBS, each followed by centrifugation at 1,500 × *g* for 5 min. The cell pellets were resuspended in 1 mL of ice-cold acetone, incubated at −20 °C for 10 min, and centrifuged again at 1,500 × *g* for 5 min. The cells were then resuspended in 0.1 mL of PBS, followed by the addition of 2.5 *μ*L of Alexa Fluor 488 phalloidin.

Images of the cells were captured using a BX51 upright microscope (Olympus, Tokyo, Japan) equipped with a DP71 digital camera (Olympus). To detect Venus/Alexa Fluor 488 phalloidin, chloroplast fluorescence, and DAPI-stained DNA, the NIBA, WIG, and U-MWU filter sets (Olympus) were used, respectively. The fluorescence intensity of DAPI-stained DNA was measured using ImageJ ver. 1.53a (Schneider et al. 2012).

To observe cyanidiophycean cells using transmission electron microscopy, cells were cultured in MA medium adjusted to the optimal pH for each strain in 25 cm^2^ tissue culture flasks on a rotary shaker (120 rpm) in a 5% CO_2_ incubator at 42 °C under continuous light (50 *μ*mol photons m^−2^ s^−1^). Preparation of samples and observations were performed as previously described for haploid and diploid cells of *Cc. yangmingshanensis* (Ichinose and Iwane 2018; Hirooka et al. 2022), with minor modifications and for those of *Cz. merolae*, *Cd. caldarium* and *G. partita* (Yamashita et al. 2025).

### Phylogenetic analysis

The amino acid and nucleotide sequences were aligned using MAFFT ver. 7.212 (Katoh and Standley 2013), and ambiguous sites were excluded using Gblocks ver. 0.91b (Castresana 2000). Substitution models were selected using ModelTest-NG ver. 0.1.7 (Darriba et al. 2020) (LG + G for the amino acid sequences of RbcL in Fig. 2a; HKY + G and GTR + G for the nucleotide sequences of *rbcL* in Fig. 4e and Fig. S8d, respectively). Maximum likelihood (ML) analysis and 1,000 pseudoreplicates of bootstrap analysis were performed using RAxML-NG ver. 1.0.3 (Kozlov et al. 2019). Bayesian analyses were performed using MrBayes 3.2.7 (Ronquist et al. 2012) with the optimal substitution models, 1,000,000 generations of Markov chain Monte Carlo iterations, and a burn-in of 25%.

### DNA extraction and sequencing

For PacBio long-read sequencing, haploid cells (*Cc. yangmingshanensis* NIES-4659 Ha1, *Cd. caldarium* NIES-551 Ha5, and *Cz. merolae* MS1 Ha3) were harvested by centrifugation at 2,000 × *g* for 5 min from 10 mL cultures (OD_750_ ∼5.0). High-molecular-weight genomic DNA was extracted from the harvested cells according to Hirooka et al. (2022). Following the manufacturer's instructions, the genomic DNA was subjected to library construction using the SMRTbell Template Prep Kit (Pacific Biosciences of California, Inc., United States) and sequenced using a PacBio RS II or PacBio Sequel II.

For Illumina short-read sequencing, the cells were harvested by centrifugation at 2,000 × *g* for 5 min from 2 mL cultures (OD_750_ ∼5.0). Genomic DNA was extracted from the harvested cells according to Hirooka et al. (2022). The extracted genomic DNA was separately subjected to library construction using the MiSeq Reagent Kit ver. 3 (Illumina, Inc.) for the 300 bp paired-end library and TruSeq DNA PCR-Free Kit (Illumina, Inc.) for the 150 bp paired-end library. The 300 and 150 bp paired-end libraries were sequenced using MiSeq and NovaSeq 6000, respectively. The sequence reads were processed using Cutadapt ver. 3.1 (Martin 2011) to remove low-quality ends (<QV30), adapter sequences, and reads shorter than 50 bp. The trimmed reads were used for subsequent analyses.

### Genomic analyses

For de novo assembly of organelle genomes, Illumina short reads of the haploid clones (*Cc. yangmingshanensis* NIES-4659 Ha1, *Cd. caldarium* NIES-551 Ha5) were assembled using SPAdes ver. 3.15.0 (Bankevich et al. 2012). Genes of the chloroplast and mitochondrial genomes were annotated using GeSeq on Chlorobox (Tillich et al. 2017), with the previously annotated mitochondrial and chloroplast genomes (Cho et al. 2020; Park et al. 2023) as references. For de novo nuclear genome assembly, PacBio long reads of the haploid clones (*Cc. yangmingshanensis* NIES-4659 Ha1, *Cd. caldarium* NIES-551 Ha5, and *Cz. merolae* MS1 Ha3) were mapped to the chloroplast and mitochondrial genomes using Minimap2 ver. 2.17 (Li, 2018), and the mapped reads were removed by BEDTools (Quinlan and Hall 2010). The remaining long reads were then assembled de novo using Canu ver. 2.0 (Koren et al. 2017). The assembled contigs were error corrected using Pilon ver. 1.2.3 (Walker et al. 2014), with Illumina reads mapped using Bowtie2 ver. 2.3.4.1 (Langmead and Salzberg 2012). Nuclear genes were predicted using AUGUSTUS (Stanke et al. 2004) with species-specific parameters trained on manually curated gene models for *Cyanidiococcus* and *Cyanidium*, integrating RNA-seq data from both diploid and haploid cells, followed by additional manual curation. Each translated amino acid sequence was used as a BLASTP query against the nr database (E-value of <1 × 10^−3^) through Blast2GO (Conesa et al. 2005) to assign provisional annotations (Data Set S7). Nuclear genes encoding TFs, TRs, and protein kinases (including MAPK components) were identified using iTAK (Zheng et al. 2016). However, in *Cd. caldarium*, the *BELL*-related TF gene was not identified by iTAK but was detected through manual curation. Secretory proteins, glycosyltransferases, and transmembrane proteins were identified using SignalP 6.0 (Teufel et al. 2022), dbCAN2 metaserver (Zhang et al. 2018), and the Phobius web server (Kall et al. 2007), respectively. In addition, these sequences were subjected to the SUPERFAMILY database ver. 1.75 (Wilson et al. 2007) to verify the presence of specific domains (Sec1/munc18-like, BAR/IMD, Kelch, trefoil, and hedgehog). SNARE, ESCRT-III, and actin-related proteins were identified by BLAST searches using annotated sequences as queries (Sanderfoot 2007; Kusdian et al. 2013; Yagisawa et al. 2020). Gamete fusion and meiotic tool genes were also identified by BLAST searches. *A. thaliana* genes were used as queries for gamete fusion–related genes (Mori et al. 2006; Alandete-Saez et al. 2011), and *Cz. merolae* 10D and *Galdieria javensis* 074W genes were used as queries for meiotic tool genes (Thangavel et al. 2023).

For comparative genome analysis, genome datasets downloaded from public databases (Data Set S4) were used. To identify orthogroups, gene clustering analysis was performed using OrthoFinder ver. 2.5.4 (Emms and Kelly 2019) with default parameters. A BLASTP search for amino acid sequences was performed using DIAMOND ver. 2.1.0 (Buchfink et al. 2015).

To identify SNPs and indels and estimate genome-wide SNP/indel rates, DNA-seq reads were mapped to the reference genome sequences (*Cc. yangmingshanensis* NIES-4659 Ha1, *Cd. caldarium* NIES-551 Ha5, and *Cz. merolae* MS1 Ha3) using Bowtie2 ver. 2.3.4.1 (Langmead and Salzberg 2012). Callable sites, defined as genomic positions with sufficient coverage and mapping quality, were determined separately for each strain using GATK CallableLoci ver. 3.8 with a minimum mapping quality (MAPQ) threshold of 20 to limit the contribution of ambiguously or multiply mapped reads. Noncallable sites (LOW_COVERAGE, NO_COVERAGE, or POOR_MAPPING_QUALITY) were identified and merged for each strain. For each genus, the union of noncallable sites across all strains was subtracted from the reference genome to define the final set of callable sites shared among strains. Callable regions shorter than 200 bp were excluded after subtraction. SNPs and indels were called using GATK HaplotypeCaller ver. 3.8 (McKenna et al. 2010), with variant calling restricted to the callable sites defined above using the -L option. For diploid strains, the default ploidy setting (ploidy = 2) was used, whereas for haploid strains, the ploidy parameter was set to 1 (-ploidy 1). The resulting variant call format (VCF) files were used for subsequent analyses. SNPs and indels were visualized using the Integrative Genomics Viewer (IGV) ver. 2.8.2 (Robinson et al. 2011). Genome-wide SNP/indel rates (%) were calculated as (number of variant sites/total number of callable sites) × 100. For diploid strains, SNP/indel rates were calculated using heterozygous variant sites, whereas for haploid strains, rates were calculated using homozygous variant sites. The number of variant sites was obtained from filtered VCF files.

For *k*-mer analysis, whole-genome Illumina short-read data were processed to calculate a 41-mer frequency distribution using Jellyfish ver. 2.3.0. *k*-mer–based heterozygosity was subsequently estimated using GenomeScope (Vurture et al. 2017).

### Pulsed-field gel electrophoresis

Cells of *Cd. caldarium* NIES-551 Ha5, *Cc. yangmingshanensis* NIES-4659 Ha1, and *Cz. merolae* 10D were harvested by centrifugation at 2,000 × *g* for 5 min at room temperature. The resulting cell pellets were resuspended in 50 *μ*L of cell suspension buffer (10 mM Tris-HCl, pH 7.2, 50 mM EDTA, 20 mM NaCl). Next, 50 *μ*L of 2% Certified Low Melt Agarose (Bio-Rad), prepared in 0.5× TBE buffer (44.5 mM Tris, 44.5 mM boric acid, 1 mM EDTA at pH 8.0), was added to each suspension, gently mixed, and poured into disposable plug molds (Bio-Rad) to solidify at room temperature. The solidified agarose gels (plugs) were incubated in *N*-lauroylsarcosine-Na buffer (1% *N*-lauroylsarcosine-Na, 500 mM EDTA, pH 8.0) with 5 mg proteinase K (Sigma-Aldrich) at 50 °C overnight. The plugs were washed with 0.5× TBE buffer before pulsed-field gel electrophoresis (PFGE). For each sample, 2 mm of plugs were loaded into the wells of 1% PFGE-certified agarose (Bio-Rad) gels prepared in 0.5× TBE buffer. Electrophoresis was run at 6 V cm^−1^ at 14 °C with 120° pulse angle for 15 h with a switch time of 60 s and followed 9 h at a switch time of 90 s using the CHEF-DR III (Bio-Rad) system. Gels were stained with GelRed Nucleic Acid Gel Stain (Biotium), and signals were detected using a ChemiDoc Touch Imaging System (Bio-Rad).

### Preparation of linear DNA for the transformation

The sequences of the primers and all plasmids used in this study are listed in Data Set S6. PCR amplification for plasmid construction and purification of PCR products were performed using PrimeSTAR Max DNA Polymerase (Takara Bio Inc., Japan) and the QIAquick PCR Purification Kit (QIAGEN, Venlo, Netherlands), respectively.

For the transformation of *Cc. yangmingshanensis* NIES-4659 Ha1, we constructed the following plasmids. To integrate the *Venus* expression cassette and the chloramphenicol acetyltransferase (*CAT*) selectable marker into the intergenic region between the g478.t1 and g479.t1 loci (IG1) through homologous recombination, plasmid pCcIG1-Venus-CAT was constructed (Fig. 5b). The *Venus* ORF (the nucleotide sequence was codon optimized for the *Cz. merolae* nuclear genome) and the *CAT* ORF from *Staphylococcus aureus* (UniProtKB/Swiss-Prot ID: P00485; the nucleotide sequence was codon optimized for the *Cz. merolae* nuclear genome) were commercially synthesized. The *Venus* ORF was connected with the *Cc. yangmingshanensis APCC* promoter (*pAPCC*; 500 bp upstream of the *APCC* ORF) and the *Cc. yangmingshanensis β-TUBULIN* terminator (*tTUBB*; 250 bp downstream of the *β-TUBULIN* ORF). The *CAT* ORF was connected with the *Cc. yangmingshanensis ELONGATION FACTOR 1α* promoter (*pEF1α*; 500 bp upstream of the *EF1α* ORF) and the *Cc. yangmingshanensis UBIQUITIN* terminator (*tUBQ*; 250 bp downstream of the *UBIQUITIN* ORF).

To generate a *Cc. yangmingshanensis* transformant in which the *CAT* selectable marker and the *HSVtk* suicide marker, linked by a glycine–serine (GS) linker, are integrated into the IG1 locus, plasmid pCcIG1-tUBQ-CAT-HSVtk-tUBQ was constructed (Fig. 5g). The *HSVtk* ORF (UniProtKB/Swiss-Prot ID: P13157; the nucleotide sequence was codon optimized for the *Cz. merolae* nuclear genome) was commercially synthesized. The *CAT–HSVtk* fusion ORF was connected with *pEF1α*. To generate a uracil-auxotrophic (Δ*URA5.3*) chloramphenicol-resistant *Cc. yangmingshanensis* transformant (Fig. 6a), plasmid pCcΔURA5.3-Venus-CAT was constructed (Fig. S7a).

For the transformation of *Cd. caldarium* NIES-551 Ha5, we constructed the following plasmids. To integrate the *Venus* expression cassette and the *BSD* selectable marker into the intergenic region between the g620.t1 and g621.t1 loci (IG1) through homologous recombination, plasmid pCdIG1-Venus-BSD was constructed (Fig. S5a). The *Venus* ORF (codon optimized for the *Cd. caldarium* nuclear genome) and the *BSD* ORF from *Aspergillus terreus* (UniProtKB/Swiss-Prot ID: P0C2P0.1; codon optimized for the *Cd. caldarium* nuclear genome) were commercially synthesized. The *Venus* ORF was connected with the *Cd. caldarium APCC* promoter (*pAPCC*; 500 bp upstream of the *APCC* ORF) and the *Cd. caldarium β-TUBULIN* terminator (*tTUBB*; 250 bp downstream of the *β-TUBULIN* ORF). The *BSD* ORF was connected with the *Cd. caldarium ELONGATION FACTOR 1α* promoter (*pEF1α*; 500 bp upstream of the *EF1α* ORF) and the *Cd. caldarium UBIQUITIN* terminator (*tUBQ*; 250 bp downstream of the *UBIQUITIN* ORF). To generate a uracil-auxotrophic (Δ*URA5.3*), blasticidin S-resistant *Cd. caldarium* transformant, plasmid pCdΔURA5.3-Venus-BSD was constructed (Fig. S7a).

For the transformation of *Cz. merolae* 10D, we constructed the following plasmids. To generate a *Cz. merolae* transformant in which the *Venus* expression cassette, the *CAT* selectable marker, and the *HSVtk* suicide marker are integrated into the intergenic region (IG1) upstream of the *URA5.3* gene locus, plasmid pCzURAup- Venus-HSVtk-CAT-URAup was constructed (Fig. S5g). The *Venus* ORF was connected with the *Cz. merolae APCC* promoter (*pAPCC*; 600 bp upstream of the *APCC* ORF) and the *Cz. merolae β-TUBULIN* terminator (*tTUBB*; 197 bp downstream of the *β-TUBULIN* ORF). The *HSVtk* ORF was connected with the *Cz. merolae CAB* promoter (*pCAB*; 589 bp upstream of the *CAB* ORF) and the *Cz. merolae APX* terminator (*tAPX*; 420 bp downstream of the *APX* ORF). The *CAT* ORF, fused to a sequence encoding a chloroplast transit peptide (the N-terminal 60 amino acids of *Cz. merolae APCC*), was connected with the *Cz. merolae CPCC* promoter (*pCPCC*; 500 bp upstream of the *CPCC* ORF) and the *Cz. merolae UBIQUITIN* terminator (*tUBQ*; 275 bp downstream of the *UBIQUITIN* ORF). To generate a uracil-auxotrophic (Δ*URA5.3*), chloramphenicol-resistant *Cz. merolae* transformant, plasmid pCzΔURA5.3-Venus-CAT was constructed (Fig. S7a). The *Venus* ORF was connected with the *Cz. merolae APCC* promoter (*pAPCC*; 500 bp upstream of the *APCC* ORF) and the *Cz. merolae β-TUBULIN* terminator (*tTUBB*; 250 bp downstream of the *β-TUBULIN* ORF). The *CAT* ORF was connected with the *Cz. merolae ELONGATION FACTOR 1α* promoter (*pEF1α*; 500 bp upstream of the *EF1α* ORF) and the *Cz. merolae UBIQUITIN* terminator (*tUBQ*; 250 bp downstream of the *tUBQ* ORF).

Linear DNA used for transformation was prepared by PCR with the primer set pUC19F and pUC19R (designed based on the pUC19 sequence), using the respective plasmids as templates. PCR amplification was performed using PrimeSTAR Max DNA Polymerase (Takara Bio Inc.). PCR products were purified using the QIAquick PCR Purification Kit (QIAGEN) and eluted with 55 *μ*L of sterile water, of which 45 *μ*L was used for transformation.

### Transformation

PEG-mediated transformation of haploid clones of *Cc. yangmingshanensis* NIES-4659 Ha1, *Cd. caldarium* NIES-551 Ha5, and *Cz. merolae* 10D was performed according to Fujiwara and Ohnuma (2018), with the following modifications.

To prepare cells for transformation, haploid cells of *Cc. yangmingshanensis* NIES-4659 Ha1 and *Cz. merolae* 10D were inoculated into 20 mL of MA medium at pH 2.0, while haploid cells of *Cd. caldarium* NIES-551 Ha5 were inoculated into 20 mL of MA medium at pH 0.75, each in a 25 cm^2^ tissue culture flask, to give an OD_750_ of 0.4. The cells were then cultured at 42 °C (for *Cc. yangmingshanensis* and *Cz. merolae*) or 37 °C (for *Cd. caldarium*) under a 12-h light/12-h dark cycle (50 *μ*mol photons m^−2^ s^−1^) on a reciprocal shaker at 140 rpm (NR-2; TAITEC). At the end of the third light period, when the percentage of S-phase cells reached its peak (Fujiwara et al. 2020; Jong et al. 2021), Tween-20 was added to the culture to a final concentration of 0.001%. The cells were harvested by centrifugation at 2,000 × *g* for 5 min at room temperature. The cells were then resuspended in IMA medium at pH 1.2 (or pH 0.75 for *Cd. caldarium*) to adjust the OD_750_ to 100. To prepare ∼60% (wt/vol) PEG solution, 0.3 g of PEG4000 (Sigma-Aldrich) was dissolved in 225 *μ*L of MA2 medium (Ohnuma et al. 2008) at 95 °C for 5 min and then kept at 42 °C on a heat block until use. Next, 45 *μ*L of DNA solution (3 to 5 *μ*g of linear DNA), 5 *μ*L of 10× transformation solution (400 mM [NH_4_]_2_SO_4_, 40 mM MgSO_4_, 0.3% H_2_SO_4_), and 62.5 *μ*L of PEG solution were mixed by pipetting in a 1.5 mL tube, yielding a total volume of 112.5 *μ*L. Then, 12.5 *μ*L of cell suspension was added to the 112.5 *μ*L transformation-DNA-PEG mixture (final PEG concentration ∼30% [wt/vol]). The tube was vigorously inverted 10 times, and the contents were immediately transferred to 10 mL of IMA medium at pH 1.2 (or pH 0.75 for *Cd. caldarium*) in 1 well of a 6-well plate (VTC-P6, VIOLAMO). For Δ*URA5.3* transformants, the media were supplemented with 0.5 mg/mL uracil (Sigma-Aldrich). The cells were cultured statically in a 2% CO_2_ incubator at 42 °C (or 37 °C for *Cd. caldarium*) under continuous light (30 *μ*mol photons m^−2^ s^−1^) for 3 d. After incubation, the cells were harvested by centrifugation at 2,000 × *g* for 5 min at room temperature. The cell pellet was resuspended in 1 mL of IMA medium at pH 1.2 (or pH 0.75 for *Cd. caldarium*). A 100 *μ*L aliquot of the cell suspension was inoculated into 1 mL of IMA medium at pH 1.2 supplemented with 100 *μ*g/mL CP (or IMA medium at pH 0.75 supplemented with 1 mg/mL BS for *Cd. caldarium*). For Δ*URA5.3* transformants, the media were supplemented with 0.5 mg/mL uracil and 5-FOA (FUJIFILM Wako). The cells were cultured statically in a 2% CO_2_ incubator at 42 °C (or 37 °C for *Cd. caldarium*) under continuous light (30 *μ*mol photons m^−2^ s^−1^) for 3 wk to allow drug-resistant transformants to grow. Single clones were obtained by limiting dilution of the culture in a 96-well plate (92696, TPP Techno Plastic Products). The Δ*URA5.3* clones were maintained in MA medium at pH 2.0 (or pH 0.75 for *Cd. caldarium*) supplemented with 0.5 mg/mL uracil.

To remove the *HSVtk* suicide marker from a chromosome of a transformant through intrachromosomal homologous recombination, the cells in which the *HSVtk* suicide marker was integrated were inoculated into 1 mL of MA medium at pH 2.0 supplemented with 10 *µ*g/mL 5-FdU (or 20 *µ*g/mL for *Cz. merolae*), in 1 well of a 24-well plate, to give an OD_750_ of 0.2. The cells were then cultured statically in a 2% CO_2_ incubator at 42 °C under continuous light (30 *μ*mol photons m^−2^ s^−1^) for 3 wk to select for cells that had lost the *HSVtk* suicide marker. Single clones were obtained by limiting dilution in a 96-well plate or by plating on cornstarch slurry spots over gellan gum–solidified MA medium.

The occurrence of homologous recombination in each transformant clone was confirmed by PCR using genomic DNA as the template, with the primer sets indicated in Data Set S6. PCR amplification was performed using KOD One PCR Master Mix (TOYOBO Co., Ltd., Osaka, Japan).

### Immunoblotting

Cyanidiophycean cells were harvested by centrifugation at 2,000 × *g* for 5 min at room temperature. The cell pellets were lysed in sample buffer (2% SDS, 62 mM Tris-HCl, pH 6.8, 100 mM DTT, 10% glycerol, and 0.01% bromophenol blue) and incubated at 95 °C for 5 min. The lysate was centrifuged at 15,000 × *g* for 10 min, and the supernatant was collected. Protein concentration was determined using the XL-Bradford assay (Integrale, Japan). A total of 6 *µ*g of protein was separated by SDS-PAGE and transferred to PVDF membranes (Immobilon; Millipore). Membrane blocking, antibody incubation, and signal detection were performed as described by Fujiwara et al. (2024). For the antibodies, an anti-GFP monoclonal antibody (for detection of Venus; dilution 1:2,000; clone JL-8, Takara Bio) and an HRP-conjugated anti-mouse IgG (dilution 1:40,000; Thermo Fisher Scientific) were used.

### Mating experiments and organelle DNA inheritance analysis

To generate and select a heterozygous diploid clone by mating haploids, cells of a Δ*URA5.3* haploid clone and those of a wild-type haploid clone, or cells from a haploid population derived from a diploid clone, were inoculated into 1 mL of MA medium at pH 2.0 (or pH 0.75 for *Cd. caldarium*), supplemented with 0.5 mg/mL uracil, to give an OD_750_ of 0.1 for each (total OD_750_ = 0.2). The cells were cultured statically in 1 well of a 24-well plate, enclosed in an AnaeroPack CO_2_ generator (Mitsubishi Gas Chemical), in an incubator at 42 °C (or 37 °C for *Cd. caldarium*) under 12-h light/12-h dark conditions (50 *μ*mol photons m^−2^ s^−1^) for 7 d (or 10 d for *Cd. caldarium*) to allow haploid cell mating.

For *Cc. yangmingshanensis* and *Cz. merolae*, the cells were harvested by centrifugation at 1,500 × *g* for 5 min and resuspended in 1 mL of MA medium at pH 2.0. Then, 100 *μ*L of the cell suspension was spread onto an HATF Immobilon nitrocellulose membrane (85 mm; Merck Millipore) placed over gellan gum–solidified MA medium at pH 2.0 (9 cm Petri dish, 0.5% gellan gum) supplemented with 100 *μ*g/mL CP (or 250 *μ*g/mL for *Cz. merolae*). The cells were cultured in a 2% CO_2_ incubator at 42 °C under continuous light (30 *μ*mol photons m^−2^ s^−1^) for 2 wk to generate colonies of heterozygous diploid cells, which exhibit CP resistance but not uracil auxotrophy (Fig. 6d; Fig. S8a). Single colonies were picked and inoculated into 1.0 mL of MA medium at pH 2.0. For *Cd. caldarium*, instead of spreading the harvested cells onto an HATF Immobilon nitrocellulose membrane, the cells were resuspended in 1 mL of MA medium at pH 2.0, and 100 *μ*L of the cell suspension was inoculated into 1 mL of MA medium at pH 2.0 supplemented with 1 mg/mL blasticidin S in each well of a 24-well plate. The cells were cultured in a 2% CO_2_ incubator at 37 °C under continuous light (30 *μ*mol photons m^−2^ s^−1^) for ∼1 mo. Single clones were obtained by limiting dilution of the culture in a 96-well plate.

The heterozygosity of the resulting diploid clones was confirmed by PCR (primer sequences are listed in Data Set S6). The heterozygous diploid clones were maintained in MA medium at pH 2.0.

To examine organelle DNA inheritance, DNA was extracted from parental haploid clones and their diploid progeny using the Kaneka Easy DNA Extraction Kit ver. 2 (Kaneka Corp., Tokyo, Japan) with acid-washed glass beads (425 to 600 *μ*m; Sigma-Aldrich). Targeted regions of the mitochondrial and chloroplast DNA were amplified by PCR using specific primer sets (sequences are listed in Data Set S6) and sequenced by Sanger sequencing.

### RNA extraction and RNA-seq analyses

Cells of *Cc. yangmingshanensis* NIES-4659 Ha1 and the homozygous diploid derived from NIES-4659 Ha1 were inoculated into 50 mL of MA medium at pH 2.0 to give an OD_750_ of 0.2 in 100 mL test tubes (IWAKI, Japan). The cells were then cultured with aeration (500 mL ambient air min^−1^) at 40 °C under continuous light (100 *μ*mol photons m^−2^ s^−1^) for 3 d. Cells of other cyanidiophycean strains (*Cz. merolae* MS1, *Cz. merolae* MS1 Ha3, *Cd. caldarium* NIES-551, *Cd. caldarium* NIES-551 Ha5, *G. partita* NBRC 102759, and *G. partita* NBRC102759 N1) were inoculated into 20 mL of MA medium at pH 2.0 for *Cz. merolae*, pH 0.75 for *Cd. caldarium*, and pH 1.0 for *G. partita* to give an OD_750_ of 0.2 in 25 cm^2^ tissue culture flasks. The cells were cultured on a reciprocal shaker at 120 rpm in an incubator at 40 °C for *Cz. merolae* and at 37 °C for *Cd. caldarium* and *G. partita* under ambient air and continuous light conditions (50 *μ*mol photons m^−2^ s^−1^) for 3 d. The cells were harvested by centrifugation at 2,000 × *g* for 5 min, frozen in liquid nitrogen, and stored at −80 °C until use. Total RNA was extracted from the frozen cells according to Hirooka et al. (2022). Strand-specific cDNA libraries were constructed and sequenced by Novogene Co. Ltd. (Beijing, China) using a NovaSeq 6000 with 150 bp paired-end sequencing. The sequence reads were processed using Cutadapt ver. 3.1 (Martin 2011) to remove low-quality ends (<QV30), adapter sequences, and reads shorter than 50 bp. The trimmed reads were used for subsequent analyses.

The RNA-seq paired-end reads were mapped to protein-coding genes using Bowtie2 ver. 2.3.4.1 (Langmead and Salzberg 2012). Strand-specific read counts for each protein-coding gene were calculated using SAMtools ver. 1.8 (Li et al. 2009) with the -f 80 option, BEDtools ver. 2.17.0 bamToBed (Quinlan and Hall 2010), and R ver. 3.2.3. The count data were then normalized between the diploid and haploid cells, and differentially expressed genes (DEGs) were identified using edgeR ver. 3.12.1 (Robinson et al. 2010), with the following criteria: FDR < 0.01, log_2_CPM > 2, and |log_2_FC| > 2. Transcripts per million (TPM) values for each protein-coding gene were calculated according to Li and Dewey (2011).

### Chromatin immunoprecipitation and sequencing

Cells of *Cc. yangmingshanensis* NIES-4659 Ha1 and the homozygous diploid derived from NIES-4659 Ha1 were inoculated into 50 mL of MA medium at pH 2.0 to give an OD_750_ of 0.2 in 100 mL test tubes (IWAKI, Japan). The cells were cultured with aeration (500 mL ambient air min^−1^) at 40 °C under continuous light (100 *μ*mol photons m^−2^ s^−1^) for 3 d. A total of 50 mL of cultured cells were fixed with 1% formaldehyde at 30 °C for 10 min and quenched with 250 mM glycine on ice for 5 min. The fixed cells were harvested by centrifugation at 2,500 × *g* for 5 min and washed twice with ice-cold TBS. The cell pellets were resuspended in extraction buffer [20 mM Tris-HCl, pH 7.5, 150 mM NaCl, 1 mM EDTA, 1% Triton X-100, 0.1% sodium deoxycholate, 0.1% SDS, and a protease inhibitor cocktail {Complete Mini, EDTA-free; Roche Diagnostics}] to give an OD_750_ of 25 for haploid cells and 100 for diploid cells. The cells were disrupted using 0.5 mm low-alkaline glass beads (YGBLA05; Yasui Kikai, Osaka, Japan) in a Multi-Beads Shocker (Yasui Kikai) at 2,700 rpm for 20 cycles (1 min on, 1 min off) at 0 °C. The lysates were transferred to a milliTUBE 1 mL AFA Fiber (Covaris) and sheared using a Covaris S2 Focused-ultrasonicator (Covaris) for 20 min (60 s × 20) at 4 to 6 °C, with a water level of 15, duty cycle of 5%, intensity of 4, and 200 cycles per burst. The lysates were centrifuged at 15,000 × *g* for 10 min, and the supernatant was collected, frozen in liquid nitrogen, and stored at −80 °C until use. A 250 *μ*L aliquot of the chromatin solution was diluted with an equal volume of extraction buffer and incubated with 2 *μ*L of antibody using a Microtube Rotator (MTR-103, AS ONE, Japan) at maximum speed at 4 °C overnight. For the antibodies, an anti-H3K27me3 polyclonal antibody (07-449; Merck) and an anti-histone H3 polyclonal antibody (ab1791; Abcam) were used. Antibody–chromatin mixtures were then incubated with 80 *μ*L of Dynabeads Protein G (Invitrogen in Thermo Fisher Scientific, MA, United States), prewashed twice with 0.5 mL of extraction buffer, using a Microtube Rotator at maximum speed at 4 °C for 4 h. The beads were washed once with 1 mL of extraction buffer, 5 times with 1 mL of LiCl wash buffer (50 mM Tris-HCl, pH 7.5, 0.5 M LiCl, 1 mM EDTA, 1% IGEPAL CA-630, and 0.5% sodium deoxycholate), and once with 1 mL of 50TE buffer (50 mM Tris-HCl, pH 7.5, and 10 mM EDTA). DNA was eluted from the beads by adding 100 *μ*L of 50TE buffer containing 1% SDS and reverse crosslinked at 65 °C overnight. The DNA solutions were treated with 5 *μ*L of PureLink RNase A (Invitrogen) for 1 h at 37 °C, followed by treatment with 1 *μ*L of Proteinase K (FUJIFILM Wako) for 1 h at 50 °C. DNA samples were purified using the Monarch PCR & DNA Cleanup Kit (NEB) and eluted with 20 *μ*L of elution buffer. DNA libraries were constructed and sequenced by Macrogen (Korea) using a NovaSeq 6000 system with 150 bp paired-end sequencing. The sequence reads were processed using Cutadapt ver. 3.1 (Martin 2011) to remove low-quality ends (<QV30), adapter sequences, and reads shorter than 50 bp. The trimmed reads were used for subsequent analyses as ChIP-seq reads.

ChIP-seq reads were mapped to the reference genome sequence of *Cc. yangmingshanensis* NIES-4659 Ha1 using Bowtie2 ver. 2.3.4.1 (Langmead and Salzberg 2012). Mapped reads were counted for each protein-coding gene using SAMtools ver. 1.8 (Li et al. 2009) and BEDtools ver. 2.17.0 (Quinlan and Hall 2010). The count data were normalized between the diploid and haploid cells, and genes that are differentially enriched for H3K27me3 were identified using edgeR ver. 3.12.1 (Robinson et al. 2010), with the following criterion: |logFC| > 2. For visualization, TDF files were generated from BAM files using igvtools (Robinson et al. 2011) and visualized using IGV ver. 2.8.2 (Robinson et al. 2011).

### Proteome analysis

Cells of *Cc. yangmingshanensis* NIES-4659 Ha1 and the homozygous diploid derived from NIES-4659 Ha1 were inoculated into 50 mL of MA medium at pH 2.0 to give an OD_750_ of 0.2 in 100 mL test tubes (IWAKI). The cells were then cultured with aeration (500 mL ambient air min^−1^) at 40 °C under continuous light (100 *μ*mol photons m^−2^ s^−1^) for 3 d. The cells were harvested by centrifugation at 2,000 × *g* for 5 min, frozen in liquid nitrogen, and stored at −80 °C until use. Proteins were extracted from the respective frozen cell samples, hydrolyzed to peptides, and separated and analyzed using an UltiMate 3000 RSLCnano LC system (Thermo Fisher Scientific) and a Q Exactive HF-X mass spectrometer (Thermo Fisher Scientific) by Kazusa Genome Technologies Inc. (Kisarazu, Japan). The identification of peptides and quantitative analysis of the obtained MS data (including cross-run normalization) were conducted using DIA-NN 1.8.1 (Demichev et al. 2020), with both precursor and protein FDRs < 0.01.

### Materials availability

All plasmids and strains generated in this study are available from the lead contact upon request.

## Supplementary Material

koag148_Supplementary_Data

## Data Availability

The nuclear, mitochondrial, and chloroplast genome sequences of *Cc. yangmingshanensis* NIES-4659 Ha1 and *Cd. caldarium* NIES-551 Ha5, as well as the associated raw genomic sequence reads (including those of additional related strains), have been deposited in the DNA Data Bank of Japan (DDBJ)/European Molecular Biology Laboratory (EMBL)/GenBank (BioProject accession nos. PRJDB19695 and PRJDB19696, respectively). The nuclear genome sequences of *Cz. merolae* MS1 Ha3, as well as the associated raw genomic sequence reads (including those of 10D, MS1, and others), have been deposited in DDBJ/EMBL/GenBank (BioProject accession no. PRJDB37388). The RNA-sequencing data of cyanidiophycean algae have been deposited in DDBJ/EMBL/GenBank (BioProject accession no. PRJDB36168). The ChIP-sequencing data of *Cc. yangmingshanensis* have been deposited in DDBJ/EMBL/GenBank (BioProject accession no. PRJDB36311). The Illumina raw genomic sequence reads of *Cc. yangmingshanensis* strains (CcyaKS-1, CcyaKS-5, CcyaKS-11, TS5, TS6, TH2, TH3, and TH12) have also been deposited in DDBJ/EMBL/GenBank (BioProject accession no. PRJDB36240). All other data are included in the manuscript and/or the supporting information.
