# Supplementary material for: Sexual life cycle establishes the unicellular red algae Cyanidiophyceae as a genetically tractable model for eukaryotic evolution
Source: Plant Cell. 2026 Jun 5;38(7):koag148. doi: 10.1093/plcell/koag148 (PMC13353863; doi:10.1093/plcell/koag148)
Supplement: koag148_Supplementary_Data [file koag148_supplementary_data.zip › Supplementary_data_PCR64.pdf]

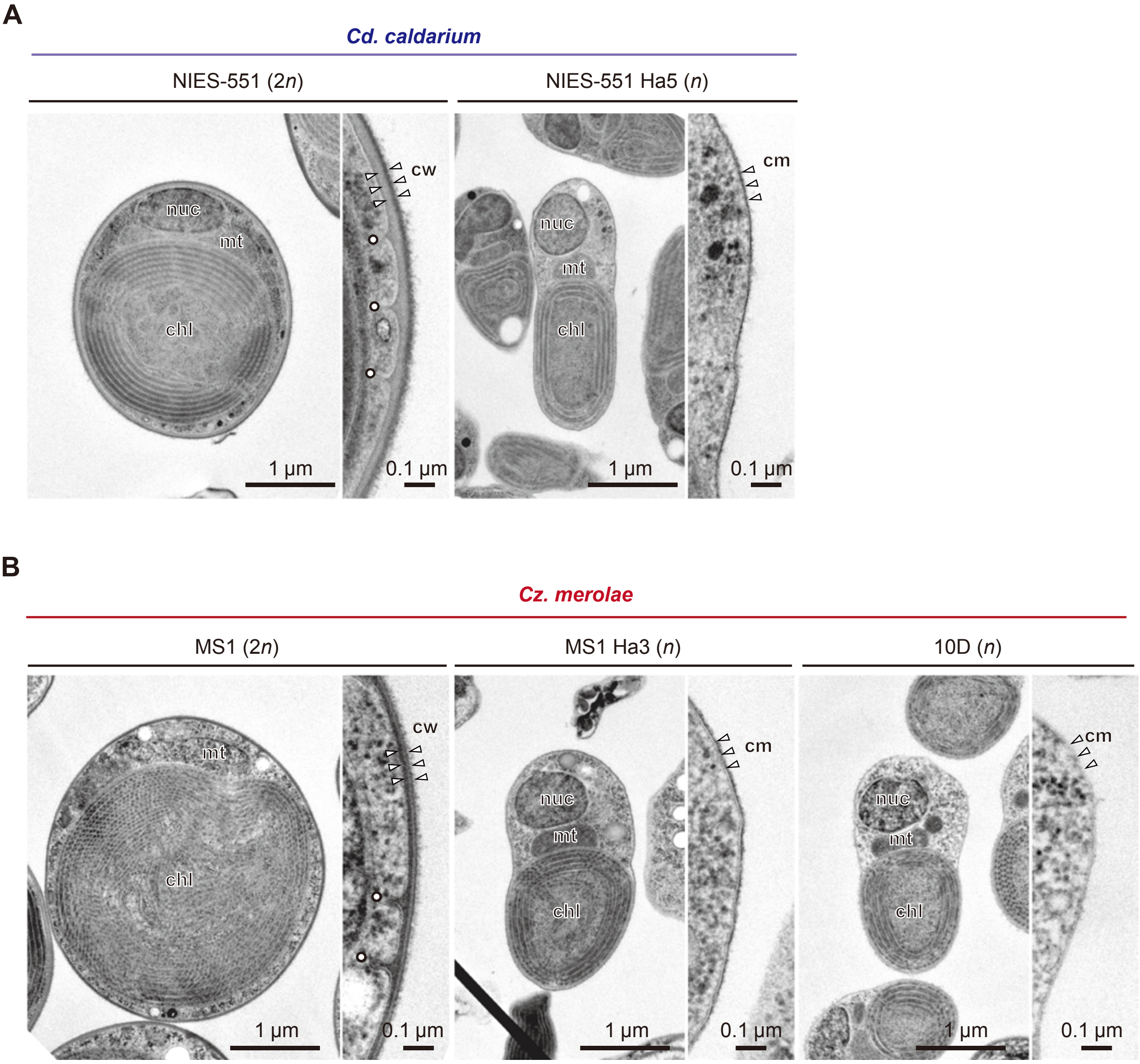

Supplementary Figure S1. Transmission electron micrographs of *Cd. caldarium* and *Cz. merolae*. A) Transmission electron micrographs of the *Cd. caldarium* original 2*n* clone NIES-551 and *n* clone NIES-551 Ha5. **B)** Transmission electron micrographs of the *Cz. merolae* original 2*n* strain MS1 and *n* clones MS1 Ha3 and 10D. *chl*, chloroplast; *cm*, cell membrane; *cw*, daughter cell wall; *mt*, mitochondrion; *nuc*, nucleus; white dots, eisosomes.

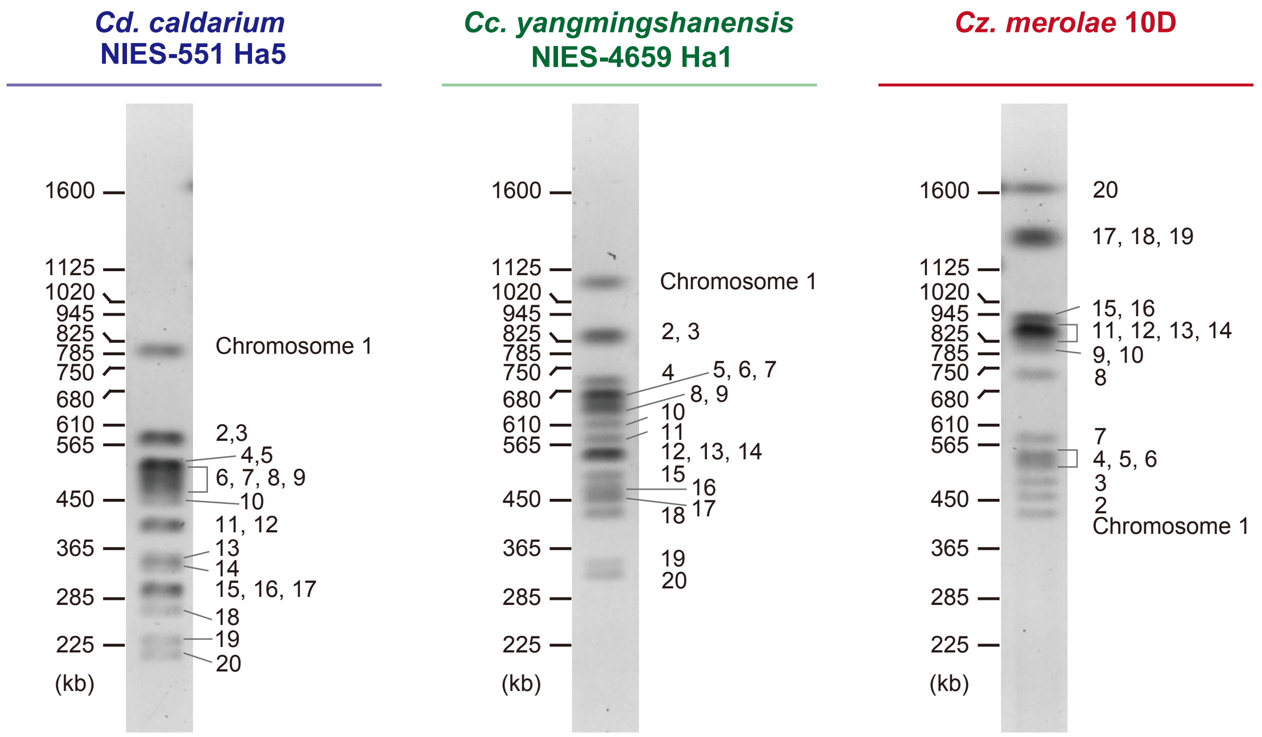

Supplementary Figure S2. Pulsed-field gel electrophoresis separation of chromosomal DNA from haploid clones of *Cd. caldarium*, *Cc. yangmingshanensis*, and *Cz. merolae*.

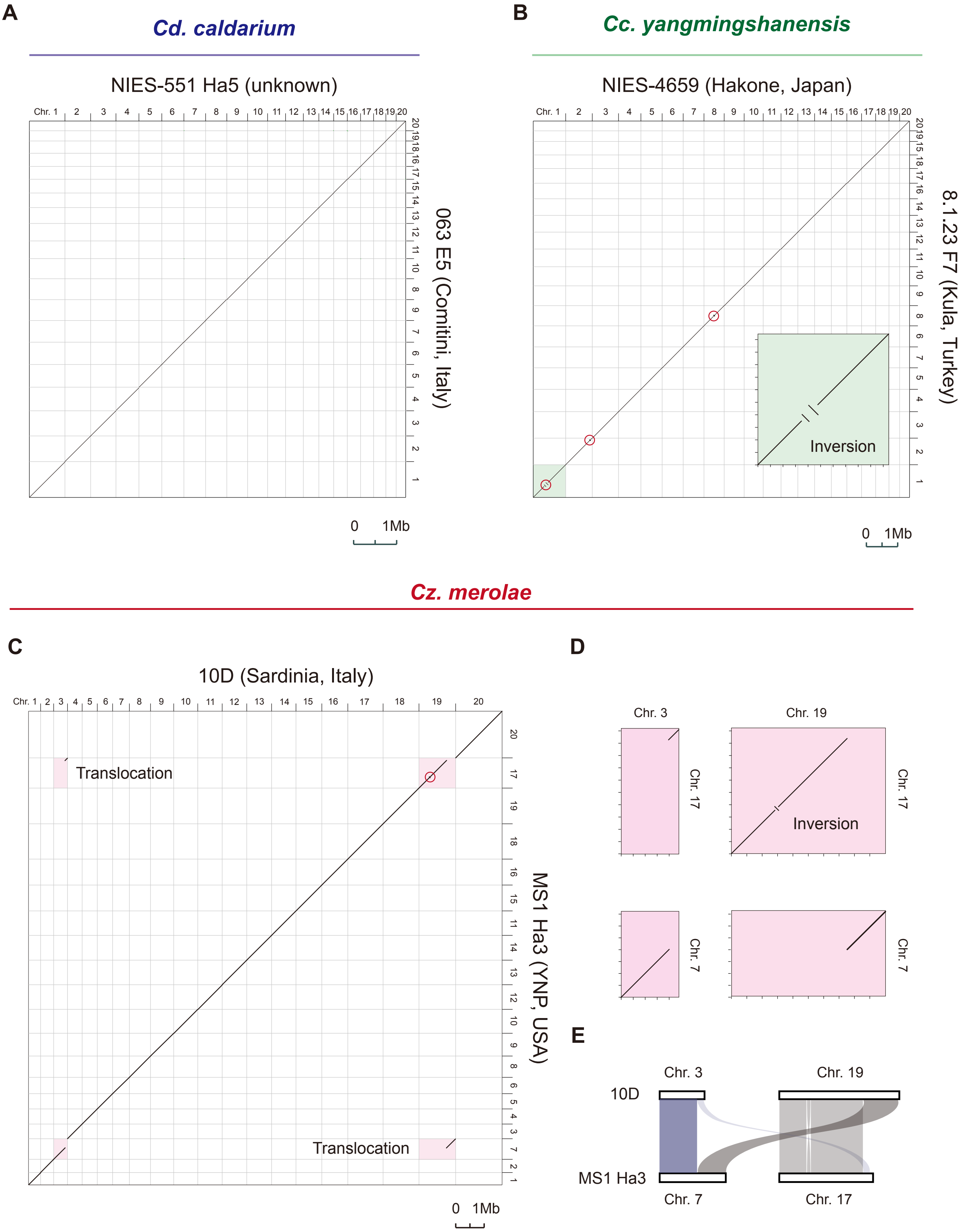

Supplementary Figure S3. Comparison of chromosome-scale synteny between two strains within each cyanidiophycean species. A–C) Whole-genome nucleotide dot plots showing synteny across chromosomes between two strains of *Cd. caldarium* (NIES-551 Ha5 and 063 E5) **(A)**, *Cc. yangmingshanensis* (NIES-4659 Ha1 and 8.1.23 F7) **(B)**, and *Cz.*

merolae (10D and MS1 Ha3) **(C)**, generated using D-Genies (Cabanettes and Klopp, 2018) with its built-in minimap2 aligner. Red circles indicate inversions. Sampling locations are indicated in parentheses following each strain name. In **(B)**, the magnified inset shows two inversions in chromosome 1 (green-highlighted region). **D)** Magnified views of chromosomes 3 and 19 (red-highlighted regions) in **(C)**, showing structural rearrangements. **E)** The genome comparison reveals one reciprocal translocation and one inversion between the 10D and MS1 Ha3 genomes.

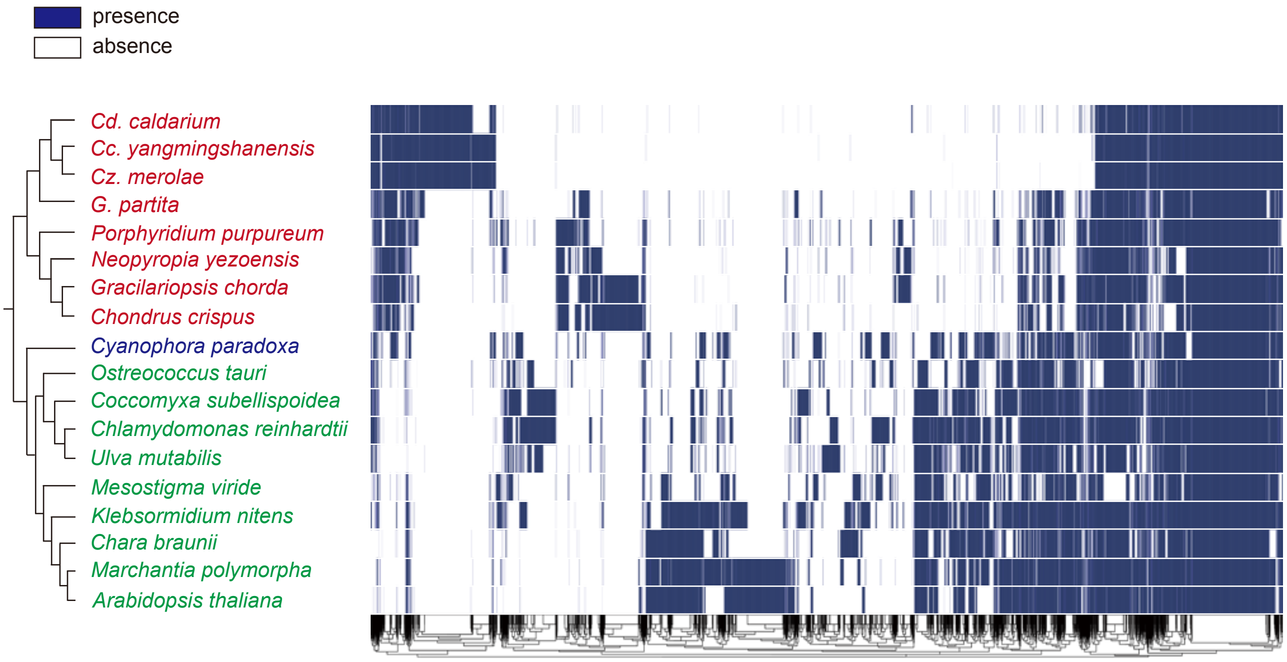

Supplementary Figure S4. Binary heat map showing the distribution of 12,468 orthogroups in 18 members of Archaeplastida, including Cyanidiophyceae. The orthogroups were identified using OrthoFinder (Emms and Kelly, 2019), and species-specific orthogroups were excluded. The clustering of orthogroups was performed using the pheatmap R package ver. 1.0.12 (Kolde, 2019) with default parameters. Columns represent orthogroups, while rows represent species. The dendrogram on the left side of the species names is based on previous studies (Munoz-Gomez et al., 2017; One Thousand Plant Transcriptomes, 2019). The presence of orthogroups is shown in blue, and the absence is shown in white.

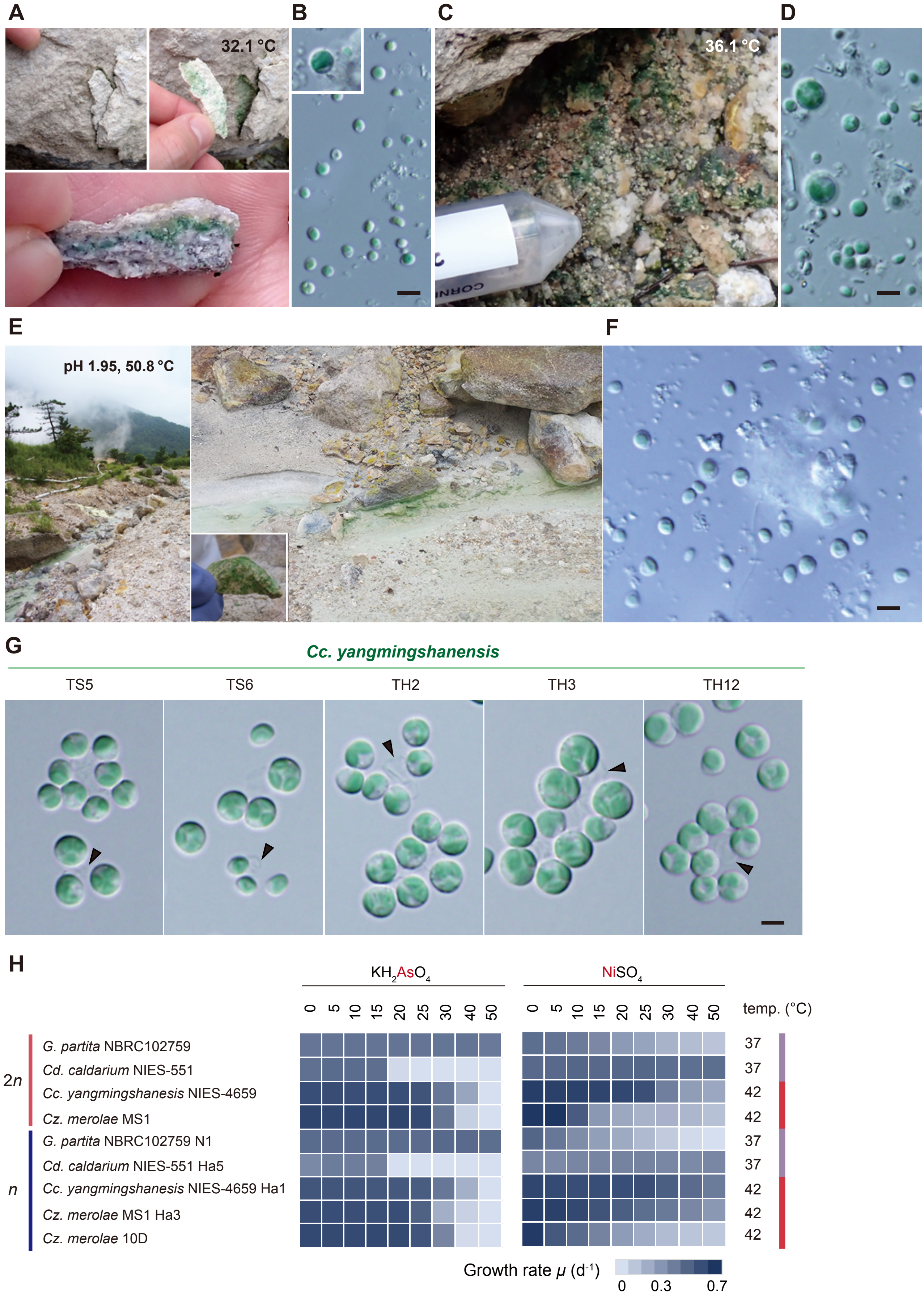
A
32.1 °C
B
C
36.1 °C
D
E
pH 1.95, 50.8 °C
F
G
Cc. yangmingshanensis
TS5
TS6
TH2
TH3
TH12
H
KH2AsO4
NiSO4
0 5 10 15 20 25 30 40 50
0 5 10 15 20 25 30 40 50
temp. (°C)
2n
G. partita NBRC102759
Cd. caldarium NIES-551
Cc. yangmingshanensis NIES-4659
Cz. merolae MS1
n
G. partita NBRC102759 N1
Cd. caldarium NIES-551 Ha5
Cc. yangmingshanensis NIES-4659 Ha1
Cz. merolae MS1 Ha3
Cz. merolae 10D
37
37
42
42
37
37
42
42
42
Growth rate μ (d-1)
0 0.3 0.7

Supplementary Figure S5. Natural habitat and comparison of metal tolerance of cyanidiophycean algae. A) Cyanidiophycean algae inhabiting the endolithic region around an acidic hot spring in Kusatsu, Gunma Prefecture, Japan (only temperature was measured because pH could not be determined). **B)** A DIC micrograph of cell-walled cyanidiophycean algae (smaller algal cells are most probably *Cyanidiococcus* spp.) obtained from an endolithic sample. The inset shows a larger algal cell, rarely observed, that is most probably *Galdieria*. Scale bar: 5 μm. **C)** Cyanidiophycean algae inhabiting the ground above spring water around an acidic hot spring in Kusatsu (only temperature was measured because pH could not be determined). **D)** A DIC micrograph of cell-walled cyanidiophycean algae (smaller algal cells are most probably *Cyanidiococcus* spp. and larger algal cells *Galdieria* spp.) obtained from the ground. Scale bar: 5 μm. **E)** Cyanidiophycean algae inhabiting an acidic hot spring in Tsukahara, Oita Prefecture, Japan, were predominantly found in association with stones in the water. **F)** A DIC micrograph of cell-walled cyanidiophycean algae (all algal cells are most probably *Cyanidiococcus* spp.) obtained from blue-green mats collected in the acidic hot spring. Scale bar: 5 μm. **G)** DIC micrographs of cultured cell-walled *Cc. yangmingshanensis* strains isolated from the acidic hot spring in Tsukahara. The white arrowhead indicates the mother cell wall released upon hatching of daughter cells. Scale bar: 2 μm. **H)** Heatmaps showing the growth rate based on the increase in OD_{750} under the indicated metal concentrations. See also Supplementary Data Set 5 for details.

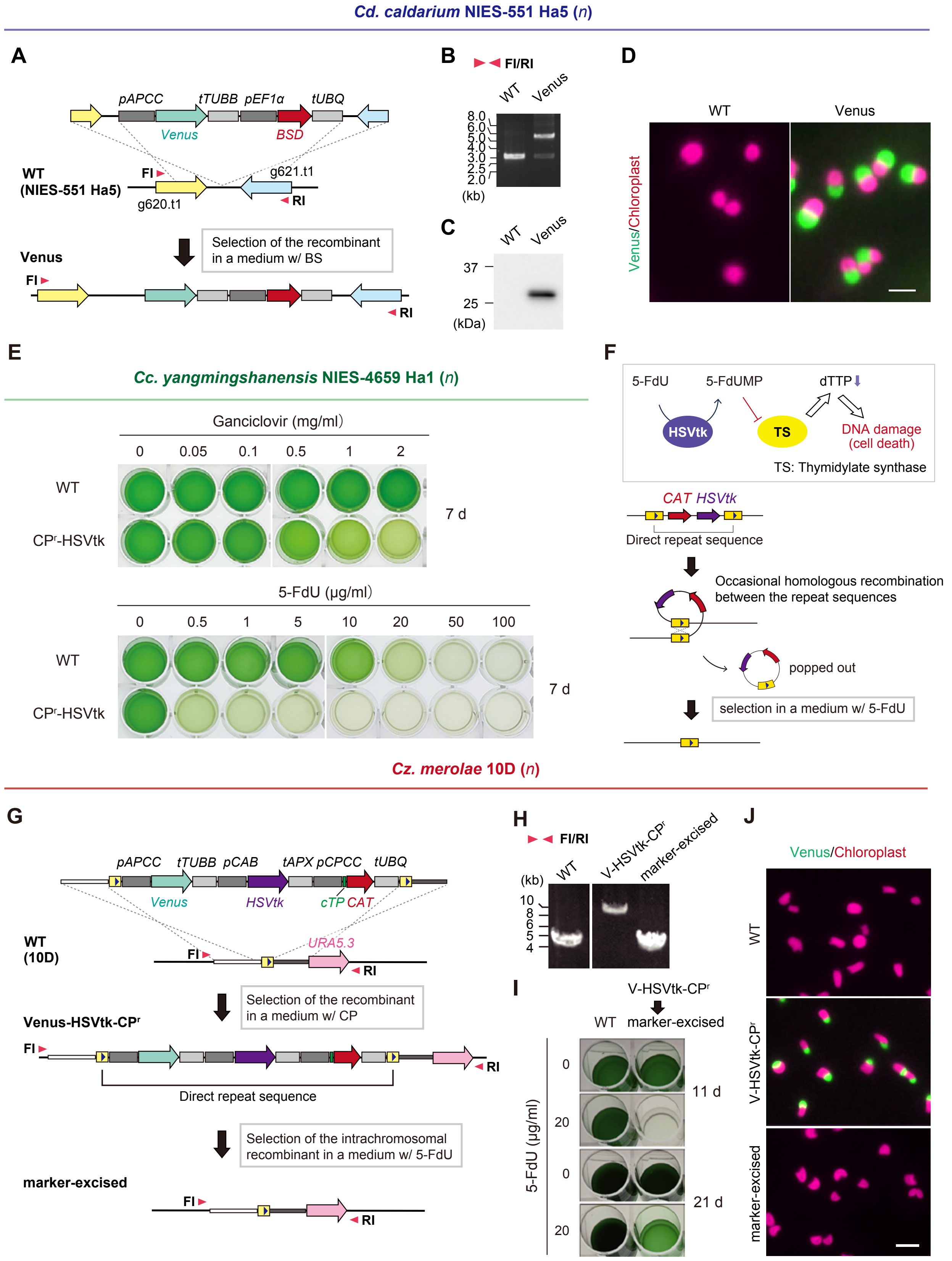

Supplementary Figure S6. Genetic manipulation and marker removal in the cyanidiophycean algae. A) A schematic diagram showing insertion of the *Venus* expression cassette and the *BSD* selectable marker into a chromosomal intergenic region

by homologous recombination in *Cd. caldarium n* clone NIES-551 Ha5. **B)** Targeted integration of the transgenes was confirmed by PCR using primers FI and RI indicated by arrowheads in **A**. **C and D)** Expression of Venus protein was confirmed by immunoblotting **(C)** and by fluorescence microscopy **(D)** (green, Venus fluorescence; magenta, chloroplast fluorescence). WT served as a negative control. Scale bar: 2 µm. **E)** The CP^r-HSVtk and WT *n* cells of *Cc. yangmingshanensis* were cultured for 7 days in the presence of the indicated concentrations of ganciclovir (upper) and 5-FdU (lower). **F)** HSVtk converts 5-FdU into the toxic product 5-FdUMP, which inhibits thymidylate synthase. Cells in which the selectable marker was removed through intrachromosomal homologous recombination between the repeat sequences (indicated by a blue arrowhead in the yellow boxes) can be selected in medium containing 5-FdU. **G)** A schematic diagram showing the targeted integration and subsequent removal of the *CAT* selectable marker in *Cz. merolae* 10D. The *Venus* expression cassette, the *HSVtk* suicide marker, and the *CAT* selectable marker were sandwiched between two directly repeated *URA5.3* upstream sequences (indicated by a blue arrow in the yellow boxes). This construct was integrated into an intergenic region (upstream of the *URA5.3* gene locus) of WT *Cz. merolae* 10D by homologous recombination. After selecting the transformant (V-HSVtk-CP^r) in the presence of CP, the selectable marker was removed through intrachromosomal homologous recombination between the two copies of the repeated sequences, followed by selection with 5-FdU. **H)** The recombination events were confirmed by PCR using the primers FI and RI indicated by arrowheads in **G**. **I)** The V-HSVtk-CP^r cells were cultured for 21 days in the presence or absence of 5-FdU. WT *Cz. merolae* 10D served as a negative control. **J)** Expression of Venus was confirmed by fluorescence microscopy (green, Venus fluorescence; magenta, chloroplast fluorescence). Scale bar: 5 µm.

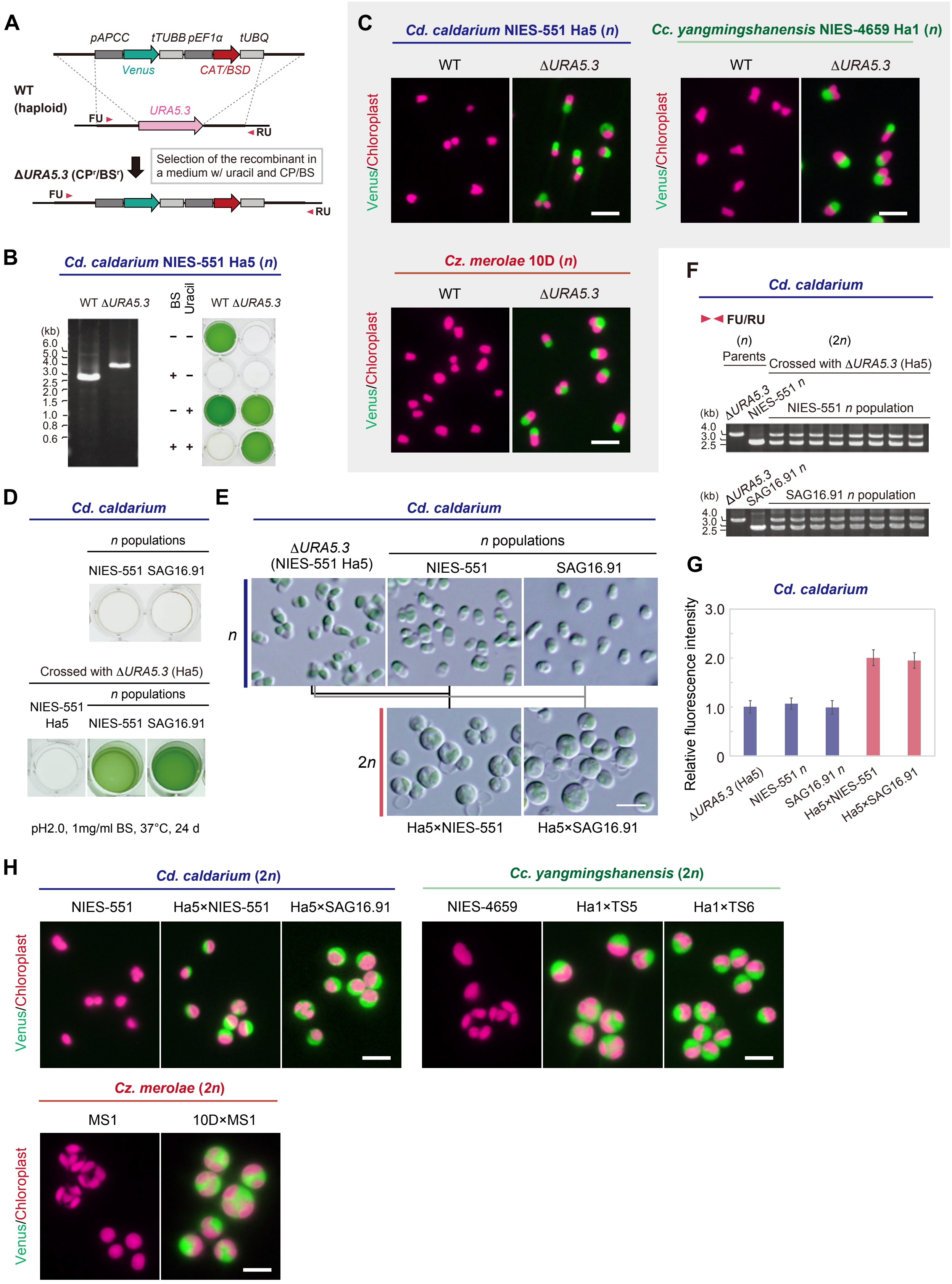

Supplementary Figure S7. Construction of *URA5.3* knockout haploid strains and confirmation of Venus expression in *URA5.3* knockout haploid strains and hybrid diploid clones. A) To select heterozygous 2*n* clones, a uracil-auxotrophic, BS/CP-resistant

n clone was generated. To knockout *URA5.3* and provide BS and CP resistance to *Cd. caldarium* NIES-551 Ha5 and *Cz. merolae* 10D, respectively, the Venus expression cassette and the *BSD* or *CAT* selectable marker were integrated into the chromosomal *URA5.3* locus by homologous recombination. **B)** Replacement of the chromosomal *URA5.3* locus with the Venus expression cassette and the *BSD* selectable marker in the resultant *Cd. caldarium* Δ*URA5.3 n* clone was confirmed by PCR using primers FU and RU, indicated by the arrowheads in **A**. WT *n* clone served as a control. The uracil auxotrophy and BS resistance of the Δ*URA5.3 n* clone were confirmed by cultivation for 7 days in the presence or absence of uracil and BS. WT *n* clone served as controls. **C)** Expression of Venus in the *URA5.3* knockout *n* clones was confirmed by fluorescence microscopy (green, Venus fluorescence; magenta, chloroplast fluorescence). Respective WT clones served as negative controls. Scale bar: 5 µm. **D)** *Cd. caldarium* Δ*URA5.3* (BS^r) *n* clone was crossed with the wild-type NIES-551 clone Ha5 (the parental *n* clone of the Δ*URA5.3* [BS^r] *n* clone) and with *n* populations derived from NIES-551 2*n* and SAG16.91 2*n*. Heterozygous 2*n* clones generated by mating were selected in MA liquid medium at pH 2.0 with 1 mg/ml BS. Respective WT *n* populations served as negative controls. The phylogenetic positions of *Cd. caldarium* NIES-551 and SAG16.91 are shown in Fig. 4E. **E)** DIC micrographs of *Cd. caldarium* clones: Δ*URA5.3* (NIES-551 Ha5) *n* clone, *n* populations derived from the original 2*n* clones NIES-551 and SAG16.91, and hybrid 2*n* clones Ha5×NIES-551 and Ha5×SAG16.91 from their respective combinations. **F)** Heterozygosity of the hybrid 2*n* clones was confirmed by PCR using primers FU and RU. **G)** Nuclear DNA content was compared by measuring fluorescence intensity of DAPI-stained nuclei. The mean fluorescence intensity of Δ*URA5.3 n* cells was defined as 1.0. Data are means ±SD from 20 independent cells. **H)** Venus expression in the hybrid 2*n* clones was confirmed by fluorescence microscopy (green, Venus fluorescence; magenta, chloroplast fluorescence). Respective WT 2*n* clones served as negative controls. Scale bar: 5 µm.

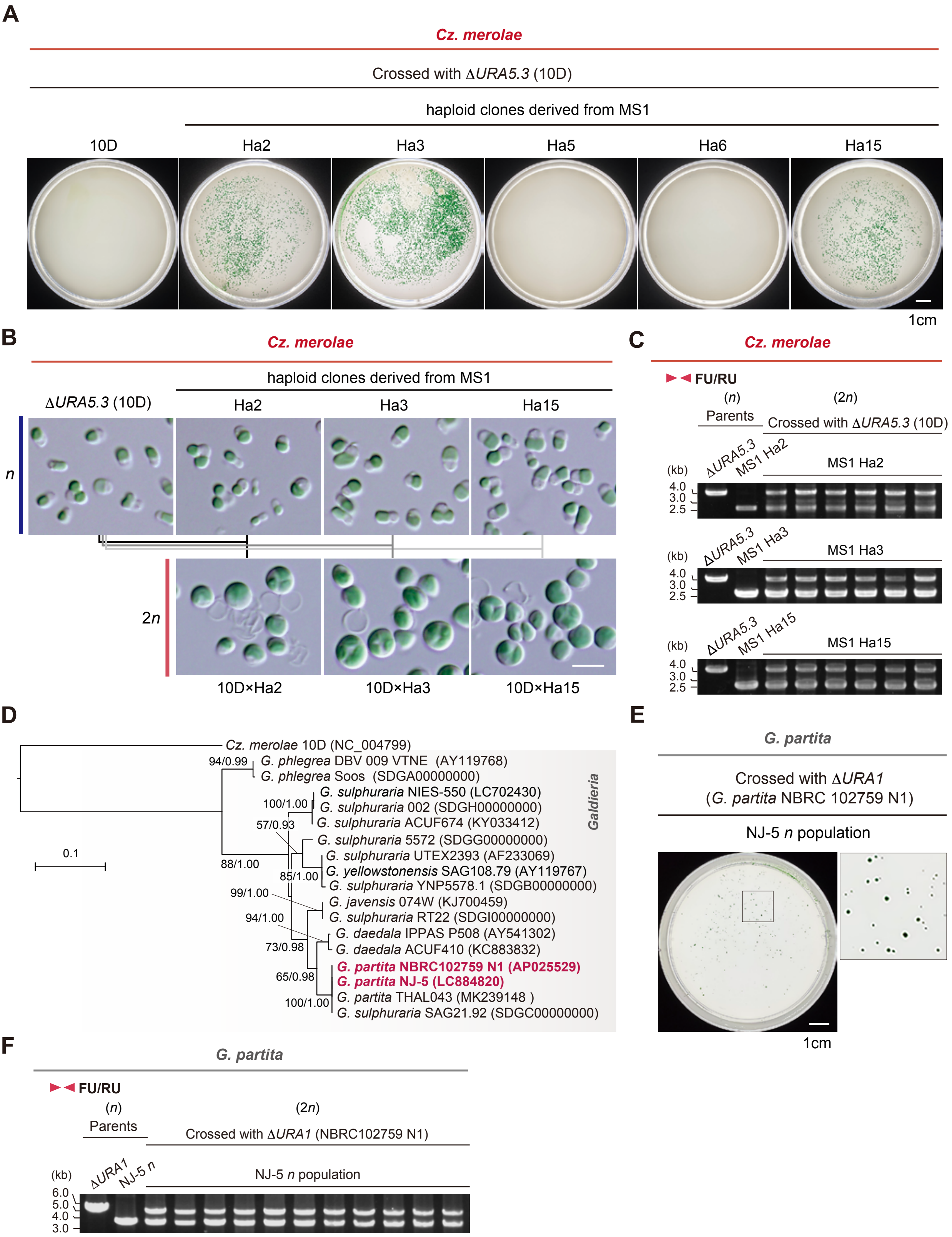

Supplementary Figure S8. Heterozygous diploid generation by haploid mating in *Cz. merolae* and *G. partita*. A) Δ*URA5.3* (CP^r) *n* clone of *Cz. merolae* 10D was crossed with *n* clones derived from the original 2*n* clone MS1, and heterozygous 2*n* clones generated by

their mating were selected on an HATF Immobilon nitrocellulose membrane (85 mm) placed over gellan gum–solidified medium at pH 2.0 with CP. **B)** DIC micrographs of *Cz. merolae* clones: Δ*URA5.3* (10D) and *n* clones derived from the original 2*n* clone MS1, as well as hybrid 2*n* clones 10D×Ha2, 10D×Ha3, and 10D×Ha15 from their respective combinations. Scale bar: 5 μm. **C)** Heterozygosity of the hybrid 2*n* clones was confirmed by PCR using primers FU and RU, as indicated in Supplementary Fig. S7A. **D)** Phylogenetic relationship of several *Galdieria* strains: The tree, based on nucleotide sequences of the chloroplast-encoded *rbcL*, was generated using maximum likelihood analysis (RAxML-NG ver. 1.0.3) (Kozlov et al., 2019). *Cz. merolae* 10D was used as an outgroup. BP >50% (left), obtained using ML, and BI >0.95 (right), calculated using Bayesian analysis (MrBayes ver. 3.2.7) (Ronquist et al., 2012), are indicated above the branches. Branch lengths reflect the evolutionary distances, as indicated by the scale bar. The strains used in this study are highlighted in red. **E)** *G. partita* NBRC 102759 N1 Δ*URA1* (BS^r) *n* clone was crossed with the WT *n* population derived from the original 2*n* clone *G. partita* NJ-5, and heterozygous 2*n* clones were selected on MA gellan gum–solidified medium at pH 2.0 with 100 μg/ml BS. **F)** Heterozygosity of the hybrid 2*n* clones was confirmed by PCR using primers FU and RU.

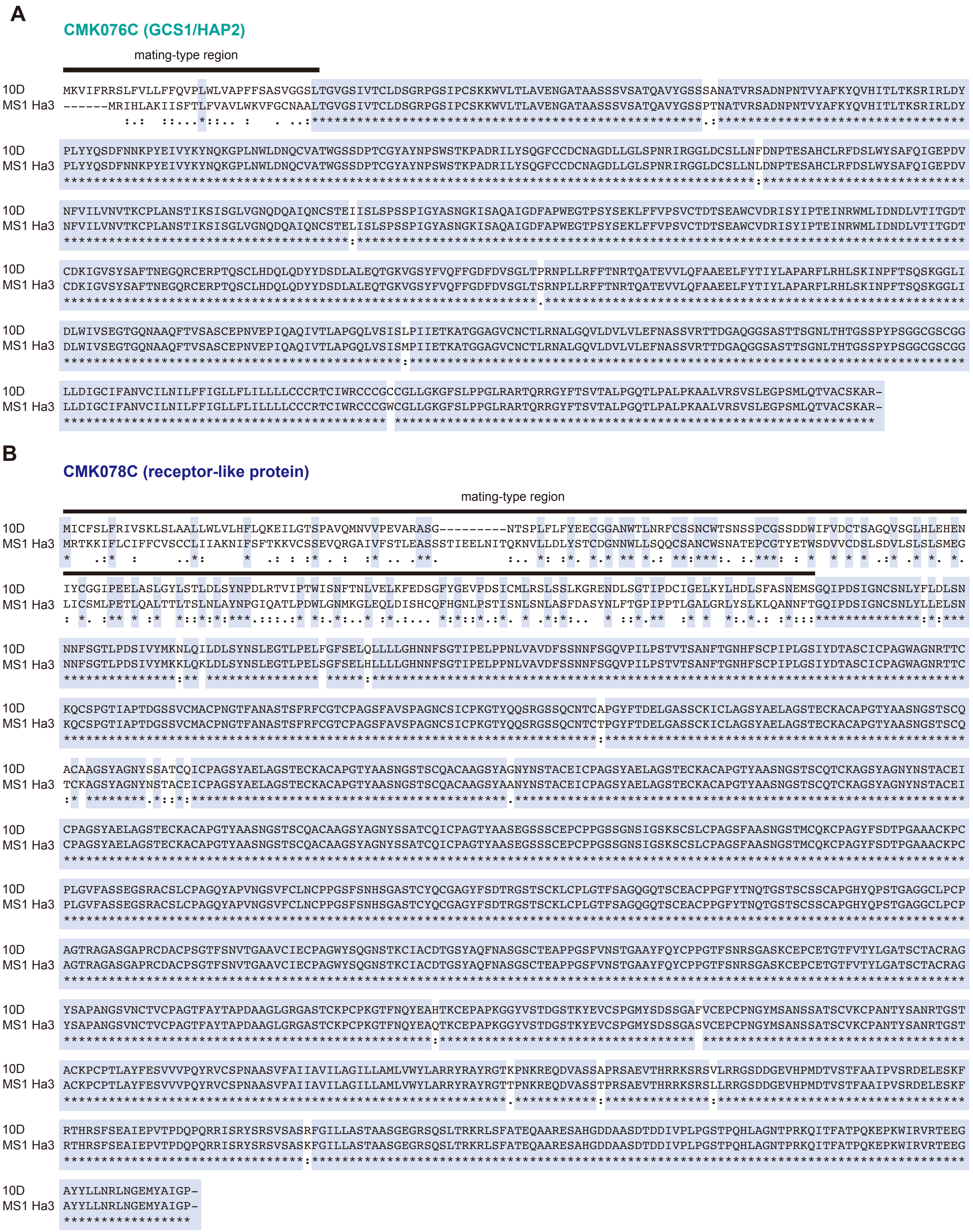

Supplementary Figure S9. Comparison of deduced amino acid sequences of GCS1/HAP2 and receptor-like proteins between *Cz. merolae* 10D and MS1 Ha3. A and B) Deduced amino acid sequence alignments of the GCS1/HAP2 protein **(A)** and the

receptor-like protein **(B)** from *Cz. merolae* 10D and MS1 Ha3, showing divergence in their N-terminal regions caused by nucleotide sequence differences in the mating-type region. The amino acid sequences were aligned using ClustalW ver. 2.1. (Thompson et al., 1994).

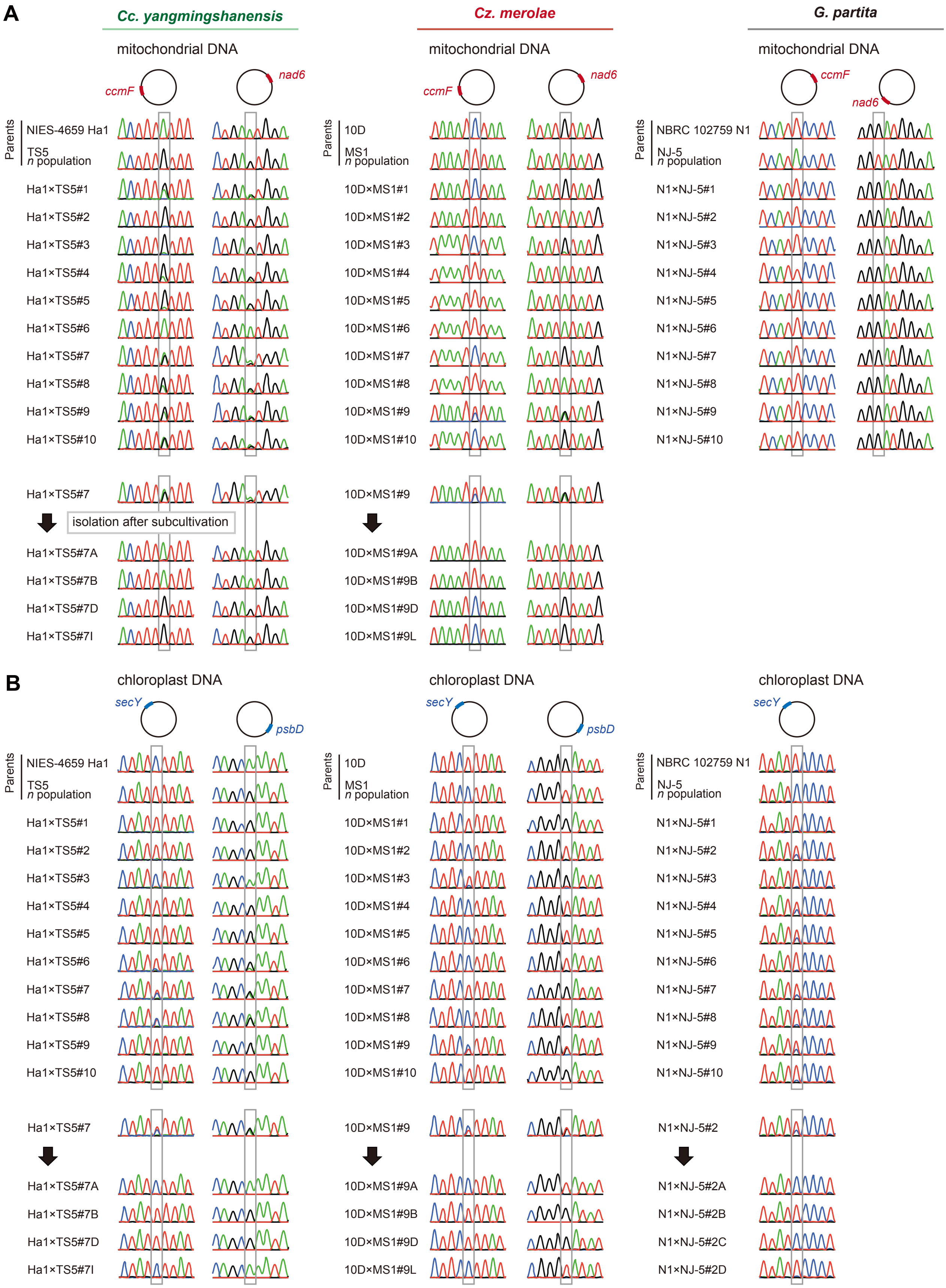
A
Cc. yangmingshanensis
Cz. merolae
G. partita
mitochondrial DNA
ccmF
nad6
Parents
NIES-4659 Ha1
TS5 n population
Ha1×TS5#1
Ha1×TS5#2
Ha1×TS5#3
Ha1×TS5#4
Ha1×TS5#5
Ha1×TS5#6
Ha1×TS5#7
Ha1×TS5#8
Ha1×TS5#9
Ha1×TS5#10
10D
MS1 n population
10D×MS1#1
10D×MS1#2
10D×MS1#3
10D×MS1#4
10D×MS1#5
10D×MS1#6
10D×MS1#7
10D×MS1#8
10D×MS1#9
10D×MS1#10
NBRC 102759 N1
NJ-5 n population
N1×NJ-5#1
N1×NJ-5#2
N1×NJ-5#3
N1×NJ-5#4
N1×NJ-5#5
N1×NJ-5#6
N1×NJ-5#7
N1×NJ-5#8
N1×NJ-5#9
N1×NJ-5#10
isolation after subcultivation
Ha1×TS5#7A
Ha1×TS5#7B
Ha1×TS5#7D
Ha1×TS5#7I
10D×MS1#9A
10D×MS1#9B
10D×MS1#9D
10D×MS1#9L
B
chloroplast DNA
secY
psbD
N1×NJ-5#2A
N1×NJ-5#2B
N1×NJ-5#2C
N1×NJ-5#2D

Supplementary Figure S10. Organelle DNA inheritance in the cyanidiophycean algae. A and B) Sanger sequencing chromatograms of mitochondrial (*ccmF* and *nad6* loci) **(A)** and chloroplast (*secY* and *psaD* loci) **(B)** DNA regions obtained from parental *n* clones and their 2*n* progeny. Gray boxes highlight nucleotide variants between the parental *n* clones. Green = adenine, red = thymine, black = guanine, blue = cytosine. After subculturing the 2*n* clones Ha1×TS5#7 (*Cc. yangmingshanensis*), 10D×MS1#9 (*Cz. merolae*), and N1×NJ-5#2 (*G. partita*), which initially inherited mitochondrial and/or chloroplast DNA from both parents, single colonies were again isolated, and the target region of each colony was examined by Sanger sequencing.

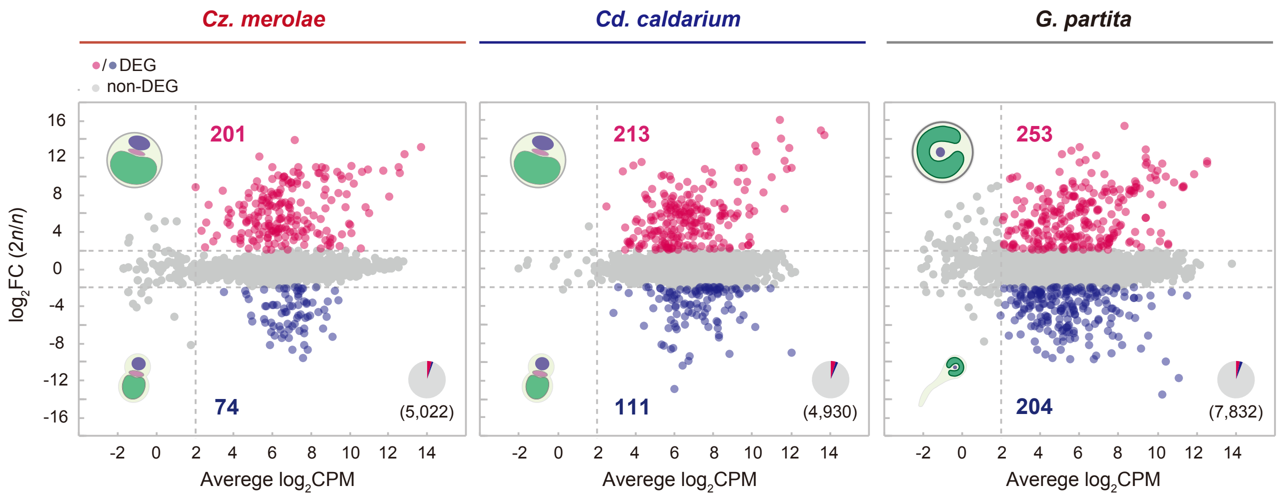

Supplementary Figure S11. Comparison of transcriptomes between diploid and haploid phases in *Cz. merolae*, *Cd. caldarium*, and *G. partita*. These figures are identical to Fig. 7A, except that the results are shown for *Cz. merolae*, *Cd. caldarium*, and *G. partita* rather than *Cc. yangmingshanensis*, and ChIP-seq of H3K27me3 was not conducted.

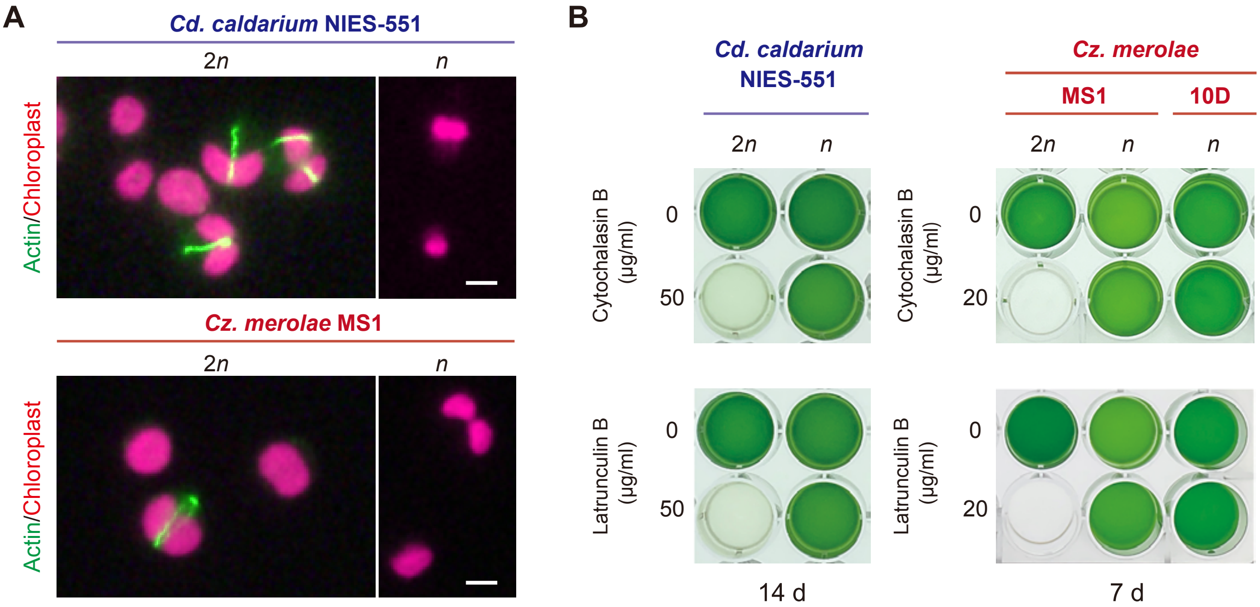

Supplementary Figure S12. Comparison of sensitivity to actin polymerization inhibitors between diploid and haploid cells of *Cd. caldarium* and *Cz. merolae.* A) Actin filaments in 2*n* and *n* cells were visualized with Alexa Fluor^{TM} 488 Phalloidin and observed by fluorescence microscopy; green, Phalloidin fluorescence indicating actin filaments; magenta, chloroplast fluorescence. Scale bar: 2 µm. **B)** Photographs of the 2*n* and *n* cultures after 14 or 7 days of cultivation in the presence or absence of cytochalasin B or latrunculin B.

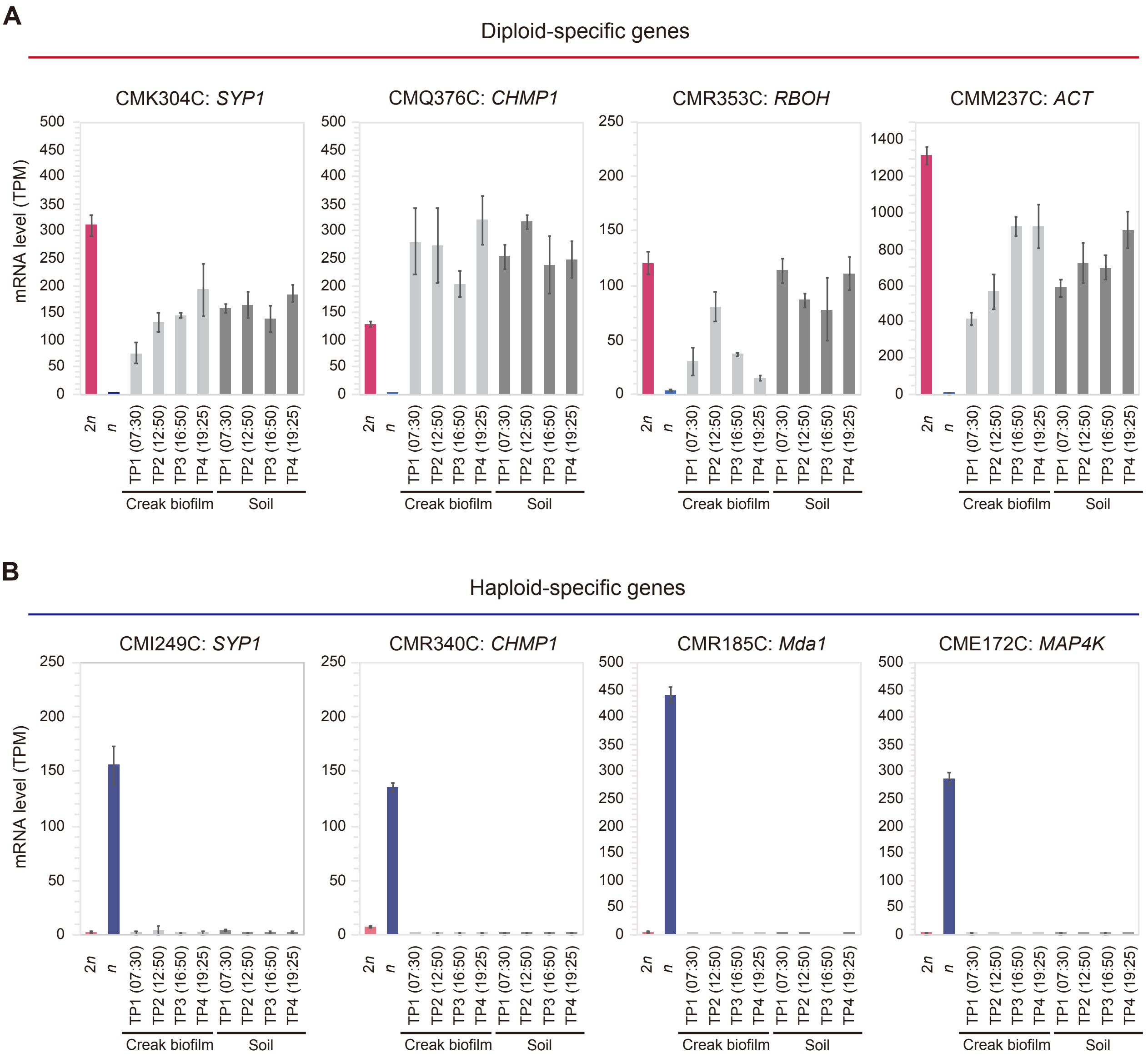

Supplementary Figure S13. Reanalysis of metatranscriptome data from Yellowstone National Park, USA, showing expression patterns of diploid- and haploid-specific genes in *Cz. merolae*. The raw data were obtained from Stephens *et al.* (2024). **A and B)** mRNA levels (TPM) of diploid-specific **(A)** and haploid-specific genes **(B)** from metatranscriptome data of creek biofilms (aquatic) and soil environments (terrestrial; above the water surface) at four time points (TP1–TP4). TPM values of 2*n* and *n* cells were extracted from Supplementary Data Set 8. Data are means ± standard deviations (SDs) from three replicates.

References

Emms, D.M., and Kelly, S. (2019). OrthoFinder: phylogenetic orthology inference for comparative genomics. Genome Biol **20,** 238.

Kolde, R. (2019). Pheatmap: pretty heatmaps. R package version **1,** 726.

Kozlov, A.M., Darriba, D., Flouri, T., Morel, B., and Stamatakis, A. (2019). RAxML-NG: a fast, scalable and user-friendly tool for maximum likelihood phylogenetic inference. Bioinformatics **35,** 4453-4455.

Munoz-Gomez, S.A., Mejia-Franco, F.G., Durnin, K., Colp, M., Grisdale, C.J., Archibald, J.M., and Slamovits, C.H. (2017). The New Red Algal Subphylum Proteorhodophytina Comprises the Largest and Most Divergent Plastid Genomes Known. Curr Biol **27,** 1677-1684 e1674.

One Thousand Plant Transcriptomes, I. (2019). One thousand plant transcriptomes and the phylogenomics of green plants. Nature **574,** 679-685.

Ronquist, F., Teslenko, M., van der Mark, P., Ayres, D.L., Darling, A., Hohna, S., Larget, B., Liu, L., Suchard, M.A., and Huelsenbeck, J.P. (2012). MrBayes 3.2: efficient Bayesian phylogenetic inference and model choice across a large model space. Syst Biol **61,** 539-542.

Thompson, J.D., Higgins, D.G., and Gibson, T.J. (1994). CLUSTAL W: improving the sensitivity of progressive multiple sequence alignment through sequence weighting, position-specific gap penalties and weight matrix choice. Nucleic Acids Res **22,** 4673-4680.
